# Supplementary material for: Which turtle should I study? Uneven distribution of research effort across Testudines species
Source: PLoS One. 2026 Apr 20;21(4):e0347198. doi: 10.1371/journal.pone.0347198 (PMC13094973; doi:10.1371/journal.pone.0347198)
Supplement: S1 File — Phylogeny was included as random effect, and we used the MCMCglmm procedure. Non-significant predictors excluded from the best model (pMCMC > 0.01 or ΔDIC > 5) are shown in italic style below the factors included in the best model. SCL = Straight-line Carapace Length; pm = posterior mean; CI = credibility interval, eff.samp = effective sample size. 110 species were included in this analysis. (DOCX) [file pone.0347198.s001.docx]

Supplementary material 1. Best multi-predictor model explaining variation in research effort across species of Testudinidae after excluding marine species. Phylogeny was included as random effect, and we used the MCMCglmm procedure. Non-significant predictors excluded from the best model (*p*MCMC > 0.01 or ΔDIC > 5) are shown in italic style below the factors included in the best model. SCL = Straight-line Carapace Length; pm = posterior mean; CI = credibility interval, eff.samp = effective sample size. 110 species were included in this analysis.

| Explanatory variables | pm | CI | eff.samp | *p*MCMC |
| --- | --- | --- | --- | --- |
| *Intercept* | 1.985 | [0.439; 3.537] | 1001 | 0.020 |
| Historic range size | 0.673 | [0.391; 0.904] | 868.1 | <0.001 |
| Introduction | 0.479 | [0.023; 0.964] | 1001 | 0.048 |
| Maximum SCL | 0.449 | [0.161; 0.752] | 1001 | 0.002 |
| Egg length | -0.444 | [-0.747; -0.111] | 1001 | 0.008 |
| Biogeographic realm (ΔDIC = -21) |  |  |  |  |
| *Maximum longevity* | *0.183* | *[-0.035; 0.434]* | *754.1* | *0.130* |
| *Insularity* | *0.273* | *[-1.269; 1.734]* | *1001* | *0.721* |
| *Incubation* | *-0.038* | *[-0.299; 0.333]* | *1001* | *0.761* |
| *Country research rank* | *0.010* | *[-0.299; 0.333]* | *1001* | *0.949* |
| *Diet (ΔDIC = 10)* |  |  |  |  |
| *Habitat (ΔDIC = 5)* |  |  |  |  |
