# Supplementary material for: Which turtle should I study? Uneven distribution of research effort across Testudines species
Source: PLoS One. 2026 Apr 20;21(4):e0347198. doi: 10.1371/journal.pone.0347198 (PMC13094973; doi:10.1371/journal.pone.0347198)
Supplement: S4 File — (DOCX) [file pone.0347198.s004.docx]

Supplementary material 4. List of the 32 species with no publication record in the Web Of Science between 1975 and 2023.

| Species | Family |
| --- | --- |
| Chelodina canni | CHELIDAE |
| Chelodina gunaleni | CHELIDAE |
| Chelodina kurrichalpongo | CHELIDAE |
| Chelodina pritchardi | CHELIDAE |
| Chelodina reimanni | CHELIDAE |
| Chelodina steindachneri | CHELIDAE |
| Elseya flaviventralis | CHELIDAE |
| Elseya rhodini | CHELIDAE |
| Emydura gunaleni | CHELIDAE |
| Emydura tanybaraga | CHELIDAE |
| Chelydra acutirostris | CHELYDRIDAE |
| Trachemys hartwegi | EMYDIDAE |
| Trachemys nebulosa | EMYDIDAE |
| Trachemys yaquia | EMYDIDAE |
| Cuora philippinensis | GEOEMYDIDAE |
| Cuora praschagi | GEOEMYDIDAE |
| Cyclemys enigmatica | GEOEMYDIDAE |
| Geoemyda japonica | GEOEMYDIDAE |
| Kinosternon acutum | KINOSTERNIDAE |
| Kinosternon durangoense | KINOSTERNIDAE |
| Kinosternon mexicanum | KINOSTERNIDAE |
| Sternotherus intermedius | KINOSTERNIDAE |
| Pelomedusa kobe | PELOMEDUSIDAE |
| Pelomedusa schweinfurthi | PELOMEDUSIDAE |
| Pelusios adansonii | PELOMEDUSIDAE |
| Pelusios bechuanicus | PELOMEDUSIDAE |
| Pelusios cupulatta | PELOMEDUSIDAE |
| Pelusios marani | PELOMEDUSIDAE |
| Chelonoidis vandenburghi | TESTUDINIDAE |
| Kinixys natalensis | TESTUDINIDAE |
| Cycloderma aubryi | TRIONYCHIDAE |
| Nilssonia leithii | TRIONYCHIDAE |
