# Supplementary material for: Which turtle should I study? Uneven distribution of research effort across Testudines species
Source: PLoS One. 2026 Apr 20;21(4):e0347198. doi: 10.1371/journal.pone.0347198 (PMC13094973; doi:10.1371/journal.pone.0347198)
Supplement: Attachment — Submitted filename: PONE-D-25-36542_R1_reviewer.pdf [file pone.0347198.s006.pdf]

PLOS ONE

Which turtle should I study? Uneven distribution of research effort across Testudines species

--Manuscript Draft--

Manuscript Number:	PONE-D-25-36542R1
Article Type:	Research Article
Full Title:	Which turtle should I study? Uneven distribution of research effort across Testudines species
Short Title:	Research effort in turtles and tortoises
Corresponding Author:	Simon Ducatez<br>Institut de recherche pour le développement: Institut de recherche pour le developpement<br>Montréal, Québec CANADA
Keywords:	Chelonian; research bias; tortoise; turtle
Abstract:	Understanding why some species are more often studied than others can provide valuable information on potential biases that research effort discrepancies can cause and help to direct future research to mitigate these biases. Turtles form an order of reptiles in which the majority of species (67%) are threatened with extinction, but they show high variation in the amount of research devoted to each species. We aimed to quantify this variation across all recognized extant species, and to decipher how this distribution of this research effort varied according to species taxonomy, phylogeny, distribution, ecology, life history and extinction risk. Using the number of references listed on the Web of Science for each species as a proxy for species research effort, we first show that this index is both robust to variation in search methods within WoS, and consistent with an index extracted from another online library (Scopus). The number of articles per species varied substantially, with 3441 articles for the green sea turtle alone, but only 475 total articles for all 50% least studied species taken together. This heterogeneity was consistent across time since the 1970s. Phylogenetic non-independence explained 66% of the variance in research effort (vs 58% for taxonomy). In addition, marine species, a group that only includes seven species, were particularly highly studied: five of the eight most studied Testudines are marine turtles. In contrast, freshwater and terrestrial species shared similar research effort values. Species occurring in Europe and North America were also more studied than species from, e.g., Central America or Africa, as were species that occur in the most scientifically active countries. In addition, species with a larger distribution range or introduced outside of their native range, as well as species with certain life history characteristics like larger clutches, smaller eggs or a longer longevity were more frequently investigated. In contrast, population trend and extinction risk did not predict research effort, although species that have not been assessed by the IUCN were characterized by a low research effort. This study underlines the importance of accounting for the heterogeneity in research effort across turtle species in global analyses and helps to identify taxa, regions, and life history strategies that should be the focus of more research.
Order of Authors:	Simon Ducatez
	Jayna L. DeVore
Response to Reviewers:	The key points that I would like to highlight are as follows:<br>1.Methodology: The study relies on the number of articles retrieved from the Web of Science. It is understood that a manual review of all relevant publications is impossible; however, semi-automated search strategies (WOS) may risk missing relevant data or including irrelevant ones. These issues should be more explicitly acknowledged. And discussed.<br>Our answer : We now discuss these issues more explicitly in both the methods and discussion (see lines 139 to 164 and 531 to 555). We have now developed this part of the discussion in more details to clearly underline the limitations of this index of research effort and suggest others that could give complementary information.<br>However, as mentioned in our responses to the reviewers’ comments, we believe that

this is a limited issue, as our objective was not to assess the absolute research effort, but to compare research effort across species. For example, we state when describing our methods: “Note that these indices are proxies for research effort, and not absolute estimates. They, for example, ignore the work that was published before 1975 as well as any reference that is not referenced in the WoS, including references from the grey literature. However, by constructing these indices, our purpose was to obtain a relative metric of research effort to compare species with each other, rather than an exact number of articles published per species. To test whether using different databases could affect this relative research effort score, we also compared the index obtained using the WoS database with the index extracted by Guedes et al. (2023) using Scopus and available for 343 of the 370 species considered.”

2.Geopolitical bias: Research effort likely reflects whether species occur in scientifically active countries (e.g., USA, Europe, and China). This should be analyzed more directly rather than only by broad regions.
Our answer: We now included an extra variable considering each species presence/absence in the 10 and 20 most scientifically active countries. It is indeed a significant predictor, though it did not affect our conclusions with regards to the other predictors, and it was not retained in our multi-predictors model.

3.Taxonomy update: Acknowledge the new 2025 Turtles of the World checklist (359 turtle and tortoise species now face extinction vs. 357 in 2021).
Our answer: We now updated the taxonomic list to the 2025 checklist, re-running all analyses after including the extra species. Note that this update was published in July 2025, after we submitted the first version of our manuscript.

When submitting your revision, we need you to address these additional requirements.
1. Please ensure that your manuscript meets PLOS ONE's style requirements, including those for file naming. The PLOS ONE style templates can be found at
https://journals.plos.org/plosone/s/file?id=wjVg/PLOSOne_formatting_sample_main_body.pdf and
https://journals.plos.org/plosone/s/file?id=ba62/PLOSOne_formatting_sample_title_authors_affiliations.pdf
2. Please note that PLOS One has specific guidelines on code sharing for submissions in which author-generated code underpins the findings in the manuscript. In these cases, we expect all author-generated code to be made available without restrictions upon publication of the work. Please review our guidelines at
https://journals.plos.org/plosone/s/materials-and-software-sharing#loc-sharing-code
and ensure that your code is shared in a way that follows best practice and facilitates reproducibility and reuse.
Our answer: We have added our code to the Suppl. Mat.

3. Thank you for stating the following financial disclosure:
“Agence nationale de la recherche, France InvEcoF #ANR-22-CE02-0007
Fondation Fyssen, France.”
Please state what role the funders took in the study. If the funders had no role, please state: "The funders had no role in study design, data collection and analysis, decision to publish, or preparation of the manuscript."
If this statement is not correct you must amend it as needed.
Please include this amended Role of Funder statement in your cover letter; we will change the online submission form on your behalf.
Our answer: Changed accordingly

4. Thank you for stating the following in the Acknowledgments Section of your manuscript:
“SD and JD were funded by the Agence nationale de la recherche, France InvEcoF #ANR-22-CE02-0007 and the Fondation Fyssen, France.”
We note that you have provided funding information that is currently declared in your Funding Statement. However, funding information should not appear in the Acknowledgments section or other areas of your manuscript. We will only publish funding information present in the Funding Statement section of the online submission form.
Please remove any funding-related text from the manuscript and let us know how you would like to update your Funding Statement. Currently, your Funding Statement reads

as follows:
“Agence nationale de la recherche, France InvEcoF #ANR-22-CE02-0007
Fondation Fyssen, France.”
Please include your amended statements within your cover letter; we will change the online submission form on your behalf.
Our answer: We have now removed the funding information from the acknowledgements section and included our funding statement and role of funder statement in the cover letter.

5. Please include captions for your Supporting Information files at the end of your manuscript, and update any in-text citations to match accordingly. Please see our Supporting Information guidelines for more information: http://journals.plos.org/plosone/s/supporting-information.
Our answer: We have now included the captions for Supporting Information at the end of our manuscript.

If the reviewer comments include a recommendation to cite specific previously published works, please review and evaluate these publications to determine whether they are relevant and should be cited. There is no requirement to cite these works unless the editor has indicated otherwise.

Additional Editor Comments:

Dear authors,

After carefully reviewing the manuscript and considering the feedback provided by the selected reviewers, I recommend that this paper can be considered for publication after the author (s) undertake major revisions.
For more information, please see the comments from reviewers.

Reviewers' comments:

Reviewer's Responses to Questions
Comments to the Author

Review Comments to the Author

Reviewer #1: This manuscript addresses a timely and important issue uneven research effort across Testudines species, highlighting biases and gaps that affect both ecological understanding and conservation actions. The study is ambitious in scale, data-rich, and well-documented, but several concerns need to be addressed before it can be considered for publication in PLOS ONE:
Our answer: We thank the reviewer for comments that helped improving the manuscript.

1. Justification of Methodological Choices (Search Strategy & Data Limitations)
The authors rely heavily on the number of articles retrieved from Web of Science using species names. While they attempt to validate the approach through correlation with Scopus data and synonym comparisons, this method can still under- or over-estimate research effort (e.g., name ambiguity, short genus names). Consider a more detailed justification or a sensitivity analysis beyond correlation coefficients.
Our answer: We further developed this important point in the discussion (see lines 535-547). Note that our index of research effort is not meant to provide an absolute, but a relative metric for research effort, as mentioned in the methods section. The objective is to compare species, not to know exactly how much research was devoted to each specie. As a result, the number of articles on large online databases such as Scopus and Web of Science have been largely used in previous work investigating research effort in other taxonomic groups. We already provide multiple sensitivity analyses, considering different search strategies in the search engines (title only, or more global search), multiple search engines (Scopus or Web of Science) and considering

Powered by Editorial Manager® and ProduXion Manager® from Aries Systems Corporation

synonyms or not. Although other sensitivity analyses could be considered, they would rely on extracting information from the grey literature or an in-depth analysis of each of the thousands of published articles that focus on turtles. We are not competent to automatize this type of analysis at this stage, and do not believe that it would bring much more information than confirming, with yet an extra sensitivity analysis, that all metrics for research effort are highly correlated. Note also that we are using an index that has been used by multiple authors in previously published work (including reptile work), and we have included more tests of the robustness of our index than most published work investigating taxonomic variation in research effort. This approach is a standard way to assess research effort and has been used by several papers assessing the heterogeneous study effort across species and its drivers (e.g., to illustrate how frequently these indices are used, here are a few examples of articles using similar indices, in addition to the articles already cited in our manuscript).

Adamo M, Chialva M, Calevo J et al (2021) Plant scientists' research attention is skewed towards colourful, conspicuous and broadly distributed flowers. Nat Plants 7:574–578.
Mammola S, Adamo M, Antić D et al (2023) Drivers of species knowledge across the tree of life. eLife 12:RP88251
Simoncini, A., Ficetola, G.F. & Lattanzi, M. Taxonomic bias towards charismatic and easy-to-find mammals shapes knowledge of parasites. Biodivers Conserv (2025). https://doi.org/10.1007/s10531-025-03147-1
Tam J, Lagisz M, Cornwell W et al (2022) Quantifying research interests in 7,521 mammalian species with h-index: a case study. Gigascience 11:giac074.
Wilson JRU, Proches S, Braschler B et al (2007) The (bio)diversity of science reflects the interests of society. Front Ecol Environ 5:409–414. https://doi.org/10.1890/060077.1

2. Treatment of Sea Turtles as Outliers
While the rationale to exclude sea turtles from the multivariate analysis is understandable due to their high representation, this introduces inconsistency: the main claim about marine species dominating research becomes methodologically decoupled from the model-based inference. Consider conducting parallel analyses (with and without sea turtles) to better understand their effect.
Our answer: We agree with the reviewer that parallel analyses including vs excluding sea turtles for the global model would be preferable. We now ran the multivariate analysis twice, either with or without sea turtles. We show the results of the model on all species in the main text, and the results of the model excluding sea turtles in the Supplementary Material. Note that the results were consistent between these two datasets.

3. Insufficient Integration of Conservation Implications
The Discussion does mention that endangered species are not more studied, but the conservation implications remain superficial. The authors should provide clearer suggestions: How can their findings be translated into priority research planning, especially for data-deficient taxa?
Our answer: We added more discussion on this aspect lines 524-530. Note that our analyses mostly identify a need for more work to clearly identify how these research gaps can affect our ability to design conservation plans for endangered species, as discussed in the paragraph lines 507-530. Specifically, our findings underline that species that are at a higher risk of extinction, though they should be prioritized by researchers to favor their conservation and/or recovery, are not more often studied than less threatened species. We believe that our index or research effort can be used as an extra argument to help obtaining funding for under researched species (either data deficient or unassessed ones) and endangered ones.

4. Trait–Effort Relationships Need Clarification
The final multivariate model includes biologically meaningful traits like body size, longevity, and clutch size. However, clutch size switches from a positive association in the univariate to negative in the multivariate model, without adequate explanation. Please address potential collinearity or scaling issues, and interpret biological significance more clearly.
Our answer: We agree with the reviewer that this switch is unclear and could be due to

collinearity issues, or differences in the set of species considered in the monovariate and multivariate models. The correlation between max SCL and clutch size was of 0.568 – to be safe and avoid any risk of collinearity, we therefore decided to exclude clutch size from the multivariate model.
Note that to better understand the switch in clutch size effect (even though we are not presenting this switch in the MS anymore), we built a model with clutch size as the only predictor considering the species that were included in the multivariate model only, to determine whether the switch could be due to a change in the set of species considered. This resulted in a non-significant effect of clutch size, supporting the idea that both collinearity between clutch size and SCL, and the subsample of species on which the multivariate analysis could be conducted, explain the discrepancy between the univariate and multivariate models with regard to the clutch size effect.

5. Geographic Bias Discussion Needs Expansion
The paper identifies geographic disparities (e.g., North America vs. Africa), but lacks explanation of underlying drivers (e.g., funding, infrastructure, language bias). Incorporate literature from bibliometric studies or conservation research bias.
Our answer: We have now added an extra analysis as suggested by reviewer 2, comparing species present in the 10 or 20 most scientifically active countries with species that are absent from these countries. We also added sentences of discussion on this aspect lines 459-461. This was also discussed in the paragraph lines 499-506.

Suggested Additional Analyses (if feasible):
1- A network map of author affiliations vs. species studied might add depth to understanding geographic biases.
Our answer: Although we agree that this would be an interesting addition, this was out of the scope of the manuscript and would require data extraction from each of the thousands of articles on the different turtle species. All of these data extractions were conducted by hand, so this would add a massive amount of work. A future study using AI might bring this information more easily.

2- A comparison between publication count and conservation funding per species or region, if data are available.
Our answer: We also agree that this would be a welcome future study, though this was out of the scope of our study. We thank the reviewer for this suggestion which we added as a perspective in the discussion (see lines 528-530).

Conclusion:
This manuscript has the potential to make a meaningful contribution to reptile conservation and bibliometric ecology, but it requires significant revisions to improve its methodological rigor, analytical clarity, and conservation relevance.
Please revise the manuscript accordingly and provide a detailed point-by-point rebuttal.
Our answer: We are grateful to the reviewer for constructive comments that helped us improving the quality of our study.

Reviewer #2: This is an interesting contribution with implications not only for the turtles, but for the approach to biodiversity studies as a whole. I need to stress that I am not an expert in statistics, so those should be checked by a specialist in the field. However, I can provide some insights from the general biological and herpetological point of view. Overall, I think the paper is well-written and it should be published but I see some issues, mostly with how the results are presented, as I think that the potential of the data is not fully utilized and some aspects may require slight refinement or a better justification.
Our answer: We thank the reviewer for comments that helped improving the manuscript.

The topic is somewhat difficult from the methodological standpoint because, given the vast number of published papers, it is not really possible to review their contents manually, whereas any semiautomatic approach (such as the one employed by the Authors) will unavoidably result in some errors. For example, some papers providing valuable information on some species may be overlooked, because they do not include the full name of the species in any of the fields searchable by the Web of Science, but instead use, e.g., only the generic name or the name of the family (as I understand, the Authors did not look for those, only for the binomina). On the other hand, in some

cases a specific name may be used in an abstract, e.g., solely in a comparative context without any meaningful information on that species presented in the paper (false hits), or as a synonym (double hits when both variants of a name are searched for). I understand that these instances are inevitable and while some workarounds can be proposed, they cannot be fully eliminated. I also understand that the cutoff used by the Authors (1975) is a sad necessity due to the increasingly limited availability of data for processing (that could be, to some extent, helped by inclusion of, e.g., the Biodiversity Heritage Library) but also due to complications regarding the synonymy and more frequent usage of sole generic names in those older papers. Unfortunately, this leaves out some important anatomical and ecological literature, also regarding the less popular taxa, which is still useful and informative and which in some cases may render restudy of those species in modern times less of a priority, thus causing them to appear even less studied in the selected timeframe. This should be perhaps acknowledged slightly more in the text (although the Authors already referred to that problem) but other than that we cannot do much about it and I accept that as an inherent limitation of that kind of research.
Our answer: As mentioned in our response to the editor and reviewer 1, we developed the discussion on this issue, although we already discussed it in both the methods and discussion of the previous version of the MS (see lines 139 to 164 and 531 to 555). As mentioned in the manuscript as well as in response to reviewer 1, the objective of this research index is not to provide an absolute metric for species research effort, but an index that allows to compare species. There is a lot more literature on turtles that is not included in WOS or Scopus, and conversely, some articles that came up in our search did not necessarily focus on the species of interest (even if the species name is given in the abstract, it does not systematically imply that the article is focused on the species of interest). Our purpose was however not to produce an exact and exhaustive list of articles per species, but rather to compare the relative amount of research that has been devoted to each species. Although pre-1975 research can be very important and should not be neglected by researchers working on particular species, the amount of research articles that have been produced since 1975 is overwhelmingly larger, by multiple orders of magnitude. This increase in the quantity of research conducted since the 1970 has been described elsewhere in details (see also our response to another comment of reviewer 2). So even if it is possible that some species that were under studied since 1975 were the focus of several studies before that date, it would likely only slightly alter their score, and be of very little effect on the global trends that we aimed at testing here. We have now added more discussion on this aspect lines 535-547.

One oversight and potential problem I see with communicating the methodology is related to Fig. 1. It presents the number of review papers on turtles. Unfortunately, I don't see any notion of such a search in the Methods section and the methodology for compiling that charts seems to be only explained in the figure caption. However, this does not seem right for two reasons. (1) using solely the given query ("tortoise*" OR "chelonian*" OR "turtle*" OR "testudines*") one is expected to find not only papers relevant to the reviewed manuscript, but potentially also papers completely out of scope, e.g., on extinct stem turtles, turtle-derived materials (tortoiseshell), arthropods (tortoise beetles), etc., so the result does not really say much. (2) I tried to see how badly out of scope the results are in that case, but I could not replicate the Author's results. I searched for the provided keywords, string copied directly from the figure caption, and document type set to Review Article. I will give the values for the year 2022, because this is the largest number in the Author's search and shows the problem the most easily, but there were also differences for other years. When searching in Author Keywords, 21 articles were found. This is much below the over 25 papers found by the Authors for that year. When searching in Keywords plus, the search yielded 58 papers for that year – this is much more. Also, as expected, the search found lots of papers on fossil turtles and some nonsensical ones (e.g., "Plant phenolics: neglected secondary metabolites in plant stress tolerance"). Please check and preferably refine – the Author Keywords yielded more sensible results in that case and about 250 papers total, so it is doable to leave out those that are out of scope). In case the Authors searched in Keywords Plus and manually rejected results out of scope (possible but I think unlikely, as that search produced over 700 results for me) then kudos to them but please communicate that in the Methods!
Our answer: We have now removed this figure, which was only meant to provide a simple illustration on the increase in reviews and large taxonomic scale comparative

studies on the Testudinae group to further illustrate the need for our study, in the introduction. This is why we did not mention this approach in the methods, but instead directly in the introduction. Our purpose was not to be exhaustive, but to show the relative increase in these large-scale studies that sometimes rely on the expectation that all species are equally known, or that aim at providing patterns or mechanisms that are true at the scale of the entire clade. Why the reviewer did not find the same numbers as us is unclear to us, as we reran these searches and found very similar numbers for each year. Note however that the WoS database is frequently changed, with new references added or removed regularly, which may explain this discrepancy (though to our experience, the differences are usually minor). We removed the figure to avoid any misunderstanding with regards to the interpretation of the figure, and because it was not absolutely needed.

The most important aspect, which is currently very indirectly and tangentially tackled by the Authors but certainly requires more attention as a potential good predictor of research effort, is simply whether the species in question is native to territories of more scientifically active countries (e.g., with better financing of scientific institutions). This may very well explain why some taxa became sort of model species for turtles and why they continue to gain attention while other remain neglected. Given the huge differences in research effort between species, some receiving next to no attention and some literally thousands of papers, I suspect that such geopolitical factors may not only be significant, but even overpower other traits – especially that, if I see correctly, all but one species with over 150 papers on their account occur either in the USA, Western or Southern Europe, or China, whereas none of at least several dozens of the least studied species (except of some artificially underestimated instances – see below) occurs there. Currently, the Authors only briefly consider that topic in terms of generalized geographic regions (North America, Central America, South America, etc.) but I am afraid this misses a lot of nuance, especially that a number of taxa falls into the uninformative “Multiple” category. Analyzing whether there is a statistically significant correlation between the research interest and the overlap of the species range with the territory of one or more of, let’s say, the 10 or 20 most scientifically active countries may prove extremally interesting and should not require a lot of additional work and may explain why some taxa inhabiting the same large-scale geographic area differ significantly in attention. I propose that the Authors check and discuss whether this is indeed the case.
Our answer: We followed the reviewer’s advice and compared the research effort of the Testudinae species occurring the 10 or 20 most scientifically active species, using a research ranking score per country, and ranking each species according to whether or not it occurs within the top 10 or top 20 countries. As predicted by the reviewers, there is a strong discrepancy, with species occurring in the most scientifically active countries being also the focus of more research. We now include this variable in both a univariate and multivariate models, though it was not a significant predictor in the multivariate model (likely because it is redundant with the geographic region that was already included). A diversity of socio-economic predictors are likely to affect research effort, including factors related to the cultural and economic importance of some taxa; other works have focused on these types of predictors to understand their role (though to the best of our knowledge, this has not been investigated in turtles). In this study, however, our purpose was not to test these questions in depth, nor to disentangle all drivers of variation in research effort. Instead, we were mostly interested in underlining the existence of research-biases across Testudinae species, and of their potential consequences for the interpretation of large scale comparative analyses. Elucidating all the causes of research-biases was out of the scope of our study.

Additionally, the Authors should address the problem of recently established species, such as Kinosternon steindachneri (2013) and Sternotherus intermedius (2017), which did not yet accumulate a significant bibliography of their own, but were considered historically as part (subspecies or subpopulation) of their closely related species (in the case of Kinosternon steindachneri – Kinosternon subrubum) or hybrids (in the case of Sternotherus intermedius). As a result, true research effort devoted to them is either difficult to establish or artificially underestimated, in turn possibly skewing indices for corresponding supraspecific taxa and traits. If possible, in those instances it would be advisable to track the research effort on the subspecies rather than species level. Otherwise, they should probably be either considered jointly with their sister species or

Powered by Editorial Manager® and ProduXion Manager® from Aries Systems Corporation

left out, unless their impact is confirmed to be insignificant.
Our answer: We agree that a limitation of our index of research effort is that it does not give the “true” research effort value for each species. It was however not our purpose, as we aimed at obtaining a relative index comparable across species (see our answer to previous comments). In the case of recently established species, if they were recently split and previously considered as a subspecies of other species, the subspecies name was included in the list of synonyms, and therefore considered (based on synonymy) even if the species was only recently recognized as a full species. Note that tracking subspecies research effort is particularly challenging as most studies do not precise which subspecies is investigated, but only provide the species name.

Another thing that may deserve reconsideration on the part of the Authors is the usage of full decades in some of their analyses, despite their timeframe starting and ending in the middle of the decades (1975–2024). Five-year bins may perhaps be more adequate?
Our answer: Our purpose here was to test for changes in research effort discrepancy over time. Since the split into decades was sufficient to test and detect this effect, we decided not to include the five-years steps which would require to rerun every search species by species for each 5-year period, with no clear extra information for our understanding of variation in research effort across time.

Finally, the paper is mostly built on the taxonomical framework established by the 2021 ninth edition of the Turtles of the World checklist. However, a new edition (tenth, 2025) is already available, so the manuscript should be updated to acknowledge that. The new edition distinguishes 359 extant species instead of 357 recognized by TotW 2021. Whether those new species should be considered on their own depends on the approach (see above), but a lot of attention has been given to turtle extinctions in the new volume, so it is relevant at least in that context.
Our answer: We have now updated our dataset with this 2025 edition of the checklist and reran all analyses considering this new framework (we also updated the historic range size values). Note that this new checklist was published on July 25th, 2025 – so after we’d submitted the previous version of the manuscript. Note that we also updated the scores of extinction risk and population trend for all species using the newly published IUCN red list (i.e., the 2025-2 version, from October 2025).

More detailed notes are provided below:
P. 3, lines 43–44 („Testudines emerged during the Triassic and survived two mass extinction crises that eliminated several other groups of reptiles at the end of the Permian and Cretaceous”): This statement is incorrect for two reasons. First, the Testudines are the crown group composed from two extant clades: the side-necked turtles (Pleurodira) and hidden-necked turtles (Cryptodira). The exact time of divergence or those two clades (and, in consequence, the appearance of the Testudines) is somewhat contentious, as it depends on the classification of some poorly known fossil taxa, but it was certainly later than in the Triassic, most probably in the Middle to Late Jurassic. All Triassic turtles are stem taxa so they belong to Testudinata, not Testudines. Second, the Permian was before the Triassic, so neither the Testudines nor the Testudinata (as a group appearing at the end of the Triassic) could not survive the Permian extinction. I imagine that the Authors may confuse here the Testudines, Testudinata, and Pan-Testudines/Pantestudinata (a larger group including also shell-less turtle relatives, possibly also the Permian Eunotosaurus africanus), but these not the same and the paper focuses solely on Testudines proper, so there is no need to bring those earlier forms up.
Our answer: We thank the reviewer for this important point, apologize for our mistake and removed this sentence from the introduction.

P. 3, lines 48 and 87 („Squamates”, “Amphibians”): when anglicized, the name should not be capitalized (so „Squamata” and “Amphibia” would be correct but in that case „squamates” and “amphibians”).
Our answer: Changed accordingly.

P. 4, lines 104–105 (“fast lifestyle”, “slow lifestyle”): this seem somewhat poorly fitting terms, maybe “life cycle”?
Our answer: We changed these into fast and slow life history strategies (see lines 103-

Powered by Editorial Manager® and ProduXion Manager® from Aries Systems Corporation

104).

P. 6, line 186 (“maximum standard carapace length”): I am unfamiliar with such a measurement and I think the Authors mean here the “maximum straight-line carapace length” (a common measurement used for turtles and typically abbreviated as SCL, also used by Rhodin et al., 2021). If so, please also correct elsewhere. If not, please define.
Our answer: The reviewer is right and we changed this (into “straight-line carapace length”) throughout the manuscript.

P. 8, line 313: can this increase be partially explained by the incomplete sampling of older articles in the WoS, e.g., published in historical but now discontinued journals which are not indexed?
Our answer: page 8 line 313 of the previous version of the MS refers to results showing the proportion of variance in research effort explained by taxonomy. This comment is likely referring to the previous paragraph and page, which shows the increase in research effort across time (lines 305-309)? If so, the increase in the number of publications across time is known and well documented globally (not only in biology) and was expected for Testudines research as well. We decided not to discuss this question in our manuscript is this was out of the scope of our study, and it has been the focus of diverse studies. Note that discontinued journals are also included in the WoS or Scopus databases. A few examples of work showing and discussing the causes of this global increase:
https://direct.mit.edu/qss/article/5/4/823/124269/The-strain-on-scientific-publishing
Thekwall and Sud 2022 Scopus 1900–2020: Growth in articles, abstracts, countries, fields, and journals. Quantitative Science Studies (2022) 3 (1): 37–50.
Fire and Guestrin 2019 Over-optimization of academic publishing metrics: observing Goodhart's Law in action. GigaScience, Volume 8, Issue 6, giz053

P. 10, line 401 (“Trachemys scrypta”): Trachemys scripta.
Our answer: Changed accordingly.

Table 1: I think I understand what the Authors want to show here, but at the same time there is something missing. The juxtaposition of the taxonomic richness of the family and the number of papers is some information, but this does not say too much without some measure of the evenness of coverage. As presented, the Chelydridae are in the third spot with a decent 243 papers per species, which looks very good, but what of it, if nearly 90% of that (!) is devoted to one species. Conversely, the Geoemydidae only have 16 papers per species, so they appear significantly understudied compared to the chelydrids, but in reality the research effort is much more evenly distributed. In consequence, I feel the message here falls into the same pitfall which the Authors try to solve in their manuscript – we see an averaged picture but completely miss how some species are understudied. Also, the numbers alone are difficult to visualize, so the nice data that the Authors gathered get somewhat lost. I would strongly suggest presenting this in some more visual way. Perhaps a bar chart with the bars subdivided into sections corresponding to the proportional research attention received by individual species, or a pie chart with subdivided slices? I get it that the number of species within many families is too large to show all of them within a single bar/slice, but the differences in attention between them are large enough that the best studied species within each family should be visible as individual portions, whereas the least studied species may be grouped together. This would not only nicely show the differences between the families, but it would also present the disproportions in the effort within each of them and it would also be much more visually appealing.
Our answer: We thank the reviewer for this suggestion. We have now added to this table the standard error for each family as an index of research effort evenness across species within families. We also followed the reviewer advice by adding a new figure with a boxplot showing the distribution of research effort within each family, with the four quartiles, mean, median and number of species per family (see Figure 2).

Fig. 1 caption: “Evolution” is not a good term here, I would suggest just leaving “The number of review articles” etc.
Our answer: We have now removed this figure from the manuscript.

Figs 4 and 5: Please explain what do “a”, “b”, and “c” mean.

	Our answer: We now provide this information in both captions.<br>We are very grateful to Tomasz Szczygielski for constructive comments that helped us to improve our manuscript.
**Additional Information:**	
**Question**	**Response**
**Financial Disclosure**<br><br>Enter a financial disclosure statement that describes the sources of funding for the work included in this submission. Review the submission guidelines for detailed requirements. View published research articles from *PLOS ONE* for specific examples.<br><br>This statement is required for submission and **will appear in the published article** if the submission is accepted. Please make sure it is accurate.<br><br>**Funded studies**<br>Enter a statement with the following details:<br>• Initials of the authors who received each award<br>• Grant numbers awarded to each author<br>• The full name of each funder<br>• URL of each funder website<br>• Did the sponsors or funders play any role in the study design, data collection and analysis, decision to publish, or preparation of the manuscript?<br><br>Did you receive funding for this work?	Yes
Please add funding details.<br>as follow-up to "**Financial Disclosure**<br><br>Enter a financial disclosure statement that describes the sources of funding for the work included in this submission. Review the submission guidelines for detailed requirements. View published research articles from *PLOS ONE* for specific examples.<br><br>This statement is required for submission and **will appear in the published article** if the submission is accepted. Please make	Agence nationale de la recherche, France InvEcoF #ANR-22-CE02-0007<br><br>Fondation Fyssen, France

sure it is accurate.<br><br>**Funded studies**<br>Enter a statement with the following details:<br>• Initials of the authors who received each award<br>• Grant numbers awarded to each author<br>• The full name of each funder<br>• URL of each funder website<br>• Did the sponsors or funders play any role in the study design, data collection and analysis, decision to publish, or preparation of the manuscript?<br><br>Did you receive funding for this work?"	
Please select the country of your main research funder (please select carefully as in some cases this is used in fee calculation).<br>as follow-up to "**Financial Disclosure**<br><br>Enter a financial disclosure statement that describes the sources of funding for the work included in this submission. Review the submission guidelines for detailed requirements. View published research articles from *PLOS ONE* for specific examples.<br><br>This statement is required for submission and **will appear in the published article** if the submission is accepted. Please make sure it is accurate.<br><br>**Funded studies**<br>Enter a statement with the following details:<br>• Initials of the authors who received each award<br>• Grant numbers awarded to each author<br>• The full name of each funder<br>• URL of each funder website<br>• Did the sponsors or funders play any role in the study design, data collection and analysis, decision to publish, or preparation of the manuscript?<br><br>Did you receive funding for this work?"	FRANCE - FR

**Competing Interests**<br><br>Use the instructions below to enter a competing interest statement for this submission. On behalf of all authors, disclose any competing interests that could be perceived to bias this work—acknowledging all financial support and any other relevant financial or non-financial competing interests.<br><br>This statement is required for submission and **will appear in the published article** if the submission is accepted. Please make sure it is accurate and that any funding sources listed in your Funding Information later in the submission form are also declared in your Financial Disclosure statement.<br><br>View published research articles from *PLOS ONE* for specific examples.<br><br>**NO authors have competing interests**<br><br>Enter: *The authors have declared that no competing interests exist.*<br><br>**Authors with competing interests**<br><br>Enter competing interest details beginning with this statement:<br><br>*I have read the journal's policy and the authors of this manuscript have the following competing interests: [insert competing interests here]*<br><br>* typeset	The authors have declared that no competing interests exist.
**Ethics Statement**<br><br>Enter an ethics statement for this submission. This statement is required if the study involved:<br><br>• Human participants<br>• Human specimens or tissue<br>• Vertebrate animals or cephalopods	N/A

Powered by Editorial Manager® and ProduXion Manager® from Aries Systems Corporation

• Vertebrate embryos or tissues<br>• Field research<br><br>Write "N/A" if the submission does not require an ethics statement.<br><br>General guidance is provided below. Consult the submission guidelines for detailed instructions. **Make sure that all information entered here is included in the Methods section of the manuscript.**	

**Format for specific study types**<br><br>**Human Subject Research (involving human participants and/or tissue)**<br>• Give the name of the institutional review board or ethics committee that approved the study<br>• Include the approval number and/or a statement indicating approval of this research<br>• Indicate the form of consent obtained (written/oral) or the reason that consent was not obtained (e.g. the data were analyzed anonymously)<br><br>**Animal Research (involving vertebrate animals, embryos or tissues)**<br>• Provide the name of the Institutional Animal Care and Use Committee (IACUC) or other relevant ethics board that reviewed the study protocol, and indicate whether they approved this research or granted a formal waiver of ethical approval<br>• Include an approval number if one was obtained<br>• If the study involved *non-human primates*, add *additional details* about animal welfare and steps taken to ameliorate suffering<br>• If anesthesia, euthanasia, or any kind of animal sacrifice is part of the study, include briefly which substances and/or methods were applied<br><br>**Field Research**<br><br>Include the following details if this study involves the collection of plant, animal, or other materials from a natural setting:<br>• Field permit number<br>• Name of the institution or relevant body that granted permission	
**Data Availability**<br><br>Authors are required to make all data underlying the findings described fully available, without restriction, and from the time of publication. PLOS allows rare exceptions to address legal and ethical concerns. See the PLOS Data Policy and FAQ for detailed information.	Yes - all data are fully available without restriction

Powered by Editorial Manager® and ProduXion Manager® from Aries Systems Corporation

A Data Availability Statement describing where the data can be found is required at submission. Your answers to this question constitute the Data Availability Statement and **will be published in the article**, if accepted.<br><br>**Important:** Stating 'data available on request from the author' is not sufficient. If your data are only available upon request, select 'No' for the first question and explain your exceptional situation in the text box.<br><br>Do the authors confirm that all data underlying the findings described in their manuscript are fully available without restriction?	
**Describe where the data may be found in full sentences. If you are copying our sample text, replace any instances of XXX with the appropriate details.**<br><br>• If the data are **held or will be held in a public repository**, include URLs, accession numbers or DOIs. If this information will only be available after acceptance, indicate this by ticking the box below. For example: *All XXX files are available from the XXX database (accession number(s) XXX, XXX.*).<br>• If the data are all contained **within the manuscript and/or Supporting Information files**, enter the following: *All relevant data are within the manuscript and its Supporting Information files.*<br>• If neither of these applies but you are able to provide **details of access elsewhere**, with or without limitations, please do so. For example:<br><br>*Data cannot be shared publicly because of [XXX]. Data are available from the XXX Institutional Data Access / Ethics Committee (contact via XXX) for researchers who meet the criteria for access to confidential data.*<br><br>*The data underlying the results presented in the study are available from (include the name of the third party*	All relevant data are within the manuscript and its Supporting Information files

and contact information or URL).<br>• This text is appropriate if the data are owned by a third party and authors do not have permission to share the data.<br><br>* typeset	
Additional data availability information:	

Powered by Editorial Manager® and ProduXion Manager® from Aries Systems Corporation

Manuscript
Click here to access/download;Manuscript;Research effort turtles revised.docx

Which turtle should I study? Uneven distribution of research effort across Testudines species

Authors: Simon Ducatez & Jayna Lynn DeVore

Simon Ducatez
Affiliation: UMR 241 SECOPOL (IRD, IFREMER, ILM, UPF)

Jayna L. DeVore
Affiliation: UMR 241 SECOPOL (UPF, IRD, IFREMER, ILM)

Corresponding author: Simon Ducatez, simon.ducatez@gmail.com

Abstract

Understanding why some species are more often studied than others can provide valuable information on potential biases that research effort discrepancies can cause and help to direct future research to mitigate these biases. Turtles form an order of reptiles in which the majority of species (67%) are threatened with extinction, but they show high variation in the amount of research devoted to each species. We aimed to quantify this variation across all recognized extant species, and to decipher how this distribution of ~~this~~ research effort varied according to species taxonomy, phylogeny, distribution, ecology, life history and extinction risk. Using the number of references listed on the Web of Science for each species as a proxy for species research effort, we first show that this index is both robust to variation in search methods within WoS, and consistent with an index extracted from another online library (Scopus). The number of articles per species varied substantially, with 3441 articles for the green sea turtle alone, but only 475 total articles for all 50% least studied species taken together. This heterogeneity was consistent across time since the 1970s. Phylogenetic non-independence explained 66% of the variance in research effort (vs 58% for taxonomy). In addition, marine species, a group that only includes seven species, were particularly highly studied: five of the eight most studied Testudines are marine turtles. In contrast, freshwater and terrestrial species shared similar research effort values. Species occurring in Europe and North America were also more studied than species from, e.g., Central America or Africa, as were species that occur in the most scientifically active countries. In addition, species with a larger distribution range or introduced outside of their native range, as well as species with certain life history characteristics like larger clutches, smaller eggs or a longer longevity were more frequently investigated. In contrast, population trend and extinction risk did not predict research effort, although species that have not been assessed by the IUCN were characterized by a low research effort. This study underlines the importance of accounting for the heterogeneity in research effort across turtle species in global analyses and helps to identify taxa, regions, and life history strategies that should be the focus of more research.

Keywords: Chelonian, research bias, tortoise, turtle

Introduction

Particularly sensitive to the current extinction crisis (Lovich *et al.* 2018; Stanford *et al.* 2020), Testudines are one of the most threatened groups of vertebrates : 67% of the assessed extant species are threatened with extinction (IUCN 2025), as compared to 25% of mammals, 20% of squamates and 14% of birds. Although several threats are responsible for this pattern, wildlife trafficking (for pet trade, turtle meat or body parts) was identified as the most damaging threat driving many species towards extinction (Stanford *et al.* 2020). Often referred to as the "Asian turtle crisis" because of how important this threat is in Asia (Cheung & Dudgeon 2006; Chen *et al.* 2009), it has recently been named a "Global turtle crisis" (Seminoff 2025) given the universal nature of turtle trafficking (e.g., see Shearer & Türkozan 2025). Testudines occupy terrestrial, freshwater and marine ecosystems, where they participate in a diversity of ecosystem functions, including herbivory, seed dispersal and detritivory (Froyd *et al.* 2014; Falcón & Hansen 2018; Falcón *et al.* 2020; Christianen *et al.* 2023; Tapia Aguilera & Gibbs 2023). Their functional role in some ecosystems is sufficiently important that tortoises have become a model group in ecosystem restoration via rewilding, including through taxon substitution (Hansen *et al.* 2010; Griffiths *et al.* 2011; Svenning *et al.* 2016; Falcón & Hansen 2018; Tapia *et al.* 2022; Jones *et al.* 2023; Tapia Aguilera & Gibbs 2023). Understanding the ability of Testudines to respond to current environmental changes is therefore important to better preserve not only the turtles and tortoises themselves but also the functioning of the ecosystems they are part of.

Although some particularly iconic species draw a lot of public interest and have attracted an important amount of research (e.g., marine species), many species remain poorly known. This knowledge gap is likely to impact our ability to globally understand the ecological roles of turtles and tortoises, the capacity of this unique taxonomic group to respond to current biodiversity and climate crises, and our ability to preserve this group. Thanks to the efforts of multiple researchers, and especially of the Turtle Taxonomy Working Group (Rhodin *et al.* 2021; Rhodin *et al.* 2025), the current knowledge of Testudines species has been assembled, and the risk of extinction of 70% of the 359 currently recognized extant turtle species has now been assessed (IUCN 2025). However, the conservation status of the remaining 30% (110 species) remains unclear. This reflects the existence of important discrepancies in the amount of research that has been dedicated to the different species of Testudines.

Knowledge gaps and discrepancies in research effort can affect our understanding of the ecology, evolution, and conservation of Testudines for several reasons. First, they lead to the exclusion of the least studied species from reviews and comparative analyses, making the conclusions of these global studies less representative and more difficult to interpret. Second, they can affect trait estimates, e.g., because more extreme values are likely to be recorded in more investigated species. Third, they can alter the accuracy of species trait value estimates. Such values may be biased towards one or few individuals or populations in understudied species, but more representative of the entire species in more studied species. Finally, these knowledge gaps directly affect researchers and experts' ability to assess the conservation status of understudied species, excluding them *de facto* from conservation programs.

Such research gaps are common across taxonomic groups and have been identified across a diversity of taxa. Predictors of taxonomic variation in research effort have for example been investigated in birds (Ducatez & Lefebvre 2014), mammals (Santos *et al.* 2020; de Figueiredo Machado *et al.* 2023), fishes (Ducatez 2019; Guy *et al.* 2021), amphibians (Silva *et al.* 2020) and plants (Adamo *et al.* 2021). Recently, a global assessment of variation in research effort across reptile species was conducted (Guedes *et al.* 2023), revealing important variation across reptile groups, and

identifying biological and socioeconomic factors that determine how often a given species is studied. Such broad scale comparative analyses are particularly useful to identify factors determining discrepancies in research effort across species, and help mitigating these. Guedes *et al.* (2023) also brought to light an important fact about Testudines: although this class emerges as one of the most studied on average among reptiles, it also shows extreme variation within the group, with families such as the Cheloniidae having attracted the most attention while others such as Chelydridae were much less studied (Guedes *et al.* 2023). These observations call for a more detailed investigation of variation in research effort across Testudines species, an aspect that was not investigated in this previous work.

The objective of this study is to provide such investigation. On the one side, we aimed to identify which species and families are particularly studied, and which ones are particularly neglected, and at deciphering whether this relates to the ecology, life history and distribution of these species. For example, are aquatic turtles more often studied than terrestrial tortoises? Are species with a fast life history (early reproduction and shorter lifespan) more studied than species with a slow life history (late age at first reproduction, high longevity)? How does research effort vary across biogeographic realms in Testudines? Is research effort targeting species that have a higher risk of extinction? By clearly identifying understudied species, we hope to motivate the funding of research programs aiming at filling these knowledge gaps. In parallel, we aimed to determine how research effort variation may affect our global understanding of the ecology, evolution and conservation of turtles and tortoises. Literature reviews are necessary to understand global patterns and mechanisms within entire taxonomic groups such as the Testudines. However, they are also limited by the available literature and data: if research is taxonomically biased, the conclusions drawn from these presumably global reviews are likely to also be taxon-specific, making it important to identify these biases. Differences in research effort may also have direct consequences for some trait estimates. For example, turtles and tortoises are famous for their long life expectancy, with records in some species exceeding human lifetimes (> 100 years in several giant tortoise species, Bonin *et al.* 2006). Record longevities are however more likely to be detected in intensively studied species, as compared to less studied ones. Progress in our knowledge of Testudines phylogeny (there is now a tree based on genetic data from 80% of all turtle species: Thomson *et al.* 2021), together with advances in statistical analyses now allow researchers to run large scale analyses considering most species in this order and to bring a more global understanding of the importance of this group, but also of the threats it is facing. Identifying knowledge gaps should help taking them into account when designing these global analyses and interpreting their results.

Here, we first provide an index of research effort for each extant Testudines species, available for use in comparative studies. We tested for its robustness by comparing it with other indices. Second, we investigated how spread out this research effort is, and tested whether research discrepancies improved or, on the contrary, became more extreme across time. Third, we investigated whether variation in research effort was related to taxonomy and phylogeny, or to 14 variables describing the distribution range, ecology, and life history of turtles. Finally, we tested whether research effort was predicted by extinction risk and population trend.

Methods

Research effort estimation

We used the taxonomy from the Turtle Taxonomy Working Group 2025 (Rhodin *et al.* 2025), except for the Galapagos tortoises (*Chelonoidis sp.*) for which we followed the taxonomy

considered by the IUCN, which recognizes 12 species that are considered as 12 subspecies of *C. niger* by Rhodin *et al.* (2025). This allowed to include this important taxon in the analyses considering extinction risk (the IUCN provides 12 different assessments for this set of 12 taxa) and resulted in a total of 370 extant species.

We estimated the research effort devoted to each one of these 370 species by extracting the number of references listed in the Web of Science (WoS) database (extraction conducted in January 2024). For each species, we conducted a search on WoS for papers published between 1975 and 2023, using the current species scientific name (e.g., "*Gopherus morafkai*") as keyword, and considering *all fields* (not just the title or keywords). We then extracted the number of articles obtained in the search as the index of research effort. In parallel, we conducted the same search but considering all synonyms of each species as keywords using the synonyms listed in Rhodin *et al.* (2025) (e.g., "*Gopherus morafkai*" OR "*Xerobates morafkai*"), to determine how name changes affected the research effort estimates. Progress in taxonomy can lead to multiple changes in species names, so that only including the current name may underestimate the research effort for some species (Correia *et al.* 2018). We also ran these two searches again but focusing on titles only, to compare an index of research effort based on the number of papers that are most likely to be focused on the focal species (as its name is in the title) with a more inclusive index that also considers articles including the focal species name in the abstract or keywords. Even in an article focused on a given species, the name of the species may not appear in its title, but in the keywords or abstract. In contrast, a species name may appear in the abstract or keywords of an article even if the article is not focused on that species. Comparing the research effort considering titles only vs all fields should allow us to determine whether changing the search method affects the resulting indices. Note that these indices are proxies for research effort, and not absolute estimates. They, for example, ignore the work that was published before 1975 as well as any reference that is not referenced in the WoS, including references from the grey literature. However, by constructing these indices, our purpose was to obtain a relative metric of research effort to compare species with each other, rather than an exact number of articles published per species. To test whether using different databases could affect this relative research effort score, we also compared the index obtained using the WoS database with the index extracted by Guedes *et al.* (2023) using Scopus and available for 343 of the 370 species considered.

Variation in research effort distribution across time

To assess variation in research effort across time and species, we extracted the number of papers referenced in the WoS database for each species and decade (i.e., from 1980 to 1989, from 1990 to 1999, from 2000 to 2009, and from 2010 to 2019). For this approach, we included all fields (not just the title) and considered all synonyms of a given species name as keywords to be more inclusive and to account for name changes across time.

Trait collection

We extracted values for 14 variables likely to either affect research effort or be affected by research effort: biogeographic realm, country research rank, range size, insularity, habitat, successful introduction, diet, carapace length, maximum longevity, generation length, female age at maturity, clutch size, incubation time and egg length. We extracted information for each species on biogeographic realm (ten different regions and one category for species occupying two or more

regions), range size (both the estimated indigenous (historical) range (AOO) from Rhodin *et al.* (2025)and current range size available in Guedes *et al.* (2023)), habitat (marine, terrestrial, freshwater or mixed for species occurring in both terrestrial and freshwater habitats), insularity (island or continent) and diet (herbivorous, carnivorous or omnivorous) from the IUCN red list website (IUCN 2025), Rhodin *et al.* (2021), Rhodin *et al.* (2025) and Bonin *et al.* (2006). Information on whether successful introductions of each species have occurred was also available in Rhodin *et al.* (2025). Country research rank was scored for each species based on the species distribution and Scimago country ranking in November 2025 (https://www.scimagojr.com/countryrank.php). The Scimago country rank is based on the number of articles published per country between 1996 and 2024. Species present in at least one of the top 10 countries were given a score of 3, a score of 2 if present in at least one of the top 10-20 countries, or a score of 1 if not present in any of the top 20 research producing countries. We considered the maximum straight-line carapace length (in cm) as body size metric, available for most species from Rhodin *et al.* (2021; 2025). In addition, we retrieved information on maximum longevity, generation length, female age at maturity, clutch size, incubation time and egg length from https://www.demogr.mpg.de/longevityrecords/0403.htm, Miller *et al.* (2001) Bonin *et al.* (2006) Myhrvold *et al.* (2015), Rodríguez-Caro *et al.* (2023). We extracted the risk of extinction and population trend of each assessed species from the IUCN's web site (IUCN 2025) in November 2025.

Analyses

We ran all the analyses using R (R Core Team 2024). To compare the different indices of research effort, we used Pearson correlation tests and coefficients on each pair of log-transformed indices (considering log (research effort +1)). To characterize the level of aggregation of research effort, we used the Poulin index of discrepancy (D, Poulin 1993). It allowed us to measure the deviation of research effort distribution from a theoretically even distribution among species. D ranges from zero to one, indicating no aggregation (0) to highly aggregated (1) distribution. This index was originally developed to quantify parasite aggregation across individuals in a host population. When investigating changes in D across decades, we excluded the species that had not yet been described in 1980. Research can only be dedicated to a given species after its description, and we aimed at using the same set of species to make the comparison meaningful. However, for the global estimate of discrepancy, we included all species. We used the QPweb software to calculate D (Reiczigel *et al.* 2019).

Phylogeny and taxonomy

We built a Phylogenetic Linear Mixed Model (PLMM) using the function MCMCglmm from the R package *MCMCglmm* (Hadfield 2010) to estimate the proportion of variance in research effort (log-transformed) explained by phylogeny. We included phylogeny as random effect in a model with research effort (log-transformed) as response variable, using the phylogeny from Thomson *et al.* (2021), which used nuclear DNA to infer the phylogenetic relationships of 80% of the recognized extant Testudines species. This analysis was therefore conducted on a subset of 275 species included in this phylogeny. We used a poorly informative inverse Wishart prior (V = 1, ν = 0.002) for the variances, following Hadfield (2010), and ran the model for 550001 iterations with a burn-in interval of 50000 to ensure satisfactory convergence, sampling one every 500 iterations, and checking that autocorrelation levels among samples was lower than 0.1. We assessed chain convergence by visually analyzing trace plots and running Gelman-Rubin convergence diagnostic on five chains (*gelman.diag* function from the package *coda*, Plummer *et al.* 2006). We calculated the intra-class coefficient (ICC) using the variance estimates of the model (with ICC = Vp / (Vp + Vr), where Vp = phylogenetic variance and Vr = residual variance).

To determine the proportion of variance in research effort explained by taxonomy, we built linear mixed models (LMM) with research effort (log + 1 transformed) as response variable, and either family or genus nested into family as random effects, using the *lme* function in the R package nlme (Pinheiro *et al.* 2023). We also built a null model (using the gls function) with no random effect and compared the AIC of these three models. We calculated the intra-class coefficient (ICC) of each taxonomic level using the variance estimates provided by the full model for both the family and genus effects.

Predictors of research effort

We investigated the association between research effort and 14 species characteristics, including biogeographic range, country research rank, range size (considering either the estimated indigenous (historical) range or current range size), insularity (binary variable separating island from continent species), habitat (factor with four levels: marine, freshwater, terrestrial or both freshwater and terrestrial), introduction (binary variable segregating successfully introduced species from other species), diet (herbivorous, carnivorous, omnivorous), maximum straight-line carapace length (SCL), maximum longevity, generation length, female age at maturity, clutch size, egg length and incubation time. We also ran an extra analysis focusing on either freshwater or terrestrial species to test whether the research effort varied between freshwater species occurring in rivers, still waterbodies or both rivers and still waterbodies, and between terrestrial species occurring in forests, open habitats, or both. We first tested for direct associations between research effort and each trait independently using Spearman rank correlations for continuous or ordinal variables (as some variables were not normally distributed), Wilcoxon tests for binary variables, or building linear models with research effort (log-transformed) as response variable and the factor of interest as explanatory variable for categorical variables (biogeographic range, habitat, and diet). This set of analyses allowed to maximize the sample size, as all the species with available information for the trait of interest could be included.

We then tested whether these associations were consistent when controlling for phylogeny (or whether phylogenetic non-independence alone could explain these associations), building one LMM per predictor with research effort (log-transformed) as response variable, the predictor of interest as explanatory variable and phylogeny as random effect. This analysis restricted the dataset to the 275 species included in the phylogeny. For categorical variables, we compared the deviance information criterion (DIC) of the model including vs excluding the categorical variable of interest to assess whether it was associated with research effort. The DIC is a variation of the AIC used in Bayesian model selection using MCMC simulations, and lower DIC indicate models with a better fit. Finally, we included all the predictors as fixed effects into one LMM with phylogeny as random effect to determine which predictors of research effort were the most important. We used a stepwise selection, removing non-significant predictors one by one until all predictors were significant (pMCMC < 0.05). We also used DIC comparisons of models including vs excluding each categorical variable to decide whether to include them or not in the final model (as MCMCglmm does not provide unique pMCMC values for categorical variables). Once a final model was selected, we added each removed parameter one by one to the model to validate it, determining whether previously removed variables were still non-significant (or resulted in models with a higher DIC, using a threshold of ΔDIC = 5). For this analysis, we scaled all continuous predictors to a mean of 0 and a variance of 1 to obtain comparable estimates. This final global model could only include the species with trait values available for all the traits considered, and therefore was conducted on a significantly decreased sample size. Female age at maturity and generation length strongly decreased the number of species on which to run this analysis, and were never significantly associated with research effort, so we excluded them from this last analysis to maximize the sample size. The correlation between

predictors was always < 0.4 except between maximum SCL and clutch size (Spearman ρ = 0.568, p < 0.001). To avoid collinearity issues, we excluded clutch size from this analysis. With regards to range size, we considered the estimated indigenous range size (AOO), available for all species in our dataset, since current range size was only available for 313 of the 370 species (the two metrics were highly correlated: Spearman ρ = 0.931). The dataset for this multivariate analysis considered 115 species for which all 11 traits were available. We reran this model selection after excluding the sea turtles to test for result consistency.

Extinction risk

We tested whether the research effort for IUCN-assessed species differed from the non-assessed species. Then, we tested whether extinction risk was associated with research effort, ranking the five IUCN extinction risk categories from 1 (critically endangered) to 5 (least concern) and excluding data deficient, extinct and non-assessed species (IUCN 2025). We also tested whether threatened (CR, EN or VU) and non-threatened (NT or LC) species had different research efforts, and whether population trend (with 0 = decreasing, 1 = stable or 2 = increasing, available from IUCN (2025) predicted research effort. To that aim, we built linear models with research effort (log-transformed) as response variable and either IUCN assessment (assessed or not), extinction risk, threatened (yes/no) or population trend as fixed effects. Considering extinction risk and population trend as continuous or as categorical variables resulted in the same conclusions so we only present the results considering them as continuous variables.

Results

Estimating research effort using species names in titles only or in titles, abstracts and keywords provided highly correlated coefficients (Pearson correlation coefficient between the log-transformed indices = 0.966; p < 0.001). Similarly, considering synonyms or focusing on current names resulted in highly correlated coefficients (Pearson = 0.973; p < 0.001). The research effort extracted from Scopus by Guedes *et al.* (2023) was also highly correlated with the indices obtained with references listed in the WoS (e.g., when considering all fields and synonyms, Pearson = 0.953; p < 0.001; n = 342 species). In the following analyses, we used the index considering species synonyms and all fields (not just the title) in the search for papers on WoS.

The search resulted in a total of 24442 articles on the Web of Science. The mean number of articles per species was 66 (SE = 16), and the median was six articles (range = 0 to 3441), suggesting that the distribution was highly right-skewed. The Poulin index of discrepancy confirmed that the distribution of research effort was highly aggregated (D = 0.873, CI = [0.835; 0.912]). Although there was a small decrease in D across time (Poulin D; 1980s: 0.904, CI = [0.877 to 0.932]; 1990s: 0.905, CI = [0.869; 0.929]; 2000s: 0.867, CI = [0.830; 0.900]; 2010s: 0.860, CI = [0.809 to 0.899]), the change in discrepancy across decades was minimal (from 0.904 to 0.860).

The green sea-turtle (*Chelonia mydas*, with 3441 articles) and the loggerhead sea-turtle (*Caretta caretta*, with 3334 articles) were the two species with the highest index of research effort, followed by the pond slider (*Trachemys scripta*, with 2157 articles), painted turtle (*Chrysemys picta*, with 1445 articles) and common snapping turtle (*Chelydra serpentina*, with 1082 articles). The next three species were all marine turtles, so that out of the seven extant species of marine turtles, five are among the eight most studied species of Testudines. Sea turtles represent 1.9% of all Testudines species but gathered 39% of all publications in this group, with the least studied sea turtle (*Natator depressus*) obtaining a research effort score of 105 articles, 17 times higher than the median score for all Testudines. The most studied terrestrial species was the desert tortoise (*Gopherus agassizii*,

ranking 11th with 454 articles). Thirty-two species had a score of 0, including species such as the enigmatic leaf turtle *Cyclemys enigmatica*, while the 50% least studied species gathered only 2% of the global research effort (475 articles for 185 species). Across decades, the number of articles published on turtles increased sharply: from 1067 in the 1980s to 9766 in the 2010s, i.e. 9.2 times more in the 2010s than in the 1980s (Figure 1). This increase was particularly important in marine turtles, which attracted 18.1 times more research in the 1980s as compared to the 2010s (Figure 1).

Taxonomic and phylogenetic variation

The model including genus nested in family (AIC = 1329) had a better fit than the one including family only (AIC = 1346) or the null model (AIC = 1402). Together, family and genus explained 58% of the variance in research effort (ICC = 0.49 for family, ICC = 0.09 for genus). Phylogeny accounted for 66% of the variance when including it as a random effect in a PGLMM considering the 275 species of Testudines included in the phylogeny (extracting the ICC for family and genus with this same subset of 275 species resulted in an ICC of 0.48 for family and 0.09 for genus – thus 57% in total).

More diverse families also attracted more research (the number of papers per family increased significantly with the number of species per family, Figure 2). However, species diversity within a given family was not significantly correlated with the mean research effort per species (Figure 2). Some families were investigated particularly frequently, especially the two sea turtle families, while others were particularly neglected, such as the Pelomedusidae, a family of African freshwater turtles with five articles per species on average (Table 1). Important variation in research effort across species within a given family was also noticeable (Figure 3).

Biogeographic, ecological and life history predictors of research effort

Range size was significantly correlated with research effort; species with a larger range attracted more research (using the estimated indigenous (historical) range, n = 370, Spearman $\rho = 0.347$, $p < 0.001$; using the current range, n = 313, Spearman $\rho = 0.44$, $p < 0.001$). In addition, species that have been successfully introduced outside of their native range had a higher research effort than the other species (Wilcoxon W = 4701.5, $p < 0.001$; mean research effort = 150.9 ± 48.5 articles for the 58 introduced species, 51.5 ± 16.2 for the 312 non-introduced species). Research effort also varied significantly across geographic realms (Sum sq = 250.18, F = 14.34, Df = 9, $p < 0.001$). In addition to species occupying multiple realms (mostly species occurring in both Central and North America, or marine species with broad distribution), species from Europe and North America were given more research whereas species from the Caribbean, Central America or Africa were much less studied (see Figure 4). In addition, research effort varied significantly with the research rank of countries where species occur (n = 370, Spearman $\rho = 0.436$, $p < 0.001$): research effort was highest for species occurring in at least one of the top 10 countries (n = 152, mean = 145, SE = 37), intermediate for species occurring in at least one of the top 10-20 countries (n = 53, mean = 25, SE = 4), and lowest for species absent from the top 20 countries (n = 165, mean = 9, SE = 1).

Research effort did not vary significantly between island and continental species (n = 370, Wilcoxon W = 6076, p = 0.082), but habitat was a significant predictor of research effort (n = 352, Sum sq = 140.81, F = 21.917, Df = 3, $p < 0.001$; Figure 5), with marine species being studied more frequently than species from any other habitat category (all $p < 0.001$). Research effort did not differ significantly between freshwater, terrestrial and mixed-habitat species (all $p > 0.138$). Focusing on

either freshwater or terrestrial species, the type of freshwater or terrestrial habitat did not significantly predict variation in research effort (freshwater: n = 126, Sum sq = 5.365, F = 1.243, Df = 2, p = 0.292; terrestrial: n = 54, Sum sq = 5.972, F = 1.573, Df = 2, p = 0.217).

Research effort did not vary across diet categories either (n = 257, Sum sq = 0.89, F = 0.174, Df = 2, p = 0.841). Considering life history traits, research effort increased significantly with body size (maximum SCL: n = 369, Spearman ρ = 0.210, p < 0.001), maximum longevity (n = 202, Spearman ρ = 0.339, p < 0.001) and clutch size (n = 275, Spearman ρ = 0.148, p = 0.014), decreased significantly with egg size (n = 189, Spearman ρ = -0.161, p = 0.026) but was not significantly correlated with generation length (n = 139, Spearman ρ = 0.017, p = 0.839), female age at maturity (n = 92, Spearman ρ = 0.151, p = 0.150) or incubation time (n = 212, Spearman ρ = -0.127, p = 0.065).

Building separate phylogenetic mixed models for each of these 14 predictors led to similar conclusions, except for the insularity effect which became significant – insular species had a significantly lower research effort than continental ones (pMCMC = 0.002) – and for incubation time as research effort decreased significantly with incubation time (pMCMC = 0.028) once correcting for phylogenetic non-independence (Table 2).

The best model with multiple predictors included five different predictors, in addition to the random effect of phylogeny: range size, introduction status, biogeographic realm, maximum SCL, and egg length. The direction of their effects was similar to those detected using univariate models (see Table 3). Excluding marine species resulted in the same best model and qualitatively similar results (see Suppl. Mat. 1).

Extinction risk

IUCN-assessed species had a higher research effort than non-assessed or data-deficient species (estimate = 0.904 ± 0.176, t = 5.123, p < 0.001; mean number of papers for assessed species = 86.2 ± 22.2; for non-assessed species = 14.8 ± 2.6). The research effort of threatened and non-threatened species did not differ significantly (estimate = -0.099 ± 0.214, t = -0.463, p = 0.644), and the risk of extinction did not significantly predict research effort (n = 160, estimate =0.104 ± 0.070, t = 1.486, p = 0.138). Similarly, research effort did not vary significantly with population trend (n = 177, estimate = 0.114 ± 0.234, t = 0.488, p = 0.626).

Discussion

Identifying taxonomic biases in research effort can help future research programs to aim at correcting these biases and improve our global understanding of turtles and tortoises. We underline here that Testudines show strong discrepancies in research effort across species, with the overwhelmingly high proportion of research dedicated to a small number of sea turtle species being particularly striking. Phylogeny itself explained 66% of the variance in research effort, also illustrating that some parts of the Testudines tree have been much more researched than others. Although overall, the number of publications focused on Testudines has increased substantially over the last decades, discrepancies across species have been maintained so that understudied species remained understudied while more studied species keep attracting more research. Our results also underline that these research-biases are not neutral with regards to species traits – instead, more research is dedicated towards species that share similar biogeographic, ecological and life history traits. Neither

high risk nor low risk species were particularly targeted by researchers since conservation status did not predict research effort.

Our analyses underline the high and non-neutral (with regards to species traits) heterogeneity in research effort across turtle species. This heterogeneity showed few changes over the last decades, as illustrated by the minimal decrease in research load discrepancy between the 1980s and 2010s. This is not unique to turtles, and has been described in other taxonomic groups, including birds (Ducatez & Lefebvre 2014), mammals (de Figueiredo Machado *et al.* 2023), fishes (Ducatez 2019; Guy *et al.* 2021) and amphibians (Silva *et al.* 2020). Although our result suggests a need for a reorientation of research efforts and funding towards less studied species, different explanations can help in interpreting it. First, some species are used as model organisms, responding to the need of acquiring in-depth knowledge of some species to address more advanced biological questions, and leading to outlier-species which attracted the attention of many researchers. *Trachemys scripta* is one such example: the pond slider has been the focus of research addressing questions in a diversity of disciplines, including molecular biology (e.g., Milton *et al.* 2006), development biology (Lin *et al.* 2024), physiology (Overgaard *et al.* 2002), ecology (e.g., Lindsay *et al.* 2013) and cognition (e.g., Sun *et al.* 2023), although understanding the impacts of this species that is invasive in part of its range also contributed to its success with researchers (e.g., see Balzani *et al.* 2016). The relatively small size of this species, its adaptability to captivity and its distribution (in both its native and introduced range) in areas with high scientific capacity made it an ideal model species to study both in the lab and *in situ*.

Second, some species are the target of research because of their sociocultural importance. The focus on charismatic species in conservation has been seen as a way to attract funding and public attention towards conservation issues, although this has been debated (Bowen-Jones & Entwistle 2002; Small 2012a; Small 2012b; Macdonald *et al.* 2015). From a global knowledge perspective, this focus on some flagship species targeted because of their charisma is more problematic, as it is likely to both concentrate the funding on a small subset of species, and lead to a lack of representativeness of the knowledge on a given taxonomic group such as the Testudines. For example, the particularly high amount of research dedicated to sea turtles is likely mostly explained by their cultural value (Kitolelei *et al.* 2022) and by the fascination that they represent for the public, increasing the probability of research funding attribution for this group of turtles. This can be interpreted as a positive outcome: our knowledge of sea turtles is particularly good, especially for species that are difficult to study given their marine lifestyle. In addition, the original habitat and migratory behaviors of sea turtles also increases the relevance of investigating these species. However, the contrast with non-marine species highlights the way research resources are distributed: on average, a sea-turtle species received 33 times more research than a freshwater or terrestrial species. The endangerment of sea turtle species (five are considered at risk of extinction, one is data deficient and one is considered Least Concern) together with their unique use of the marine habitat might be seen as justifications for the high effort put towards sea turtles. However, this is not specific to sea turtles: only 24% of all freshwater and terrestrial species are considered as least concern or near threatened, with the others being either at risk of extinction, not assessed or data deficient. The high proportion of freshwater and terrestrial species which risk of extinction has not been evaluated (109 species) is also a major concern, as under-studied species tend to have a higher risk of extinction than species that have been evaluated (Howard & Bickford 2014; Wotherspoon *et al.* 2024). Using sea-turtles as flagship species to attract public attention and funding towards other groups of turtles might be a strategy to fill some of these major knowledge gaps.

The ease of researcher access to a given species is also an important factor determining species knowledge production. This is likely reflected in the biogeographic traits that determined research effort in our analyses. Species with a larger range are more likely to include research facilities within their range and are therefore more often investigated, while continental species are often more accessible than insular ones (although the insularity effect was only significant when considered as single predictor in a LMM). Similarly, species distributed in areas with a higher scientific capacity, such as North America and Europe or any of the top 10 or top 20 most scientifically productive countries, are more studied than species from areas with more limited research funding such as Africa or Central America. This result is in line with the literature: for example, Guedes *et al.* (2023) found that reptile species which range overlaps with biodiversity institutions received more research attention. Some of the most important turtle biodiversity hotspots are in areas that are relatively neglected by researchers (Australasia, Africa, South America; Bonin *et al.* 2006). Similar patterns were detected in other taxonomic groups, including in reptiles (Guedes *et al.* 2023), birds (Ducatez & Lefebvre 2014) or sharks (Ducatez 2019), underlining that these research biases are not unique to turtles and tortoises. Therefore, conclusions of large-scale studies are likely to mostly apply for well-studied regions such as North America and Europe, but less so to understudied areas such as Central America or Africa. Overall, although targeting species that are easier to access is meaningful from both a financial and a logistic perspective, orienting research towards understudied areas, localized and/or insular species to correct these biases is highly relevant for turtles as for a diversity of other taxonomic groups.

Several life history traits were associated with research effort. These associations might be the result of differences in how easy it is to study species with particular traits. For example, larger species can be easier to detect and study, and often characterized by a longer lifespan, potentially explaining the positive association between research effort and longevity. Alternatively, for some traits, differences in research effort are likely to directly affect the trait estimates. For example, record longevities are more likely to be documented in intensely studied species than in less studied ones, leading to biased differences in the estimated species maximum longevities. Associations between research effort and trait values due to biased estimates or trait-based selection of researched species are likely to have consequences for the interpretation of comparative analyses and literature reviews. Such studies are often meant to be representative of entire taxonomic groups. They however tend to exclude poorly known species and/or consider biased or imprecise trait estimates due to species knowledge discrepancies, making their conclusions less general than expected. Small turtle and tortoise species with small eggs, small clutches and short longevity are, for example, less well known, and therefore likely to be more often excluded from global analyses, biasing any results to a subset of species that is not representative of the entire group. In contrast, maternal age at reproduction and generation length were not associated with research effort. However, these two traits were available for small numbers of species (110 and 86 species, see Table 2), and the lack of significant association might be due to a lack of statistical power. Overall, the importance of these knowledge differences in shaping the results of broad taxonomic scale analyses needs to be acknowledged in order to properly interpret their results.

Apart from the strong research bias towards marine species, ecological traits did not predict variation in research effort across species. When focusing on non-marine species, research effort did not vary according to Testudines habitats or diet. The lack of differences in research effort related to either habitat or diet is likely robust since these two traits were available for most species. It suggests that species with different habitats (with the exception of marine habitats) and diet have been relatively evenly investigated, contrasting with other taxonomic groups where species from some habitats tend

to be understudied. For example, deep-sea shark species were less often investigated than species occurring closer to the surface (Ducatez 2019).

Previous works have evidenced the importance of other traits as predictors of variation in research effort across species. Socioeconomic factors, including range overlap with biodiversity institutions, were for example shown to increase the amount of research dedicated to reptile species (Guedes *et al.* 2023), while recently described species tend to have received less research (Guedes *et al.* 2023). Aesthetic biases have also been demonstrated for example in plants where more colourful and conspicuous species attracted more research (Adamo *et al.* 2021). The use of anthropic habitats also tends to increase research attention, including in amphibians (Silva *et al.* 2020). Such parameters may also influence research effort in turtles and tortoises and could be considered in future studies.

The number of publications per species did not vary with either extinction risk or population trend across Testudines species. Removing sea turtles from the analyses did not change this result (additional results). Similarly, considering a categorical variable instead of a continuous one to represent either extinction risk or population trend led to the same conclusion. This result contrasts with several other taxonomic groups, where researchers tend to focus more on low risk species (e.g., in Amphibians, Silva *et al.* 2020; in birds, Ducatez & Lefebvre 2014) or species with intermediate risk levels (e.g., in sharks, Ducatez 2019). This relatively even distribution of research attention across extinction risk categories may be considered as a positive result, especially as compared to other taxa: endangered species are not relatively understudied. However, a special research focus on endangered species would be desirable to improve conservation practices on species that need it the most. A study focusing on research effort on turtles and tortoises in conservation science (rather than all publications) and its distribution according to species risk of extinction would be of interest to better assess the needs for conservation research in endangered species (see for example this study on primates: de Figueiredo Machado *et al.* 2023). The (expected) much lower number of publications on the 110 species that were either not assessed or considered data deficient (as compared to species which extinction risk has been assessed) also raises important concerns, as these species tend to be at a higher risk of extinction according to work conducted on other taxonomic groups (Howard & Bickford 2014; Wotherspoon *et al.* 2024). Overall, species that are at high risk of extinction, as well as species which extinction risk has not been assessed yet, or which extinction risk could not be assessed because of data deficiency, should be the focus of more research to optimize our chances of implementing appropriate conservation actions for the entire clade. Future work investigating the association between research effort and conservation funding per species would also provide valuable information on how indicative of conservation funding indices of research efforts can be.

Assessing the amount of research dedicated to a given species requires to make choices with regards to the sources and criteria considered. Including one or several databases, considering or not the grey literature, accounting for name changes or quantifying the amount of research dedicated to a given species within publications are among the important factors likely to affect conclusions. The index we are using cannot be directly interpreted as an absolute measure of the global research effort that was dedicated to a given species. Studies published before 1975 were not included in this index, and grey literature as well studies not included in the WoS were excluded when calculating this index. In addition, even when ignoring these limitations, the number of articles mentioning a species name in its title or abstract is a rough proxy for research effort: the amount of published research and knowledge provided on a given species can vary substantially from one article to another, and using the number of articles is an important shortcut. Other indices, including information on species knowledge (e.g., trait value knowledge, number of individuals measured,

duration of studies) would provide complementary information on how much research was invested in a given species. However, these limitations are mostly important when aiming at obtaining absolute indices able to capture all or most research that has been conducted on a given species. Here, our purpose was to obtain a metric that allows comparisons of research effort across species, i.e., to evaluate the amount of research attributed to a given species relative to others. To that aim, we demonstrated that the choice of one index over another had little importance for Testudines, since all tested indices were highly correlated. Including synonyms or not, considering all fields or only the titles during the search for papers, and considering the Web of Science or Scopus databases resulted in comparable numbers of publications per species (all Pearson coefficients > 0.94) – a pattern similar to other taxonomic groups (Ducatez & Lefebvre 2014; Ducatez 2019). That being said, we recommend using the index that takes into account synonyms (see Correia *et al.* 2018) and all fields (rather than focusing on articles where the species names appears in the title), as it is more inclusive.

By identifying both the most and least studied families and species of Testudines, we hope that this work will help orienting research towards taxa and regions that require more research. Families such as the Pelomedusidae, with only five articles per species on average (Table 1), would, for example, warrant much more research. Although having model species is relevant and allows addressing fundamental questions more in depth, the lack of knowledge on a significant proportion of the Testudines is likely to affect our interpretation of their global ecological importance and our ability to preserve them. More research on endangered species, including in areas such as South East Asia that concentrate a high proportion of high risk or unassessed species (Mittermeier *et al.* 2015), but also to understudied areas such as Africa and Central America, would be especially rewarding by both increasing our knowledge of this unique taxonomic group and identifying actions that could help improve the conservation status of endangered species. Sharing funding allocated to sea turtle research and conservation might also be a way forward to fill the knowledge gaps that are likely to prevent effective conservation actions for terrestrial and freshwater species that also play key roles in their ecosystems. By providing an index of research effort for all species of Testudines, we also hope to help account for potential biases related to discrepancies in research effort across species in global reviews and comparative analyses.

References

ADAMO, M., CHIALVA, M., CALEVO, J., BERTONI, F., DIXON, K. & MAMMOLA, S. 2021. Plant scientists' research attention is skewed towards colourful, conspicuous and broadly distributed flowers. *Nature Plants* 7: 574–578. doi:10.1038/s41477-021-00912-2

BALZANI, P., VIZZINI, S., SANTINI, G., *et al.* 2016. Stable isotope analysis of trophic niche in two co-occurring native and invasive terrapins, *Emys orbicularis* and *Trachemys scripta elegans*. *Biological Invasions* 18: 3611–3621. doi:10.1007/s10530-016-1251-x

BONIN, F., DEVAUX, B. & DUPRÉ, A. 2006. *Turtles of the World*. New-York, USA: Johns Hopkins University Press.

BOWEN-JONES, E. & ENTWISTLE, A. 2002. Identifying appropriate flagship species: the importance of culture and local contexts. *Oryx* 36: 189–195. doi:10.1017/S0030605302000261

CHEN, T.-H., CHANG, H.-C. & LUE, K.-Y. 2009. Unregulated trade in turtle shells for Chinese traditional medicine in East and Southeast Asia: the case of Taiwan. *Chelonian Conservation and Biology* 8: 11–18. doi:10.2744/CCB-0747.1

CHEUNG, S.M. & DUDGEON, D. 2006. Quantifying the Asian turtle crisis: market surveys in southern China, 2000–2003. *Aquatic Conservation: Marine and Freshwater Ecosystems* 16: 751–770. doi:10.1002/aqc.803

CHRISTIANEN, M.J.A., SMULDERS, F.O.H., VONK, J.A., *et al.* 2023. Seagrass ecosystem multifunctionality under the rise of a flagship marine megaherbivore. *Global Change Biology* 29: 215–230. doi:10.1111/gcb.16464

CORREIA, R.A., JARIĆ, I., JEPSON, P., MALHADO, A.C.M., ALVES, J.A. & LADLE, R.J. 2018. Nomenclature instability in species culturomic assessments: Why synonyms matter. *Ecological Indicators* 90: 74–78. doi:10.1016/j.ecolind.2018.02.059

DUCATEZ, S. 2019. Which sharks attract research? Analyses of the distribution of research effort in sharks reveal significant non-random knowledge biases. *Reviews in Fish Biology and Fisheries* 29: 355–367. doi:10.1007/s11160-019-09556-0

DUCATEZ, S. & LEFEBVRE, L. 2014. Patterns of research effort in birds. *PLoS ONE* 9: e89955. doi:10.1371/journal.pone.0089955

FALCÓN, W. & HANSEN, D.M. 2018. Island rewilding with giant tortoises in an era of climate change. *Philosophical Transactions of the Royal Society B: Biological Sciences* 373: 20170442. doi:10.1098/rstb.2017.0442

FALCÓN, W., MOLL, D. & HANSEN, D.M. 2020. Frugivory and seed dispersal by chelonians: a review and synthesis. *Biological Reviews* 95: 142–166. doi:10.1111/brv.12558

DE FIGUEIREDO MACHADO, F., ROCHA, B.S., BRITO, D. & TERRIBILE, L.C. 2023. Trends and biases in research efforts for primate conservation: threatened species are not in the spotlight. *Perspectives in Ecology and Conservation* 21: 286–293. doi:10.1016/j.pecon.2023.10.001

FROYD, C.A., COFFEY, E.E.D., VAN DER KNAAP, W.O., VAN LEEUWEN, J.F.N., TYE, A. & WILLIS, K.J. 2014. The ecological consequences of megafaunal loss: giant tortoises and wetland biodiversity. *Ecology Letters* 17: 144–154. doi:10.1111/ele.12203

GRIFFITHS, C.J., HANSEN, D.M., JONES, C.G., ZUËL, N. & HARRIS, S. 2011. Resurrecting extinct interactions with extant substitutes. *Current Biology* 21: 762–765. doi:10.1016/j.cub.2011.03.042

GUEDES, J.J.M., MOURA, M.R. & ALEXANDRE F. DINIZ-FILHO, J. 2023. Species out of sight: elucidating the determinants of research effort in global reptiles. *Ecography* 2023: e06491. doi:10.1111/ecog.06491

GUY, C.S., COX, T.L., WILLIAMS, J.R., *et al.* 2021. A paradoxical knowledge gap in science for critically endangered fishes and game fishes during the sixth mass extinction. *Scientific Reports* 11: 8447. doi:10.1038/s41598-021-87871-y

HADFIELD, J.D. 2010. MCMC Methods for Multi-Response Generalized Linear Mixed Models: The MCMCglmm R Package. *Journal of Statistical Software* 33: 1–22. doi:10.18637/jss.v033.i02

HANSEN, D.M., DONLAN, C.J., GRIFFITHS, C.J. & CAMPBELL, K.J. 2010. Ecological history and latent conservation potential: large and giant tortoises as a model for taxon substitutions. *ECOGRAPHY* 33: 272–284. doi:10.1111/j.1600-0587.2010.06305.x

HOWARD, S.D. & BICKFORD, D.P. 2014. Amphibians over the edge: silent extinction risk of Data Deficient species. *Diversity and Distributions* 20: 837–846. doi:10.1111/ddi.12218

IUCN 2025. The IUCN Red List of Threatened Species Version 2025-1.

JONES, C.G., TATAYAH, V., MOORHOUSE-GANN, R., GRIFFITHS, C., ZUEL, N. & COLE, N. 2023. *Slow and Steady Wins the Race: Using Non-native Tortoises to Rewild Islands off Mauritius*. M. J. Gaywood, J. G. Ewen, P. M. Hollingsworth, & A. Moehrenschlager (Eds.). Cambridge: Cambridge Univ Press.

KITOLELEI, S., SODERBERG, A., QAQARA, N., *et al.* 2022. Conservation status and cultural values of sea turtles leading to (un)written parallel management systems in Fiji. *Ambio* 51: 2431–2444. doi:10.1007/s13280-022-01766-4

LENTH, R. 2024. emmeans: Estimated marginal means, aka least-squares means. *R package version 1.10.5*.

LIN, J., ZHANG, M., LIANG, F., *et al.* 2024. Morphological and transcriptomic analyses of embryonic development of red-eared slider *Trachemys scripta elegans*. *Animal Reproduction Science* 261: 107395. doi:10.1016/j.anireprosci.2023.107395

LINDSAY, M.K., ZHANG, Y., FORSTNER, M.R.J. & HAHN, D. 2013. Effects of the freshwater turtle *Trachemys scripta elegans* on ecosystem functioning: an approach in experimental ponds. *Amphibia-Reptilia* 34: 75–84. doi:10.1163/15685381-00002871

LOVICH, J.E., ENNEN, J.R., AGHA, M. & GIBBONS, J.W. 2018. Where have all the turtles gone, and why does it matter? *BioScience* 68: 771–781. doi:10.1093/biosci/biy095

MACDONALD, E.A., BURNHAM, D., HINKS, A.E., DICKMAN, A.J., MALHI, Y. & MACDONALD, D.W. 2015. Conservation inequality and the charismatic cat: *Felis felicis*. *Global Ecology and Conservation* 3: 851–866. doi:10.1016/j.gecco.2015.04.006

MILLER, J.D., LIMPUS, C.J. & GODFREY, M.H. 2001. Nest Site Selection, Oviposition, Eggs, Development, Hatching, and Emergence of Loggerhead Turtles. Biology and conservation of loggerhead sea turtle. University of Florida Press

MILTON, S.L., NAYAK, G., LUTZ, P.L. & PRENTICE, H.M. 2006. Gene transcription of neuroglobin is upregulated by hypoxia and anoxia in the brain of the anoxia-tolerant turtle *Trachemys scripta*. *Journal of Biomedical Science* 13: 509–514. doi:10.1007/s11373-006-9084-8

MITTERMEIER, R.A., DIJK, P.P. van, RHODIN, A.G.J. & NASH, S.D. 2015. Turtle hotspots: an analysis of the occurrence of tortoises and freshwater turtles in biodiversity hotspots, high-biodiversity wilderness areas, and turtle priority areas. *Chelonian Conservation and Biology* 14: 2–10. doi:10.2744/ccab-14-01-2-10.1

MYHRVOLD, N.P., BALDRIDGE, E., CHAN, B., SIVAM, D., FREEMAN, D.L. & ERNEST, S.K.M. 2015. An amniote life-history database to perform comparative analyses with birds, mammals, and reptiles. *Ecology* 96: 3109–3109. doi:10.1890/15-0846R.1

OVERGAARD, J., STECYK, J.A.W., FARRELL, A.P. & WANG, T. 2002. Adrenergic control of the cardiovascular system in the turtle *Trachemys scripta*. *The Journal of Experimental Biology* 205: 3335–3345. doi:10.1242/jeb.205.21.3335

PINHEIRO, J., BATES, D. & R CORE TEAM 2023. nlme: Linear and Nonlinear Mixed Effects Models.

PLUMMER, M., BEST, N., COWLES, K. & VINES, K. 2006. CODA: Convergence diagnosis and output analysis for MCMC. *R News* 6: 7–11.

POULIN, R. 1993. The disparity between observed and uniform distributions: A new look at parasite aggregation. *International Journal for Parasitology* 23: 937–944. doi:10.1016/0020-7519(93)90060-C

R CORE TEAM 2024. R: A Language and Environment for Statistical Computing.

REICZIGEL, J., MAROZZI, M., FÁBIÁN, I. & RÓZSA, L. 2019. Biostatistics for parasitologists – a primer to quantitative parasitology. *Trends in Parasitology* 35: 277–281. doi:10.1016/j.pt.2019.01.003

RHODIN, A.G.J., IVERSON, J.B., BOUR, R., *et al.* 2021. Turtles of the World: Annotated checklist and atlas of taxonomy, synonymy, distribution, and conservation status (9th Ed.). *Chelonian Research Monographs* 8: 1–472. doi:10.11606/issn.2316-9079.v20i2p225-228

RHODIN, A.G.J., IVERSON, J.B., FRITZ, U., *et al.* 2025. Turtles of the World: Annotated checklist and atlas of taxonomy, synonymy, distribution, and conservation status (10th Ed.). *Chelonian Research Monographs* 10. doi:10.11606/issn.2316-9079.v20i2p225-228

RODRÍGUEZ-CARO, R.C., GRACIÁ, E., BLOMBERG, S.P., *et al.* 2023. Anthropogenic impacts on threatened species erode functional diversity in chelonians and crocodilians. *Nature Communications* 14: 1542. doi:10.1038/s41467-023-37089-5

SANTOS, J.W. dos, CORREIA, R.A., MALHADO, A.C.M., *et al.* 2020. Drivers of taxonomic bias in conservation research: a global analysis of terrestrial mammals. *Animal Conservation* 23: 679–688. doi:10.1111/acv.12586

SEMINOFF, J.A. 2025. The global turtle crisis—a wildlife trafficking challenge like no other. *Chelonian Conservation and Biology* 23: 135–136. doi:10.2744/1071-8443-23.2.1

SHEARER, I. & TÜRKOZAN, O. 2025. Global *Testudo* trade: update and recent trends. *Chelonian Conservation and Biology* 23: 161–168. doi:10.2744/CCB-1643.1

SILVA, A.F. da, MALHADO, A.C.M., CORREIA, R.A., LADLE, R.J., VITAL, M.V.C. & MOTT, T. 2020. Taxonomic bias in amphibian research: Are researchers responding to conservation need? *Journal for Nature Conservation* 56: 125829. doi:10.1016/j.jnc.2020.125829

SMALL, E. 2012a. The new Noah's Ark: beautiful and useful species only. Part 1. Biodiversity conservation issues and priorities. *Biodiversity* 12: 232–247. doi:10.1080/14888386.2011.642663

SMALL, E. 2012b. The new Noah's Ark: beautiful and useful species only. Part 2. The chosen species. *Biodiversity* 13: 37–53. doi:10.1080/14888386.2012.659443

STANFORD, C.B., IVERSON, J.B., RHODIN, A.G.J., *et al.* 2020. Turtles and tortoises are in trouble. *Current Biology* 30: R721–R735. doi:10.1016/j.cub.2020.04.088

SUN, X., PIAO, Y., WANG, T., WANG, J., FU, J. & CUI, J. 2023. Keep numbers in view: red-eared sliders (*Trachemys scripta elegans*) learn to discriminate relative quantities. *Biology Letters* 19: 20230203. doi:10.1098/rsbl.2023.0203

SVENNING, J.-C., PEDERSEN, P.B.M., DONLAN, C.J., *et al.* 2016. Science for a wilder Anthropocene: Synthesis and future directions for trophic rewilding research. *Proceedings of the National Academy of Sciences* 113: 898–906. doi:10.1073/pnas.1502556112

TAPIA AGUILERA, W. & GIBBS, J.P. 2023. Rewilding giant tortoises engineers plant communities at local to landscape scales. *Conservation Letters* 16: e12968. doi:10.1111/conl.12968

TAPIA, W., GOLDSPIEL, H.B. & GIBBS, J.P. 2022. Introduction of giant tortoises as a replacement “ecosystem engineer” to facilitate restoration of Santa Fe Island, Galapagos. *Restoration Ecology* 30: e13476. doi:10.1111/rec.13476

THOMSON, R.C., SPINKS, P.Q. & SHAFFER, H.B. 2021. A global phylogeny of turtles reveals a burst of climate-associated diversification on continental margins. *Proceedings of the National Academy of Sciences* 118: e2012215118. doi:10.1073/pnas.2012215118

WOTHERSPOON, L., DE OLIVEIRA CAETANO, G.H., ROLL, U., *et al.* 2024. Inferring the extinction risk of Data Deficient and Not Evaluated Australian squamates. *Austral Ecology* 49: e13485. doi:10.1111/aec.13485

Table 1. Species richness, mean research effort per species and total research effort per family in Testudines.

	Number of species	Mean number of papers	Total number of papers	Standard error
CHELONIIDAE	6	1429	8575	628
DERMOCHELYIDAE	1	1062	1062	
CHELYDRIDAE	5	243	1213	211
EMYDIDAE	58	111	6410	45
CARETTOCHELYIDAE	1	54	54	
PODOCNEMIDIDAE	8	50	402	21
PLATYSTERNIDAE	1	50	50	
TESTUDINIDAE	58	50	2874	13
TRIONYCHIDAE	35	32	1119	17
DERMATEMYDIDAE	1	29	29	
KINOSTERNIDAE	34	16	548	6
GEOEMYDIDAE	75	15	1138	3
CHELIDAE	60	14	833	3
PELOMEDUSIDAE	27	5	135	2

Table 2. Association between research effort and 14 predictors in Testudinidae, after taking phylogenetic relationships into account. Research effort was included as response variable in 14 different PLMM that included phylogeny as random effect and either one of the 14 predictors as fixed effects. For continuous or binary predictors, pMCMC was used to assess significance (a). A positive effect of introduction means that species that were introduced successfully had a higher research effort; a negative effect of insularity means that insular species had a lower research effort than continental ones. For categorical variables, DIC-based comparisons of models including or excluding each categorical predictor are shown (b) as MCMCglmm does not provide unique pMCMC values for these variables (a negative ΔDIC indicates that the predictor improved the fit of the model). SCL = Straight-line Carapace Length; pm = posterior mean; CI = credibility interval, eff samp = effective sample size, n = number of species included. Significant effects are in bold style.

Binary or continuous variables	pm	CI	eff samp	pMCMC	n
**Historic range size (AOO)**	**0.757**	**[0.631; 0.929]**	**1001**	**<0.001**	**275**
**Current range size**	**0.864**	**[0.705; 1.005]**	**1001**	**<0.001**	**251**
**Introduction**	**1.161**	**[0.748; 1.570]**	**1001**	**<0.001**	**275**
**Insularity**	**-1.144**	**[-1.840; -0.447]**	**1001**	**0.002**	**275**
**Country research rank**	**0.731**	**[0.548; 0.918]**	**1001**	**<0.001**	**275**
**Max SCL**	**0.015**	**[0.005; 0.024]**	**1001**	**0.008**	**274**
**Max longevity**	**0.018**	**[0.009; 0.027]**	**909.6**	**<0.001**	**185**
Generation length	-0.007	[-0.033; 0.018]	1001	0.601	110
Maternal age at maturity	0.026	[-0.312; 0.440]	1190	0.897	85
**Clutch size**	**0.028**	**[0.012; 0.042]**	**1001**	**<0.001**	**246**
**Egg length**	**-0.028**	**[-0.054; -0.003]**	**1001**	**0.028**	**172**
**Incubation time**	**-0.006**	**[-0.011; -0.001]**	**1001**	**0.028**	**194**

Categorical variables	DIC with	DIC without	ΔDIC	n
**Biogeographic realm**	**909**	**949**	**-40**	**275**
Habitat	950	944	6	273
Diet	786	783	3	229

Table 3. Best multi-predictor model explaining variation in research effort across species of Testudinidae. Phylogeny was included as random effect, and we used the MCMCglmm procedure. Non-significant predictors excluded from the best model (pMCMC > 0.01 or ΔDIC > 5) are shown in italic style below the factors included in the best model. SCL = Straight-line Carapace Length; pm = posterior mean; CI = credibility interval, eff.samp = effective sample size. 115 species were included in this analysis.

Explanatory variable	pm	95% CI	eff.samp	pMCMC
Intercept	2.269	[0.474; 4.226]	1125.8	0.018
Historic range size	0.668	[0.408; 0.941]	1205.5	<0.001
Introduction	0.463	[0.025; 0.905]	1001	0.042
Maximum SCL	0.854	[0.470; 1.184]	900.8	<0.001
Egg length	-0.433	[-0.734; -0.133]	1001	0.004
Biogeographic realm (ΔDIC = -32)				
Maximum longevity	*0.184*	*[-0.059; 0.396]*	*1001*	*0.118*
Insularity	*0.309*	*[-1.240; 1.734]*	*908.9*	*0.691*
Incubation	*-0.029*	*[-0.331; 0.211]*	*1001*	*0.859*
Country research rank	*0.035*	*[-0.318; 0.330]*	*1001*	*0.839*
Diet (ΔDIC = 8)				
Habitat (ΔDIC = 27)				

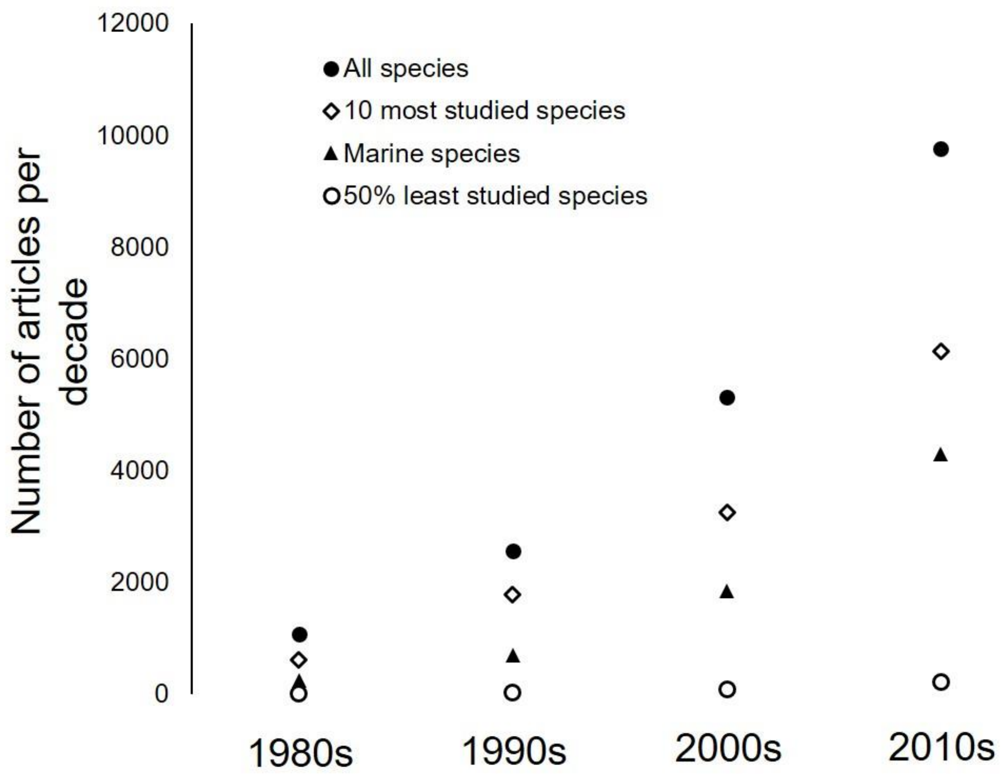

Figure 1. Evolution of the number of articles on turtle species across time for all turtle species, for the 10 most studied species, for marine species only, and for the 50% least studied species.

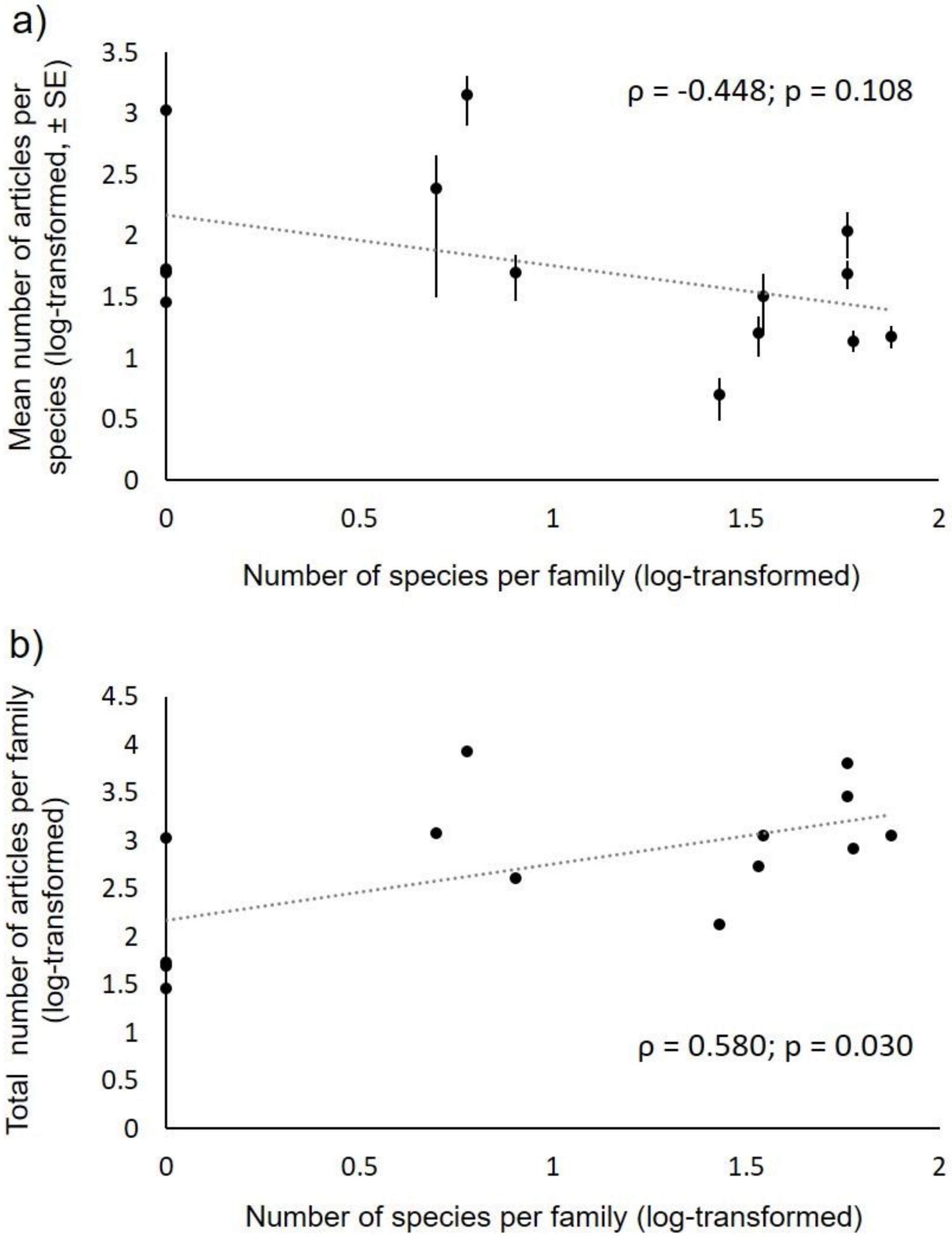

Figure 2. Association between the number of articles listed in the WoS (either the mean number in panel a, or the total number in panel b) and the number of species per family of Testudines. The results of Pearson tests are given (ρ = correlation coefficient; p = p-value) for each association.

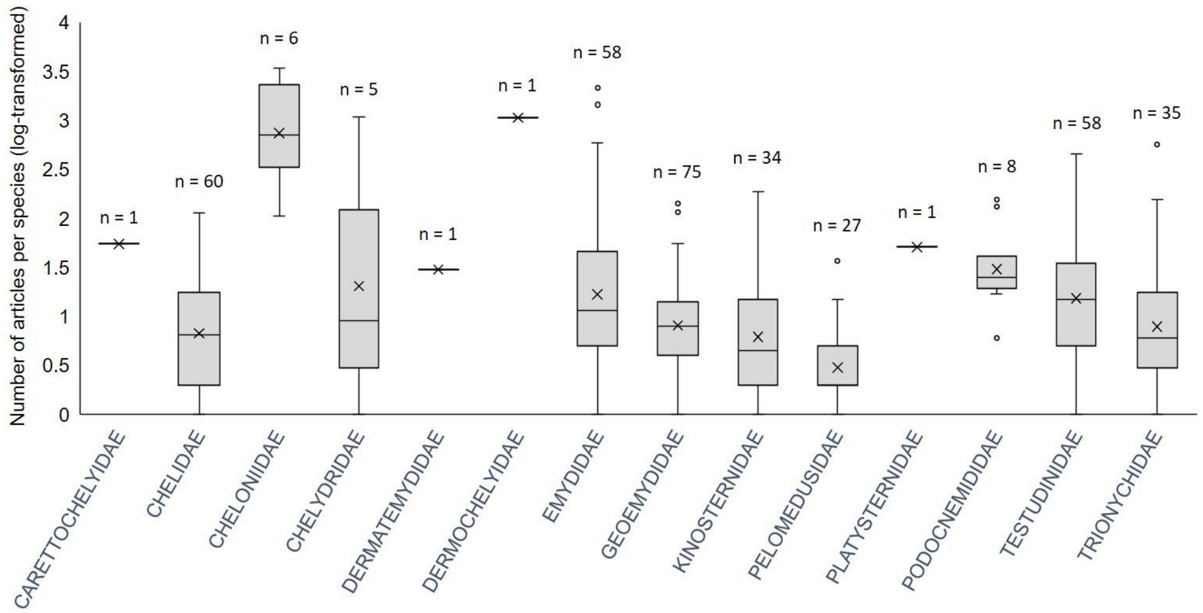

Figure 3. Box plot of the distribution of the number of articles (log-transformed) per species within each family of Testudines. Minimum, maximum, median, 25th and 75th quartiles are shown, as well as outliers. The number of species per family is indicated above each box, and x-symbols show the mean for each family.

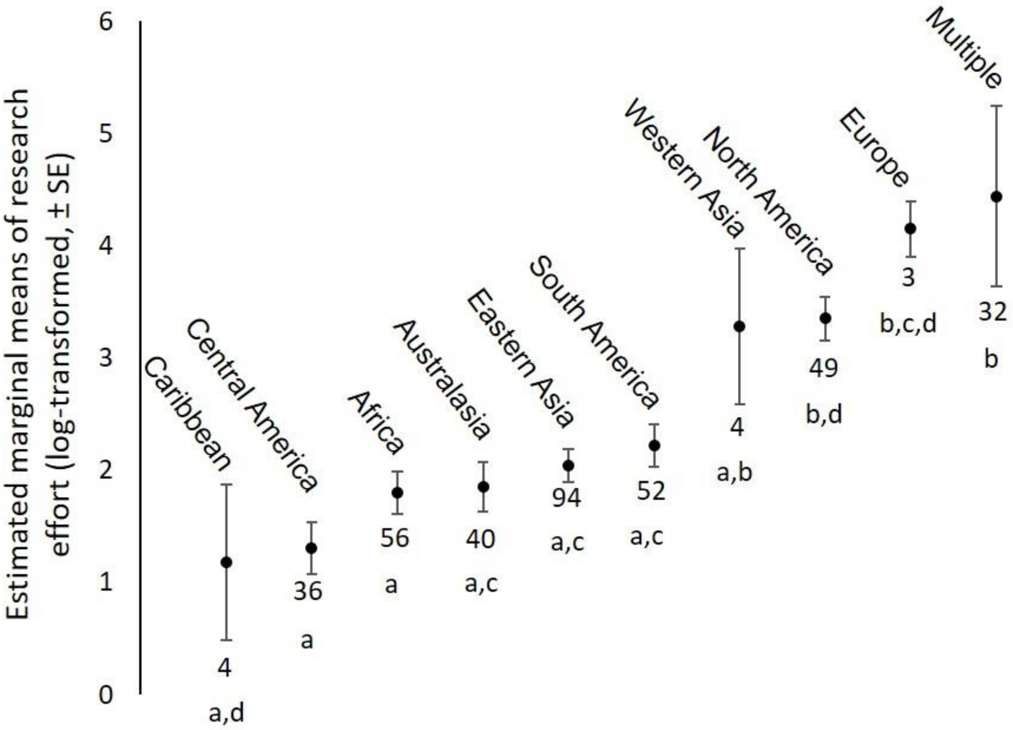

Figure 4. Variation in research effort across biogeographic realm. Estimated marginal means were extracted using the emmeans function (Lenth 2024). Different letters indicate significant differences between geographic realms (e.g., Central America and Europe), whereas research effort does not differ significantly between regions that share the same letter (e.g., all realms with an a).

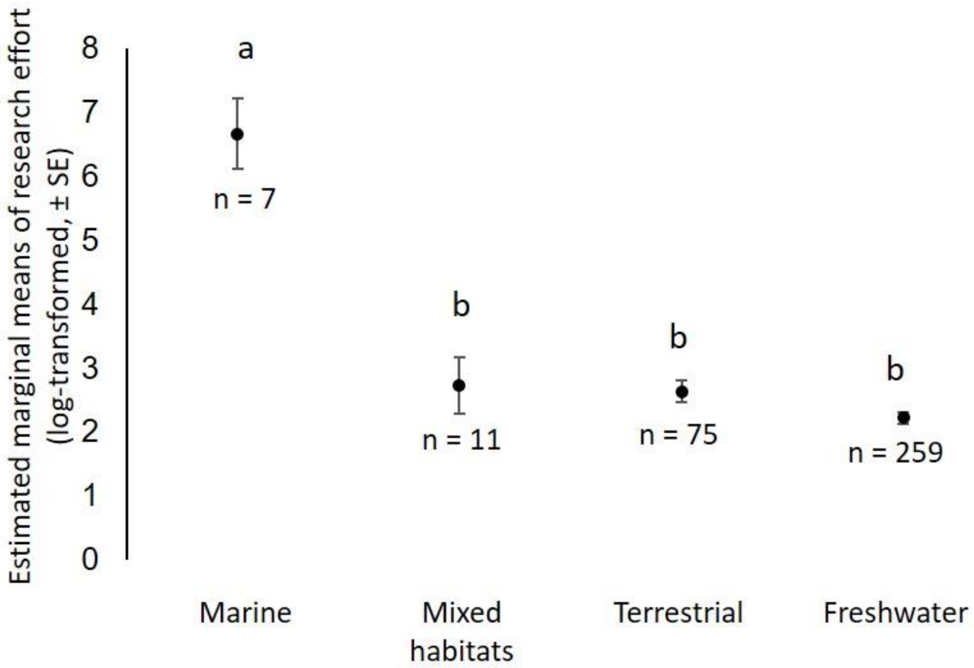

Figure 5. Variation in turtle research effort across habitats. Marine species were significantly more studied than turtles from any other habitat category, including species occurring in multiple habitats. The letters a and b indicate the significance ($p < 0.05$) of between-habitats comparisons (if the difference is significant, the two habitats were attributed different letters; if non-significant, they share the same letter). Different letters indicate significant differences between geographic realms (e.g., Central America and Europe), whereas research effort does not differ significantly between regions that share the same letter (e.g., all realms with an a).

Supplementary material 1. Best multi-predictor model explaining variation in research effort across species of Testudinidae after excluding marine species. Phylogeny was included as random effect, and we used the MCMCglmm procedure. Non-significant predictors excluded from the best model (pMCMC > 0.01 or ΔDIC > 5) are shown in italic style below the factors included in the best model. SCL = Straight-line Carapace Length; pm = posterior mean; CI = credibility interval, eff.samp = effective sample size. 110 species were included in this analysis.

Explanatory variables	pm	CI	eff.samp	pMCMC
Intercept	1.985	[0.439; 3.537]	1001	0.020
Historic range size	0.673	[0.391; 0.904]	868.1	<0.001
Introduction	0.479	[0.023; 0.964]	1001	0.048
Maximum SCL	0.449	[0.161; 0.752]	1001	0.002
Egg length	-0.444	[-0.747; -0.111]	1001	0.008
Biogeographic realm (ΔDIC = -21)				
Maximum longevity	*0.183*	*[-0.035; 0.434]*	*754.1*	*0.130*
Insularity	*0.273*	*[-1.269; 1.734]*	*1001*	*0.721*
Incubation	*-0.038*	*[-0.299; 0.333]*	*1001*	*0.761*
Country research rank	*0.010*	*[-0.299; 0.333]*	*1001*	*0.949*
Diet (ΔDIC = 10)				
Habitat (ΔDIC = 5)				

Supplementary material 2. Dataset including the research effort indices and traits considered in the analyses.

Supplementary material 3. R code used for the conducted analyses

Data Accessibilty Statement: The full dataset is available as Supplementary Material.

Competing Interests Statement: The authors declare no competing interest.

Author Contributions: SD: Writing – review & editing, Writing – original draft, Visualization, Methodology, Investigation, Funding acquisition, Formal analysis, Data curation, Conceptualization. **JD**: Writing – review & editing, Visualization, Methodology, Funding acquisition, Conceptualization

Acknowledgements: We are grateful to Tomasz Szczygielski and an anonymous reviewer for comments on a previous version of the manuscript.

Supporting Information 2 - dataset

Click here to access/download

Supporting Information

ResearchEffortTestudines.xlsx

Click here to access/download
Supporting Information
Supplementary Material 3 script REturtles.r

Which turtle should I study? Uneven distribution of research effort across Testudines species

Authors: Simon Ducatez & Jayna Lynn DeVore

Formatted: English (United States)

Simon Ducatez

Affiliation: UMR 241 SECOPOL (IRD, IFREMER, ILM, UPF)

Jayna L. DeVore

Affiliation: UMR 241 SECOPOL (UPF, IRD, IFREMER, ILM, UPF)

Corresponding author: Simon Ducatez, simon.ducatez@gmail.com

Abstract

Understanding why some species are more often studied than others can provide valuable information on ~~the~~ potential biases that research effort discrepancies can cause~~,~~ and help to direct~~ing~~ future research to mitigate these biases. Turtles form an order of~~r~~ reptiles in which the~~a~~ majority of species (67~~70~~%) are threatened with extinction, ~~and~~ but they show high variation in the amount of research devoted to each species. We aimed ~~at~~ to quantify~~ing~~ this variation across all ~~363~~ recognized extant species, and ~~at~~ to decipher~~ing~~ how this~~e~~ distribution of this research effort varied according to species taxonomy, phylogeny, distribution, ecology, life history and extinction risk. Using the number of references listed on the Web of Science for each species as a proxy for species research effort, we first show that this index ~~was~~ is both robust to ~~the~~ variation in search methods within WoS, and consistent with an index extracted from another online library (Scopus). The number of articles per species varied substantially, with 3441 articles for the green sea turtle alone, but only 475~~84~~ total articles for all 50% least studied species taken together. This heterogeneity was consistent across time since the 1970s. Phylogenetic non-independence explained 66% of the variance in research effort (vs 58% for taxonomy). In addition, marine species, a group that only includes seven species, were particularly highly studied: five of the eight most studied Testudines are marine turtles. In contrast, f~~F~~reshwater and terrestrial species shared similar research effort values. Species occurring in Europe and North America were also more studied than species from, e.g., Central America or Africa, as were species that occur~~ring~~ in the most scientifically active countries. In addition, species with a larger distribution range or introduced outside of their native range, ~~and~~ as well as species ~~that~~ with certain life history characteristics like ~~produce~~ larger clutches, smaller eggs or ~~with~~ a longer longevity were more ~~often~~ frequently investigated. In contrast, population trend and extinction risk did not predict research effort, although species that have not been assessed by the IUCN were characterized by a low research effort. This study underlines the importance of ~~taking into account~~accounting for the heterogeneity in research effort across turtle species in global analyses and helps to identify taxa, regions, and life history strategies that should be the focus of more research.

Keywords: Chelonian, research bias, tortoise, turtle

Introduction

~~Testudines emerged during the Triassic and survived two mass extinction crises that eliminated several other groups of reptiles at the end of the Permian and Cretaceous. Unfortunately, however, they seem poorly resilient~~Particularly sensitive to the ~~in front of~~ current extinction crisis (Lovich *et al.* 2018; Stanford *et al.* 2020), Testudines are one of the most threatened groups of vertebrates ~~and are currently one of the most threatened groups of vertebrates~~: 67~~70~~% of the assessed extant species are threatened with extinction (IUCN 2025), as compared to 25% of mammals, 20% of s~~S~~quamates and 14% of birds. Although several threats are responsible for this pattern, wildlife trafficking (for pet trade, turtle meat or body parts) was identified as the most damaging threat driving many species towards extinction (Stanford *et al.* 2020). Often referred to as the "Asian turtle crisis" because of how important this threat is in Asia (Cheung & Dudgeon 2006; Chen *et al.* 2009), it has recently been named a "Global turtle crisis" (Seminoff 2025) given the universal nature of turtle trafficking (e.g., see Shearer & Türkozan 2025). Testudines occupy terrestrial, freshwater and marine ecosystems, where they participate in a diversity of ecosystem functions, including herbivory, seed dispersal and detritivory (Froyd *et al.* 2014; Falcón & Hansen 2018; Falcón *et al.* 2020; Christianen *et al.* 2023; Tapia Aguilera & Gibbs 2023). Their functional role in some ecosystems is ~~such~~ sufficiently important that tortoises have become a model group in ecosystem restoration via rewilding, including through taxon substitution (Hansen *et al.* 2010; Griffiths *et al.* 2011; Svenning *et al.* 2016; Falcón & Hansen 2018; Tapia *et al.* 2022; Jones *et al.* 2023; Tapia Aguilera & Gibbs 2023). Understanding the ability of Testudines to respond to current environmental changes is therefore important to better preserve not only the turtles and tortoises themselves but also the functioning of the ecosystems they are part of.

Although some particularly iconic species draw a lot of public interest and have attracted an important amount of research (e.g., marine species), many species remain poorly known. This knowledge gap is likely to impact our ability to globally understand the ecological roles of turtles and tortoises, the capacity of this unique taxonomic group to respond to current biodiversity and climate crises, and our ability to preserve this group. Thanks to the efforts of multiple researchers, and especially of the Turtle Taxonomy Working Group (Rhodin *et al.* 2021; Rhodin *et al.* 2025), the current knowledge of Testudines species has been assembled, and the risk of extinction of 70~~69~~% of the 359 ~~357~~ currently recognized extant turtle species has now been assessed (IUCN 2025). However, the conservation status of the remaining 30~~1~~% (110 species) remains unclear. This reflects the existence of important discrepancies in the amount of research that has been dedicated to the different species of Testudines.

Formatted: English (United States)

Knowledge gaps and discrepancies in research effort can affect our understanding of the ecology, evolution, and conservation of Testudines for several reasons. First, they lead to the exclusion of the least studied species from reviews and comparative analyses, making the conclusions of these global studies less representative and more difficult to interpret. Second, they can affect trait estimates, e.g., because more extreme values are likely to be recorded in more investigated species. Third, they can alter the accuracy of species trait value estimates. Such values may be biased towards one or few individuals or populations in understudied species, but more representative of the entire species in more studied species. Finally, these knowledge gaps directly affect researchers and experts' ability to assess the conservation status of understudied species, excluding them *de facto* from conservation programs.

Such research gaps are common across taxonomic groups and have been identified across a diversity of taxa. Predictors of taxonomic variation in research effort have for example been investigated in birds (Ducatez & Lefebvre 2014), mammals (Santos *et al.* 2020; de Figueiredo

Machado *et al.* 2023), fishes (Ducatez 2019; Guy *et al.* 2021), a~~A~~mphibians (Silva *et al.* 2020) and plants (Adamo *et al.* 2021). Recently, a global assessment of variation in research effort across reptile species was conducted (Guedes *et al.* 2023), revealing important variation across reptile groups, and identifying biological and socioeconomic factors that determine how often a given species is studied. Such broad scale comparative analyses are particularly useful to identify factors determining discrepancies in research effort across species, and help mitigating these. Guedes *et al.* (2023) also brought to light an important fact about Testudines: although this class emerges as one of the most studied on average among reptiles, it also shows extreme variation within the group, with families such as the Cheloniidae having attracted the most attention while others such as Chelydridae were much less studied (Guedes *et al.* 2023). These observations call for a more detailed investigation of variation in research effort across Testudines species, an aspect that was not investigated in this previous work.

The objective of this study is to provide such investigation. On the one side, we aimed to identify which species and families are particularly studied, and which ones are particularly neglected, and at deciphering whether this relates to the ecology, life history and distribution of these species. For example, are aquatic turtles more often studied than terrestrial tortoises? Are species with a fast ~~lifestyle~~ life history (early reproduction and shorter lifespan) more studied than species with a slow ~~lifestyle~~ life history (late age at first reproduction, high longevity)? How does research effort vary across biogeographic realms in Testudines? Is research effort targeting species that have a higher risk of extinction? By clearly identifying understudied species, we hope to motivate the funding of research programs aiming at filling these knowledge gaps. In parallel, we aimed ~~at~~ to determine~~ing~~ how research effort variation may affect our global understanding of the ecology, evolution and conservation of turtles and tortoises. Literature reviews are necessary to understand global patterns and mechanisms within entire taxonomic groups such as the Testudines. However, they are also limited by the available literature and data: if research is taxonomically biased, the conclusions drawn from these presumably global reviews are likely to also be taxon-specific, making it important to identify these biases. Differences in research effort may also have direct consequences for some trait estimates. For example, turtles and tortoises are famous for their long life expectancy, with records in some species exceeding human lifetimes (> 100 years in several giant tortoise species, Bonin *et al.* 2006). Record longevities are however more likely to be detected in intensively studied species, as compared to less studied ones. ~~These knowledge gaps are particularly important to identify as the number of review articles on this taxonomic group has been increasing significantly over the last decades (see Figure 1).~~ Progress in our knowledge of Testudines phylogeny (there is now a tree based on genetic data from 80% of all turtle species: Thomson *et al.* 2021), together with advances in statistical analyses now allow researchers to run large scale analyses considering most species in this order and to bring a more global understanding of the importance of this group, but also of the threats it is facing. Identifying knowledge gaps should help taking them into account when designing these global analyses and interpreting their results.

Here, we first provide an index of research effort for each extant Testudines species, available for use in comparative studies. We tested for its robustness by comparing it with other indices. Second, we investigated how spread out this ~~index~~ research effort is, and tested whether research discrepancies improved or, on the contrary, became more extreme across time. Third, we investigated whether variation in research effort was related to taxonomy and phylogeny, ~~and~~ or to 14~~3~~ ~~traits~~ variables describing the distribution range, ecology, and life history of turtles. Finally, we tested whether research effort was predicted by extinction risk and population trend.

Methods

Research effort estimation

We used the taxonomy from the Turtle Taxonomy Working Group 2025~~1~~ (Rhodin *et al.* 2025), except for the Galapagos tortoises (*Chelonoidis sp.*) for which we followed the taxonomy considered by the IUCN, which recognizes 12 species that are considered as 12 subspecies of *C. niger* by ~~(~~Rhodin *et al.* (2025). This allowed to include this important taxon in the analyses considering extinction risk (the IUCN provides 12 different assessments for this set of 12 taxa) and resulted in a total of 370~~63~~ extant species.

Formatted: English (United States)

Formatted: English (United States)

We estimated the research effort devoted to each one of these 370~~63~~ species by extracting the number of references listed in the Web of Science (WoS) database (extraction conducted in January 2024). For each species, we conducted a search on WoS for papers published between 1975 and 2023, using the current species scientific name (e.g., "*Gopherus morafkai*") as keyword, and considering *all fields* (not just the title or keywords). We then extracted the number of articles obtained in the search as the index of research effort. In parallel, we conducted the same search but considering all synonyms of each species as keywords using the synonyms listed in ~~(~~Rhodin *et al.* (2025) (e.g., "*Gopherus morafkai*" OR "*Xerobates morafkai*"), to determine how name changes affected the research effort estimates. Progress in taxonomy can lead to multiple changes in species names, so that only including the current name may underestimate the research effort for some species (Correia *et al.* 2018). We also ran these two searches again but focusing on titles only, to compare an index of research effort based on the number of papers that are most likely to be focused on the focal species (as its name is in the title) with a more inclusive index that also considers articles including the focal species name in the abstract or keywords. Even in an article focused on a given species, the name of the species may not appear in its title, but in the keywords or abstract. In contrast, a species name may appear in the abstract or keywords of an article even if the article is not focused on that species. Comparing the research effort considering titles only vs all fields ~~will~~ should allow us to determine~~inform on~~ whether changing the search method ~~provides~~ affects the resulting ~~comparable~~ indices. Note that these indices are proxies for research effort, and not absolute estimates. They, for example, ignore the work that was published before 1975 as well as any reference that is not referenced in the WoS, including references from the grey literature. However, by constructing these indices, our purpose was to obtain a relative metric of research effort to compare species with each other, rather than an exact number of articles published per species. To test whether using different databases could affect this relative research effort score, we also compared the index obtained using the WoS database with the index extracted by Guedes *et al.* (2023) using Scopus and available for 343 of the 370~~63~~ species considered.

Variation in research effort distribution across time

To assess variation in research effort across time and species, we extracted the number of papers referenced in the Wo~~O~~S database for each species and decade (i.e., from 1980 to 1989, from 1990 to 1999, from 2000 to 2009, and from 2010 to 2019). For this approach, we included all fields (not just the title)~~,~~ and considered all synonyms of a given species name as keywords to be more inclusive and to account for name changes across time.

Trait collection

We extracted values for 14~~3~~ ~~traits~~ variables likely to either affect research effort or be affected by research effort: biogeographic realm, country research rank, range size, insularity, habitat, successful introduction, diet, carapace length, maximum longevity, generation length, female age at maturity, clutch size, incubation time and egg length. We extracted information for each species on biogeographic realm (ten different regions and one category for species occupying two or more regions), range size (both ~~presumed historic range size~~the estimated indigenous (historical) range (AOO) ~~-~~from ~~(~~Rhodin *et al.* (2025)~~-~~and current range size available in Guedes *et al.* (2023)), habitat (marine, terrestrial, freshwater or mixed for species occurring in both terrestrial and freshwater habitats), insularity (island or continent) and diet (herbivorous, carnivorous or omnivorous) from the IUCN red list website (IUCN 2025)~~-website~~, Rhodin *et al.* (2021), ~~(~~Rhodin *et al.* (2025) and Bonin *et al.* (2006). Information on ~~the~~ whether successful introductions of each species have occurred was also available in ~~(~~Rhodin *et al.* (2025). Country research rank was scored for each species based on the species distribution and Scimago country ranking in November 2025 (https://www.scimagojr.com/countryrank.php). The Scimago country rank is based on the number of articles published per country between 1996 and 2024. Species present in at least one of the top 10 countries were given a score of 3, ~~while other species were either given~~ a score of 2 if present in at least one of the top 10-20 countries, or a score of 1 ~~otherwise~~if not present in any of the top 20 research producing countries. We considered the maximum ~~standard~~ straight-line carapace length (in cm) as body size metric, available for most species from ~~(~~Rhodin *et al.* (2021; ~~Rhodin *et al.*~~ 2025). In addition, we retrieved information on maximum longevity, generation length, female age at maturity, clutch size, incubation time and egg length from https://www.demogr.mpg.de/longevityrecords/0403.htm, ~~(~~Miller *et al.* (2001)~~;~~ Bonin *et al.* (2006)~~;~~ Myhrvold *et al.* (2015), ~~;~~ Rodríguez-Caro *et al.* (2023). ~~-~~We extracted the risk of extinction and population trend of each assessed species from the IUCN's web site (IUCN 2025) in ~~December~~ November 202~~5~~3.

Analyses

We ran all the analyses using R (R Core Team 2024). To compare the different indices of research effort, we used Pearson correlation tests and coefficients on each pair of log-transformed indices (considering log (research effort +1)). To characterize the level of aggregation of research effort, we used the Poulin index of discrepancy (D, Poulin 1993). It allowed us to measure the deviation of research effort distribution from a theoretically even distribution among species. D ranges from zero to one, indicating no aggregation (0) to highly aggregated (1) distribution. This index was originally developed to quantify parasite aggregation across individuals in a host population. When investigating changes in D across decades, we excluded the species that had not yet been described in 1980. Research can only be dedicated to a given species after its description, and we aimed at using the same set of species to make the comparison meaningful. However, for the global estimate of discrepancy, we included all species. We used the QPweb software to calculate D (Reiczigel *et al.* 2019).

Phylogeny and t~~T~~axonomy

We built a Phylogenetic Linear Mixed Model (PLMM) using the function MCMCglmm from the R package *MCMCglmm* (Hadfield 2010) to estimate the proportion of variance in research effort (log-transformed) explained by phylogeny. We included phylogeny as random effect in a model with research effort (log-transformed) as response variable, using the phylogeny from Thomson *et al.* (2021), which used nuclear DNA to infer the phylogenetic relationships of 80% of the recognized extant Testudines species. This analysis was therefore conducted on a subset of 275 species included in this phylogeny. We used a poorly informative inverse Wishart prior (V = 1, ν = 0.002) for the

Formatted: English (United States)
Formatted: English (United States)
Formatted: French (France)
Formatted: French (France)
Field Code Changed
Field Code Changed
Formatted: French (France)
Formatted: French (France)
Field Code Changed
Formatted: French (France)
Formatted: French (France)
Field Code Changed
Formatted: English (United States)
Formatted: English (United States)
Formatted: English (United States)
Field Code Changed
Field Code Changed
Formatted: English (United States)

variances, following Hadfield (2010), and ran the model for 550001 iterations with a burn-in interval of 50000 to ensure satisfactory convergence, sampling one every 500 iterations, and checking that autocorrelation levels among samples was lower than 0.1. We assessed chain convergence by visually analyzing trace plots and running Gelman-Rubin convergence diagnostic on five chains (*gelman.diag* function from the package *coda*, Plummer *et al.* 2006). We calculated the intra-class coefficient (ICC) using the variance estimates of the model (with ICC = Vp / (Vp + Vr), where Vp = phylogenetic variance and Vr = residual variance).

To determine the proportion of variance in research effort explained by taxonomy, we built linear mixed models (LMM) with research effort (log + 1 transformed) as response variable, and either family or genus nested into family as random effects, using the *lme* function in the R package nlme (Pinheiro *et al.* 2023). We also built a null model (using the gls function) with no random effect and compared the AIC of these three models. We calculated the intra-class coefficient (ICC) of each taxonomic level using the variance estimates provided by the full model for both the family and genus effects.

Predictors of research effort

We investigated the association between research effort and 14~~3~~ species characteristics, including biogeographic range, country research rank, range size (considering either the ~~presumed historic range or current~~ estimated indigenous (historical) range ~~range~~or current range size), insularity (binary variable ~~segregating~~ separating island from continent species), habitat (factor with four levels: marine, freshwater, terrestrial or both freshwater and terrestrial), introduction (binary variable segregating successfully introduced species from other species), diet (herbivorous, carnivorous, omnivorous), maximum ~~standard~~ straight-line carapace length (SCL), maximum longevity, generation length, female age at maturity, clutch size, egg length and incubation time. We also ran an extra analysis focusing on either freshwater or terrestrial species to test whether the research effort varied between freshwater species occurring in rivers, still waterbodies or both rivers and still waterbodies, and between terrestrial species occurring in forests, open habitats, or both. We first tested for direct associations between research effort and each trait independently using Spearman rank correlations for continuous or ordinal variables (as some variables were not normally distributed), Wilcoxon tests for binary variables, or building linear models with research effort (log-transformed) as response variable and the factor of interest as explanatory variable for categorical variables (biogeographic range, habitat, and diet). This set of analyses allowed to maximize the sample size, as all the species with available information for the trait of interest could be included.

We then tested whether these associations were consistent when controlling for phylogeny (or whether phylogenetic non-independence alone could explain these associations), building one LMM per predictor with research effort (log-transformed) as response variable, the predictor of interest as explanatory variable and phylogeny as random effect. This analysis restricted the dataset to the 275 species included in the phylogeny. For categorical variables, we compared the deviance information criterion (DIC) of the model including vs excluding the categorical variable of interest to assess whether it was associated with research effort. The DIC is a variation of the AIC used in Bayesian model selection using MCMC simulations, and lower DIC indicate models with a better fit. Finally, we included all the predictors as fixed effects into one LMM with phylogeny as random effect to determine which predictors of research effort were the most important. We used a stepwise selection, removing non-significant predictors one by one until all predictors were significant (pMCMC < 0.05). We also used DIC comparisons of models including vs excluding each categorical variable to decide whether to include them or not in the final model (as MCMCglmm does not provide unique pMCMC values for categorical variables). Once a final model was selected, we added each removed parameter one by one to the model to validate it, determining whether previously

removed variables were still non-significant (or resulted in models with a higher DIC, using a threshold of ΔDIC = 5). For this analysis, we scaled all continuous predictors to a mean of 0 and a variance of 1 to obtain comparable estimates. This final global model could only include the species with trait values available for all the traits considered, and therefore was conducted on a significantly decreased sample size. Female age at maturity and generation length strongly decreased the number of species on which to run this analysis, and were never significantly associated with research effort, so we excluded them from this last analysis to maximize the sample size~~ (n = 107 species)~~. The correlation between predictors was always $<$ 0.4 except between maximum SCL and clutch size (Spearman ρ = 0.568, p < 0.001). To avoid collinearity issues, we excluded clutch size from this analysis~~7~~. With regards to range size, we considered the estimated indigenous range size (AOO), available for all species in our dataset, since current range size was only available for 313 of the 370 species (the two metrics were highly correlated: Spearman ρ = 0.931). The dataset for this multivariate analysis considered 115 species for which all 11 traits were available. We reran this model selection after excluding the sea turtles to test for result consistency.~~ Because sea turtles were clear outliers in terms of research effort, we excluded them from this analysis.~~

Extinction risk

We tested whether the research effort ~~of~~ for IUCN-assessed species differed from the non-assessed ~~ones~~species. Then, we tested whether extinction risk was associated with research effort, ranking the five IUCN extinction risk categories from 1 (critically endangered) to 5 (least concern) and excluding data deficient, extinct and non-assessed species (IUCN 2025). We also tested whether threatened (CR, EN or VU) and non-threatened (NT or LC) species had different research efforts, and whether population trend (with 0 = decreasing, 1 = stable or 2 = increasing, available from ~~(~~IUCN (2025) predicted research effort. To that aim, we built linear models with research effort (log-transformed) as response variable and either IUCN assessment (assessed or not), extinction risk, threatened (yes/no) or population trend as fixed effects. Considering extinction risk and population trend as continuous or as categorical variables resulted in the same conclusions so we only present the results considering them as continuous variables.

Results

Estimating research effort using species names in titles only or in titles, abstracts and keywords provided highly correlated coefficients (Pearson correlation coefficient between the log-transformed indices = 0.966~~7~~; p < 0.001). Similarly, considering synonyms or focusing on current names resulted in highly correlated coefficients (Pearson = 0.973~~67~~; p < 0.001). The research effort extracted from Scopus by Guedes *et al.* (2023) was also highly correlated with the indices obtained with references listed in the WoS (e.g., when considering all fields and synonyms, Pearson = 0.953~~40~~; p < 0.001; n = 342~~3~~ species). In the following analyses, we used the index considering species synonyms and all fields (not just the title) in the search for papers on WoS.

The search resulted in a total of 24442~~36~~ articles on the Web of Science. The mean number of articles per species was 66~~7~~ (SE = 16), and the median was six articles (range = 0 to 3441), suggesting that the distribution was highly right-skewed. The Poulin index of discrepancy confirmed that the distribution of research effort was highly aggregated (D = 0.873~~1~~, CI = [0.835~~27~~; 0.912~~04~~]). Although there was a small decrease in D across time (Poulin D; 1980s: 0.904~~2~~, CI = [0.877~~7~~ to 0.932~~30~~]; 1990s: 0.905~~4~~, CI = [0.869~~69~~; 0.929~~29~~]; 2000s: 0.867~~65~~, CI = [0.830~~28~~; 0.900~~896~~]; 2010s: 0.860~~59~~, CI = [0.809~~10~~ to 0.899~~900~~]), the change in discrepancy across decades was minimal (from 0.904~~2~~ to 0.860~~59~~).

The green sea-turtle (*Chelonia mydas*, with 3441 articles) and the loggerhead sea-turtle (*Caretta caretta*, with 3334 articles) were the two species with the highest index of research effort, followed by the pond slider (*Trachemys scripta*, with 2157 articles), painted turtle (*Chrysemys picta*, with 1445 articles) and common snapping turtle (*Chelydra serpentina*, with 1082 articles). The next three species were all marine turtles, so that out of the seven extant species of marine turtles, five are among the eight most studied species of Testudines. Sea turtles represent 1.9% of all Testudines species but gathered 39% of all publications in this group, with the least studied sea turtle (*Natator depressus*) obtaining a research effort score of 105 articles, 17 times higher than the median score for all Testudines. The most studied terrestrial species was the desert tortoise (*Gopherus agassizii*, ranking 11th with 454 articles). Twenty-nineThirty-two species had a score of 0, including species such as the enigmatic leaf turtle *Cyclemys enigmatica*, while the 50% least studied species gathered only 2% of the global research effort (47584 articles for 1851 species). Across decades, the number of articles published on turtles increased sharply: from 106784 in the 1980s to 9050 9766 in the 2010s, i.e. 9.21 times more in the 2010s than in the 1980s (Figure 12). This increase was particularly important in marine turtles, which attracted 18.1 times more research in the 1980s as compared to the 2010s (see Figure 1). 2).

Taxonomic and phylogenetic variation

The model including genus nested in family (AIC = 1329287.5) had a better fit than the one including family only (AIC = 134608) or the null model (AIC = 1402361). Together, family and genus explained 58% of the variance in research effort (ICC = 0.49 for family, ICC = 0.09 for genus). Phylogeny accounted for 66% of the variance when including it as a random effect in a PGLMM considering the 275 species of Testudines included in the phylogeny (extracting the ICC for family and genus with this same subset of 275 species resulted in an ICC of 0.48 for family and 0.09 for genus – thus 57% in total).

More diverse families also attracted more research (the number of papers per family increased significantly with the number of species per family, Figure 23). However, species diversity within a given family was not significantly correlated with the mean research effort per species (Figure 23). Some families were particularly often investigated particularly frequently, especially the two sea turtle families, while others were particularly neglected, such as the Pelomedusidae, a family of African freshwater turtles with five articles per species on average (Tsee table 1). Important variation in research effort across species within a given familyies werewas also noticeable (see Figure 3).

Biogeographic, ecological and life history predictors of research effort

Range size was significantly correlated with research effort;, species with a larger range attractededing more research (using the estimated indigenous (historical) rangepresumed historic range, n = 356370, Spearman ρ = 0.34740, p < 0.001; using the current range, n = 31306, Spearman ρ = 0.442, p < 0.001). In addition, species that have been successfully introduced outside of their native range had a higher research effort than the other species (Wilcoxon W = 38914701.5, p < 0.001; mean research effort = 1506..92 ± 48.550.1 articles for the 586 introduced species, 510.51 ± 16.223.0 for the 31020 non-introduced species). Research effort also varied significantly across geographic realms (Sum sq = 250.1839.61, F = 14.343.77, Df = 9, p < 0.001). In addition to species occupying multiple realms (mostly species occurring in both Central and North America, or marine species with broad

distribution), species from Europe and North America were given more research whereas species from the Caribbean, Central America or Africa were much less studied (see Figure 434). In addition, research effort varied significantly with -the research rank of countries where species occur (n = 370, Spearman ρ = 0.436, p < 0.001): research effort was highest for species occurring in at least one of the top 10 countries (n = 152, mean = 145, SE = 37), intermediate for species occurring in at least one of the top 10-20 (but none of the top 10) countries (n = 53, mean = 25, SE = 4), and lowest for species absent from the top 20 countries (n = 165, mean = 9, SE = 1).

Research effort did not vary significantly between island and continental species (n = 370, Wilcoxon W = 607655523, p = 0.082141), but habitat was a significant predictor of research effort (n = 352, Sum sq = 140.8198, F = 21.91713, Df = 3, p < 0.001; Figure 545), with marine species being more often studied more frequently than species from any other habitat category (all p < 0.001). Research effort did not differ significantly between freshwater, terrestrial and mixed-habitat species (all p > 0.1386). Focusing on either freshwater or terrestrial species, the type of freshwater or terrestrial habitat did not significantly predict variation in research effort (freshwater: n = 126, Sum sq = 5.365, F = 1.243, Df = 2, p = 0.292; terrestrial: n = 54, Sum sq = 5.972, F = 1.573, Df = 2, p = 0.217).

Research effort did not vary across diet categories either (n = 257, Sum sq = 0.89, F = 0.174, Df = 2, p = 0.841). Considering life history traits, research effort increased significantly with body size (maximum SCL: n = 369, Spearman ρ = 0.210199, p < 0.001), maximum longevity (n = 202, Spearman ρ = 0.339, p < 0.001) and clutch size (n = 275, Spearman ρ = 0.148, p = 0.014), decreased significantly with egg size (n = 189, Spearman ρ = -0.161189, p = 0.02610) but was not significantly correlated with generation length (n = 139, Spearman ρ = 0.017, p = 0.839), female age at maturity (n = 92, Spearman ρ = 0.151, p = 0.150) or incubation time (n = 212, Spearman ρ = -0.127116, p = 0.06592).

Building separate phylogenetic mixed models for each of these 143 predictors led to similar conclusions, except for the insularity effect which became significant – insular species had a significantly lower research effort than continental ones (pMCMC = 0.0026) – and for incubation time as research effort decreased significantly with incubation time (pMCMC = 0.028) once correcting for phylogenetic non-independence (Table 2).

The best model with multiple predictors included seven five different predictors, in addition to the random effect of phylogeny: range size, introduction status, biogeographic realm, maximum SCL, maximum longevity, clutch size and egg length. The direction of their effects was similar to those detected using univariate models, except for clutch size, which effect became negative (see Table 3, all pMCMC < 0.002, or ΔDIC = 14.6). Note that adding habitat to this model resulted in a very similar DIC (ΔDIC = 0.4). Excluding marine species resulted in the same best model and qualitatively similar results (see Suppl. Mat. 1).

Formatted: Font: Not Italic

Extinction risk

IUCN-assessed species had a higher research effort than non-assessed or data-deficient species (estimate = 0.904749 ± 0.17678, t = 5.1234.201, p < 0.001; mean number of papers for assessed species = 86.29.7 ± 22.28; for non-assessed species = 14.85.9 ± 2.67). The research effort of threatened and non-threatened species did not differ significantly (estimate = -0.0990.010 ± 0.214220, t = -0.4630.045, p = 0.644964), and the risk of extinction did not significantly predict research effort (n = 160, estimate = -0.104062 ± 0.0701, t = 1.486-0.877, p = 0.138381). Similarly, research effort did not vary significantly with population trend (n = 177, estimate = 0.114078 ± 0.23459, t = 0.488302, p = 0.626763).

Discussion

Identifying taxonomic biases in research effort can help future research programs to aim at correcting these biases and improve our global understanding of turtles and tortoises. We underline here that Testudines show strong discrepancies in research effort across species, with the overwhelmingly high proportion of research dedicated to a small number of sea turtle species being particularly striking. Phylogeny itself explained 66% of the variance in research effort, also illustrating that some parts of the Testudines tree have been much more researched than others. Although overall, the number of publications focused on Testudines has increased substantially over the last decades, discrepancies across species have been maintained so that understudied species remained understudied while more studied species keep attracting more research. Our results also underline that these research-biases are not neutral with regards to species traits – instead, more research is dedicated towards species that share similar biogeographic, ecological and life history traits. Neither high risk nor low risk species were particularly targeted by researchers since conservation status did not predict research effort.

Our analyses underline the high and non-neutral (with regards to species traits) heterogeneity in research effort across turtle species. This heterogeneity showed ~~little~~ few changes over the last decades, as illustrated by the minimal decrease in research load discrepancy between the 1980s and 2010s. This is not unique to turtles, and has been described in other taxonomic groups, including birds (Ducatez & Lefebvre 2014), mammals (de Figueiredo Machado *et al.* 2023), fishes (Ducatez 2019; Guy *et al.* 2021) and amphibians (Silva *et al.* 2020). Although our result suggests a need for a reorientation of research efforts and funding towards less studied species, different explanations can help in interpreting it. First, some species are used as model organisms, responding to the need of acquiring in-depth knowledge of some species to address more advanced biological questions, and leading to outlier-species which attracted the attention of many researchers. *Trachemys scri~~y~~pta* is one such example: the pond slider has been the focus of research addressing questions in a diversity of disciplines, including molecular biology (e.g., Milton *et al.* 2006), development biology (Lin *et al.* 2024), physiology (Overgaard *et al.* 2002), ecology (e.g., Lindsay *et al.* 2013) and cognition (e.g., Sun *et al.* 2023), although understanding the impacts of this species that is invasive in part of its range also contributed to its success with researchers (e.g., see Balzani *et al.* 2016). The relatively small size of this species, its adaptability to captivity and its distribution (in both its native and introduced range) in areas with high scientific capacity made it an ideal model species to study both in the lab and *in situ*.

Second, some species are the target of research because of their sociocultural importance. The focus on charismatic species in conservation has been seen as a way to attract funding and public attention towards conservation issues, although ~~it~~ this has been ~~largely~~ debated (Bowen-Jones & Entwistle 2002; Small 2012a; Small 2012b; Macdonald *et al.* 2015). From a global knowledge perspective, this focus on some flagship species targeted because of their charisma is more ~~concerning~~problematic, as it is likely to both concentrate the funding on a small subset of species, and lead to a lack of representativeness of the knowledge on a given taxonomic group such as the Testudines. For example, the particularly high amount of research dedicated to sea turtles is likely mostly explained by their cultural value (Kitolelei *et al.* 2022) and by the fascination that they represent for the public, increasing the probability of research funding attribution for this group of turtles. This can be interpreted as a positive outcome: our knowledge of sea turtles is particularly good, especially for species that are difficult to study given their marine lifestyle. In addition, the original habitat and migratory behaviors of sea turtles also increases the relevance of investigating these species.

Field Code Changed

Formatted: French (France)

Formatted: French (France)

Field Code Changed

However, the contrast with non-marine species ~~questions~~ highlights the way research resources are distributed: on average, a sea-turtle species received 33 times more research than a freshwater or terrestrial species. The endangerment of sea turtle species (~~six~~ five are considered at risk of extinction, one is data deficient and one is considered Least Concern) together with their unique use of the marine habitat might be seen as justifications for the high effort put towards sea turtles. However, this is not specific to sea turtles: only 24~~3~~% of all freshwater and terrestrial species are considered as least concern or near threatened, with the others ~~ones~~ being either at risk of extinction, not assessed or data deficient. The high proportion of freshwater and terrestrial species which risk of extinction has not been evaluated (109 species) is also a major concern, as under-studied species tend to have a higher risk of extinction than species that have been evaluated (Howard & Bickford 2014; Wotherspoon *et al.* 2024). Using sea-turtles as flagship species to attract public attention and funding towards other groups of turtles might be a strategy to fill some of these major knowledge gaps.

The ease of researcher~~s'~~ access to a given species is also an important factor determining species knowledge production. This is likely reflected in the biogeographic traits that determined research effort in our analyses. Species with a larger range are more likely to include research facilities within their range and are therefore more often investigated, while continental species are often more accessible than insular ones (although the insularity effect was only significant when considered as single predictor in a ~~G~~LMM). Similarly, species distributed in areas with a higher scientific capacity, such as North America and Europe or any of the top 10 or top 20 most scientifically productive countries, are more studied than species from areas with more limited research funding such as Africa or Central America. This result is in line with the literature: for example, ~~(~~Guedes *et al.* (2023) found that reptile species which range overlaps with biodiversity institutions received more research attention. Some of the most important turtle biodiversity hotspots are in areas that are relatively neglected by researchers (Australasia, Africa, South America; Bonin *et al.* 2006). Similar patterns were detected in other taxonomic groups, including in reptiles (Guedes *et al.* 2023), birds (Ducatez & Lefebvre 2014) or sharks (Ducatez 2019), underlining that these research biases are not unique to turtles and tortoises. Therefore, conclusions of large-scale studies are likely to mostly apply for well-studied regions such as North America and Europe, but less so to understudied areas such as Central America or Africa. Overall, although targeting species that are easier to access is meaningful from both a financial and a logistic perspective, orienting research towards understudied areas, localized and/or insular species to correct these biases is highly relevant for turtles as for a diversity of other taxonomic groups.

Several life history traits were associated with research effort. These associations might be the result of differences in how easy it is to study species with particular traits. For example, larger species ~~are~~ can be easier to detect and study, and often characterized by a longer lifespan, potentially explaining the positive association between research effort and longevity. Alternatively, for some traits, differences in research ~~pressure~~ effort are likely to directly affect the trait estimates. For example, record longevities are more likely to be documented in intensely studied species than in less studied ones, leading to biased differences in the estimated species maximum longevities. Associations between research effort and trait values due to biased estimates or trait-based selection of researched species are likely to have consequences for the interpretation of comparative analyses and literature reviews. Such studies are often meant to be representative of entire taxonomic groups. They however tend to exclude poorly known species and/or consider biased or imprecise trait estimates due to species knowledge discrepancies, making their conclusions less general than expected. Small turtle and tortoise species with small eggs, small clutches and short longevity are, for example, less well known, and therefore likely to be more often excluded from global analyses,

Formatted: Not Highlight

Formatted: English (United States)

Formatted: English (United States)

biasing ~~their~~ any results to a subset of species that is not representative of the entire group. In contrast, maternal age at reproduction and generation length were not associated with research effort. However, t~~T~~hese two traits were available for small numbers of species (110 and 86 species, see Table 2), and the lack of significant association might be due to a lack of statistical power. Overall, the importance of these knowledge differences in shaping the results of broad taxonomic scale analyses needs to be acknowledged in order to properly interpret their results.

Apart from the strong research bias towards marine species, ecological traits did not predict variation in research effort across species. When focusing on non-marine species, research effort did not vary according to Testudines habitats or diet. The lack of differences in research effort related to either habitat or diet is likely robust since these two traits were available for most species. It suggests that species with different habitats (with the exception of marine habitats) and diet have been relatively evenly investigated, contrasting with other taxonomic groups where species from some habitats tend to be understudied. For example, deep-sea shark species were less often investigated than species occurring closer to the surface (Ducatez 2019).

Previous works have evidenced the importance of other traits as predictors of variation in research effort across species. Socioeconomic factors, including range overlap with biodiversity institutions, were for example shown to increase the amount of research dedicated to reptile species (Guedes *et al.* 2023), while recently described species tend to have received less research (Guedes *et al.* 2023). Aesthetic biases have also been demonstrated for example in plants where more colourful and conspicuous species attracted more research (Adamo *et al.* 2021). The use of anthropic habitats also tends to increase research attention, including in amphibians (Silva *et al.* 2020). Such parameters ~~are likely to~~may also influence research effort in turtles and tortoises and could be considered in future studies.

The number of publications per species did not vary with either extinction risk or population trend across Testudines species. Removing sea turtles from the analyses did not change this result (~~unshown~~ additional results). Similarly, considering a categorical variable instead of a continuous one to represent either extinction risk or population trend led to the same conclusion. This result contrasts with several other taxonomic groups, where researchers tend to focus more on low risk species (e.g., in Amphibians, Silva *et al.* 2020; in birds, Ducatez & Lefebvre 2014) or species with intermediate risk levels (e.g., in sharks, Ducatez 2019). This relatively even distribution of research attention across extinction risk categories may be considered as a positive result, especially as compared to other taxa: endangered species are not relatively understudied. However, a special research focus on endangered species would be desirable to improve conservation practices on species that need it the most. ~~.~~ A study focusing on research effort on turtles and tortoises in conservation science (rather than all publications) and its distribution according to species risk of extinction would be of interest to better assess the needs for conservation research in endangered species (see for example this study on primates: de Figueiredo Machado *et al.* 2023). The (expected) much lower number of publications on the 110 species that were either not assessed or considered data deficient (as compared to species which extinction risk has been assessed) also raises important concerns, as these species tend to be at a higher risk of extinction according to work conducted on other taxonomic groups (Howard & Bickford 2014; Wotherspoon *et al.* 2024). Overall, species that are at high risk of extinction, as well as species which extinction risk has not been assessed yet, or which extinction risk could not be assessed because of data deficiency, should be the focus of more research to optimize our chances of implementing appropriate conservation actions for the entire clade. Future work investigating the association between research effort and conservation funding

Formatted: Not Highlight

per species would also provide valuable information on how indicative of conservation funding indices of research efforts can be.

Assessing the amount of research dedicated to a given species requires to make choices with regards to the sources and criteria considered. Including one or several databases, considering or not the grey literature, accounting for name changes or quantifying the amount of research dedicated to a given species within publications are among the important factors likely to affect conclusions. The index we are using cannot be directly interpreted as an absolute measure of the global research effort that was dedicated to a given species. Studies published before 1975 were not included in this index, and grey literature as well studies~~as any study~~ not included in the WoS were ~~ignored~~excluded when calculating this index. In addition, even when ignoring these limitations, the number of articles mentioning a species name in its title or abstract is a rough proxy for research effort: the amount of published research and knowledge provided on a given species can vary substantially from one article to another, and using the number of articles is an important shortcut. Other indices, including information on species knowledge (e.g., trait value knowledge, number of individuals measured, duration of studies) would provide complementary information on how much research was invested in a given species. However, these ~~decisions~~ limitations are mostly important when aiming at obtaining absolute indices able to capture all or most research that has been conducted on a given species. Here, our purpose was to obtain a metric that allows comparisons of research effort across species, i.e.~~that is to say~~, to evaluate the amount of research attributed to a given species relative to others. To that aim, we demonstrated that the choice of one index over another had little importance for Testudines, since all tested indices were highly correlated. Including synonyms or not, considering all fields or only the titles during the search for papers, and considering the Web of Science or Scopus databases resulted in comparable numbers of publications per species (all Pearson coefficients > 0.94) – a pattern similar to other taxonomic groups (Ducatez & Lefebvre 2014; Ducatez 2019). That being said, we recommend using the index that takes into account synonyms (see Correia *et al.* 2018) and all fields (rather than focusing on articles where the species names appears in the title), as it is more inclusive.

By identifying both the most and least studied families and species of Testudines, we hope that this work will help orienting research towards taxa and regions that require more research. Families such as the Pelomedusidae, with only five articles per species on average (~~see~~ Table 1), would, for example, warrant much more research. Although having model species is relevant and allows addressing fundamental questions more in depth, the lack of knowledge on a significant proportion of the Testudines is likely to affect our interpretation of their global ecological importance and our ability to preserve them. More research on endangered species, including in areas such as South East Asia that concentrate a high proportion of high risk or unassessed species (Mittermeier *et al.* 2015), but also to understudied areas such as Africa and Central America, would be especially rewarding by both increasing our knowledge of this unique taxonomic group and identifying actions that could help improv~~eing~~ the conservation status of endangered species. ~~Redirecting~~ Sharing funding allocated to sea turtle research and conservation might also be a way forward to fill the knowledge gaps that are likely to prevent effective conservation actions for terrestrial and freshwater species that also play key roles in their ecosystems. By providing an index of research effort for all species of Testudines, we also hope to help account for potential biases related to discrepancies in research effort across species in global reviews and comparative analyses.

References

ADAMO, M., CHIALVA, M., CALEVO, J., BERTONI, F., DIXON, K. & MAMMOLA, S. 2021. Plant scientists' research attention is skewed towards colourful, conspicuous and broadly distributed flowers. *Nature Plants* 7: 574–578. doi:10.1038/s41477-021-00912-2

Formatted: English (United States)

BALZANI, P., VIZZINI, S., SANTINI, G., *et al.* 2016. Stable isotope analysis of trophic niche in two co-occurring native and invasive terrapins, *Emys orbicularis* and *Trachemys scripta elegans*. *Biological Invasions* 18: 3611–3621. doi:10.1007/s10530-016-1251-x

Formatted: English (United States)

BONIN, F., DEVAUX, B. & DUPRÉ, A. 2006. *Turtles of the World*. New-York, USA: Johns Hopkins University Press.

Formatted: English (United States)

BOWEN-JONES, E. & ENTWISTLE, A. 2002. Identifying appropriate flagship species: the importance of culture and local contexts. *Oryx* 36: 189–195. doi:10.1017/S0030605302000261

CHEN, T.-H., CHANG, H.-C. & LUE, K.-Y. 2009. Unregulated trade in turtle shells for Chinese traditional medicine in East and Southeast Asia: the case of Taiwan. *Chelonian Conservation and Biology* 8: 11–18. doi:10.2744/CCB-0747.1

CHEUNG, S.M. & DUDGEON, D. 2006. Quantifying the Asian turtle crisis: market surveys in southern China, 2000–2003. *Aquatic Conservation: Marine and Freshwater Ecosystems* 16: 751–770. doi:10.1002/aqc.803

CHRISTIANEN, M.J.A., SMULDERS, F.O.H., VONK, J.A., *et al.* 2023. Seagrass ecosystem multifunctionality under the rise of a flagship marine megaherbivore. *Global Change Biology* 29: 215–230. doi:10.1111/gcb.16464

CORREIA, R.A., JARIĆ, I., JEPSON, P., MALHADO, A.C.M., ALVES, J.A. & LADLE, R.J. 2018. Nomenclature instability in species culturomic assessments: Why synonyms matter. *Ecological Indicators* 90: 74–78. doi:10.1016/j.ecolind.2018.02.059

DUCATEZ, S. 2019. Which sharks attract research? Analyses of the distribution of research effort in sharks reveal significant non-random knowledge biases. *Reviews in Fish Biology and Fisheries* 29: 355–367. doi:10.1007/s11160-019-09556-0

DUCATEZ, S. & LEFEBVRE, L. 2014. Patterns of research effort in birds. *PLoS ONE* 9: e89955. doi:10.1371/journal.pone.0089955

FALCÓN, W. & HANSEN, D.M. 2018. Island rewilding with giant tortoises in an era of climate change. *Philosophical Transactions of the Royal Society B: Biological Sciences* 373: 20170442. doi:10.1098/rstb.2017.0442

FALCÓN, W., MOLL, D. & HANSEN, D.M. 2020. Frugivory and seed dispersal by chelonians: a review and synthesis. *Biological Reviews* 95: 142–166. doi:10.1111/brv.12558

DE FIGUEIREDO MACHADO, F., ROCHA, B.S., BRITO, D. & TERRIBILE, L.C. 2023. Trends and biases in research efforts for primate conservation: threatened species are not in the spotlight. *Perspectives in Ecology and Conservation* 21: 286–293. doi:10.1016/j.pecon.2023.10.001

FROYD, C.A., COFFEY, E.E.D., VAN DER KNAAP, W.O., VAN LEEUWEN, J.F.N., TYE, A. & WILLIS, K.J. 2014. The ecological consequences of megafaunal loss: giant tortoises and wetland biodiversity. *Ecology Letters* 17: 144–154. doi:10.1111/ele.12203

GRIFFITHS, C.J., HANSEN, D.M., JONES, C.G., ZUËL, N. & HARRIS, S. 2011. Resurrecting extinct interactions with extant substitutes. *Current Biology* 21: 762–765. doi:10.1016/j.cub.2011.03.042

GUEDES, J.J.M., MOURA, M.R. & ALEXANDRE F. DINIZ-FILHO, J. 2023. Species out of sight: elucidating the determinants of research effort in global reptiles. *Ecography* 2023: e06491. doi:10.1111/ecog.06491

GUY, C.S., COX, T.L., WILLIAMS, J.R., *et al.* 2021. A paradoxical knowledge gap in science for critically endangered fishes and game fishes during the sixth mass extinction. *Scientific Reports* 11: 8447. doi:10.1038/s41598-021-87871-y

HADFIELD, J.D. 2010. MCMC Methods for Multi-Response Generalized Linear Mixed Models: The MCMCglmm R Package. *Journal of Statistical Software* 33: 1–22. doi:10.18637/jss.v033.i02

HANSEN, D.M., DONLAN, C.J., GRIFFITHS, C.J. & CAMPBELL, K.J. 2010. Ecological history and latent conservation potential: large and giant tortoises as a model for taxon substitutions. *ECOGRAPHY* 33: 272–284. doi:10.1111/j.1600-0587.2010.06305.x

HOWARD, S.D. & BICKFORD, D.P. 2014. Amphibians over the edge: silent extinction risk of Data Deficient species. *Diversity and Distributions* 20: 837–846. doi:10.1111/ddi.12218

IUCN 2025. The IUCN Red List of Threatened Species Version 2025-1.

JONES, C.G., TATAYAH, V., MOORHOUSE-GANN, R., GRIFFITHS, C., ZUEL, N. & COLE, N. 2023. *Slow and Steady Wins the Race: Using Non-native Tortoises to Rewild Islands off Mauritius*. M. J. Gaywood, J. G. Ewen, P. M. Hollingsworth, & A. Moehrenschlager (Eds.). Cambridge: Cambridge Univ Press.

KITOLELEI, S., SODERBERG, A., QAQARA, N., *et al.* 2022. Conservation status and cultural values of sea turtles leading to (un)written parallel management systems in Fiji. *Ambio* 51: 2431–2444. doi:10.1007/s13280-022-01766-4

LENTH, R. 2024. emmeans: Estimated marginal means, aka least-squares means. *R package version 1.10.5*.

LIN, J., ZHANG, M., LIANG, F., *et al.* 2024. Morphological and transcriptomic analyses of embryonic development of red-eared slider *Trachemys scripta elegans*. *Animal Reproduction Science* 261: 107395. doi:10.1016/j.anireprosci.2023.107395

LINDSAY, M.K., ZHANG, Y., FORSTNER, M.R.J. & HAHN, D. 2013. Effects of the freshwater turtle *Trachemys scripta elegans* on ecosystem functioning: an approach in experimental ponds. *Amphibia-Reptilia* 34: 75–84. doi:10.1163/15685381-00002871

LOVICH, J.E., ENNEN, J.R., AGHA, M. & GIBBONS, J.W. 2018. Where have all the turtles gone, and why does it matter? *BioScience* 68: 771–781. doi:10.1093/biosci/biy095

MACDONALD, E.A., BURNHAM, D., HINKS, A.E., DICKMAN, A.J., MALHI, Y. & MACDONALD, D.W. 2015. Conservation inequality and the charismatic cat: *Felis felicis*. *Global Ecology and Conservation* 3: 851–866. doi:10.1016/j.gecco.2015.04.006

MILLER, J.D., LIMPUS, C.J. & GODFREY, M.H. 2001. Nest Site Selection, Oviposition, Eggs, Development, Hatching, and Emergence of Loggerhead Turtles. Biology and conservation of loggerhead sea turtle. University of Florida Press

Formatted: English (United States)

Formatted: English (United States)

MILTON, S.L., NAYAK, G., LUTZ, P.L. & PRENTICE, H.M. 2006. Gene transcription of neuroglobin is upregulated by hypoxia and anoxia in the brain of the anoxia-tolerant turtle *Trachemys scripta*. *Journal of Biomedical Science* 13: 509–514. doi:10.1007/s11373-006-9084-8

MITTERMEIER, R.A., DIJK, P.P. van, RHODIN, A.G.J. & NASH, S.D. 2015. Turtle hotspots: an analysis of the occurrence of tortoises and freshwater turtles in biodiversity hotspots, high-biodiversity wilderness areas, and turtle priority areas. *Chelonian Conservation and Biology* 14: 2–10. doi:10.2744/ccab-14-01-2-10.1

MYHRVOLD, N.P., BALDRIDGE, E., CHAN, B., SIVAM, D., FREEMAN, D.L. & ERNEST, S.K.M. 2015. An amniote life-history database to perform comparative analyses with birds, mammals, and reptiles. *Ecology* 96: 3109–3109. doi:10.1890/15-0846R.1

OVERGAARD, J., STECYK, J.A.W., FARRELL, A.P. & WANG, T. 2002. Adrenergic control of the cardiovascular system in the turtle *Trachemys scripta*. *The Journal of Experimental Biology* 205: 3335–3345. doi:10.1242/jeb.205.21.3335

PINHEIRO, J., BATES, D. & R CORE TEAM 2023. nlme: Linear and Nonlinear Mixed Effects Models.

PLUMMER, M., BEST, N., COWLES, K. & VINES, K. 2006. CODA: Convergence diagnosis and output analysis for MCMC. *R News* 6: 7–11.

POULIN, R. 1993. The disparity between observed and uniform distributions: A new look at parasite aggregation. *International Journal for Parasitology* 23: 937–944. doi:10.1016/0020-7519(93)90060-C

R CORE TEAM 2024. R: A Language and Environment for Statistical Computing.

REICZIGEL, J., MAROZZI, M., FÁBIÁN, I. & RÓZSA, L. 2019. Biostatistics for parasitologists – a primer to quantitative parasitology. *Trends in Parasitology* 35: 277–281. doi:10.1016/j.pt.2019.01.003

RHODIN, A.G.J., IVERSON, J.B., BOUR, R., *et al.* 2021. Turtles of the World: Annotated checklist and atlas of taxonomy, synonymy, distribution, and conservation status (9th Ed.). *Chelonian Research Monographs* 8: 1–472. doi:10.11606/issn.2316-9079.v20i2p225-228

RHODIN, A.G.J., IVERSON, J.B., FRITZ, U., *et al.* 2025. Turtles of the World: Annotated checklist and atlas of taxonomy, synonymy, distribution, and conservation status (10th Ed.). *Chelonian Research Monographs* 10. doi:10.11606/issn.2316-9079.v20i2p225-228

RODRÍGUEZ-CARO, R.C., GRACIÁ, E., BLOMBERG, S.P., *et al.* 2023. Anthropogenic impacts on threatened species erode functional diversity in chelonians and crocodilians. *Nature Communications* 14: 1542. doi:10.1038/s41467-023-37089-5

SANTOS, J.W. dos, CORREIA, R.A., MALHADO, A.C.M., *et al.* 2020. Drivers of taxonomic bias in conservation research: a global analysis of terrestrial mammals. *Animal Conservation* 23: 679–688. doi:10.1111/acv.12586

SEMINOFF, J.A. 2025. The global turtle crisis—a wildlife trafficking challenge like no other. *Chelonian Conservation and Biology* 23: 135–136. doi:10.2744/1071-8443-23.2.1

SHEARER, I. & TÜRKOZAN, O. 2025. Global *Testudo* trade: update and recent trends. *Chelonian Conservation and Biology* 23: 161–168. doi:10.2744/CCB-1643.1

Formatted: English (United States)

SILVA, A.F. da, MALHADO, A.C.M., CORREIA, R.A., LADLE, R.J., VITAL, M.V.C. & MOTT, T. 2020. Taxonomic bias in amphibian research: Are researchers responding to conservation need? *Journal for Nature Conservation* 56: 125829. doi:10.1016/j.jnc.2020.125829

SMALL, E. 2012a. The new Noah’s Ark: beautiful and useful species only. Part 1. Biodiversity conservation issues and priorities. *Biodiversity* 12: 232–247. doi:10.1080/14888386.2011.642663

SMALL, E. 2012b. The new Noah’s Ark: beautiful and useful species only. Part 2. The chosen species. *Biodiversity* 13: 37–53. doi:10.1080/14888386.2012.659443

STANFORD, C.B., IVERSON, J.B., RHODIN, A.G.J., *et al.* 2020. Turtles and tortoises are in trouble. *Current Biology* 30: R721–R735. doi:10.1016/j.cub.2020.04.088

SUN, X., PIAO, Y., WANG, T., WANG, J., FU, J. & CUI, J. 2023. Keep numbers in view: red-eared sliders (*Trachemys scripta elegans*) learn to discriminate relative quantities. *Biology Letters* 19: 20230203. doi:10.1098/rsbl.2023.0203

SVENNING, J.-C., PEDERSEN, P.B.M., DONLAN, C.J., *et al.* 2016. Science for a wilder Anthropocene: Synthesis and future directions for trophic rewilding research. *Proceedings of the National Academy of Sciences* 113: 898–906. doi:10.1073/pnas.1502556112

TAPIA AGUILERA, W. & GIBBS, J.P. 2023. Rewilding giant tortoises engineers plant communities at local to landscape scales. *Conservation Letters* 16: e12968. doi:10.1111/conl.12968

TAPIA, W., GOLDSPIEL, H.B. & GIBBS, J.P. 2022. Introduction of giant tortoises as a replacement “ecosystem engineer” to facilitate restoration of Santa Fe Island, Galapagos. *Restoration Ecology* 30: e13476. doi:10.1111/rec.13476

THOMSON, R.C., SPINKS, P.Q. & SHAFFER, H.B. 2021. A global phylogeny of turtles reveals a burst of climate-associated diversification on continental margins. *Proceedings of the National Academy of Sciences* 118: e2012215118. doi:10.1073/pnas.2012215118

WOTHERSPOON, L., DE OLIVEIRA CAETANO, G.H., ROLL, U., *et al.* 2024. Inferring the extinction risk of Data Deficient and Not Evaluated Australian squamates. *Austral Ecology* 49: e13485. doi:10.1111/aec.13485

Table 1. Species richness, mean research effort per species and total research effort per family in Testudines.

	Number of species	Mean number of papers	Total number of papers	Standard error
CHELONIIDAE	6	1429	8575	628
DERMOCHELYIDAE	1	1062	1062	
CHELYDRIDAE	5	243	1213	211
EMYDIDAE	58	111	6410	45
CARETTOCHELYIDAE	1	54	54	
PODOCNEMIDIDAE	8	50	402	21
PLATYSTERNIDAE	1	50	50	
TESTUDINIDAE	58	50	2874	13
TRIONYCHIDAE	35	32	1119	17
DERMATEMYDIDAE	1	29	29	
KINOSTERNIDAE	34	16	548	6
GEOEMYDIDAE	75	15	1138	3
CHELIDAE	60	14	833	3
PELOMEDUSIDAE	27	5	135	2

Formatted: English (United States)

Table 2. Association between research effort and 14~~3~~ predictors in Testudinidae, after taking phylogenetic relationships into account. Research effort was included as response variable in 14~~3~~ different PLMM that included phylogeny as random effect and either one of the 14~~3~~ predictors as fixed effects. For continuous or binary predictors, pMCMC was used to assess significance (a). A positive effect of introduction means that species that were introduced successfully had a higher research effort; a negative effect of insularity means that insular species had a lower research effort than continental ones. For categorical variables, DIC-based comparisons of models including or excluding each categorical predictor are shown (b) as MCMCglmm does not provide unique pMCMC values for these variables (a negative ΔDIC indicates that the predictor improved the fit of the model). SCL = ~~Standard~~ Straight-line Carapace Length; pm = posterior mean; CI = credibility interval, eff samp = effective sample size, n = number of species included. Significant effects are in bold style.

Binary or continuous variables	pm	CI	eff samp	pMCMC	n
**Historic range size (AOO)**	**0.757**	**[0.631; 0.929]**	**1001**	**<0.001**	**275**
**Current range size**	**0.864**	**[0.705; 1.005]**	**1001**	**<0.001**	**251**
**Introduction**	**1.161**	**[0.748; 1.570]**	**1001**	**<0.001**	**275**
**Insularity**	**-1.144**	**[-1.840; -0.447]**	**1001**	**0.002**	**275**
**Country research rank**	**0.731**	**[0.548; 0.918]**	**1001**	**<0.001**	**275**
**Max SCL**	**0.015**	**[0.005; 0.024]**	**1001**	**0.008**	**274**
**Max longevity**	**0.018**	**[0.009; 0.027]**	**909.6**	**<0.001**	**185**
Generation length	-0.007	[-0.033; 0.018]	1001	0.601	110
Maternal age at maturity	0.026	[-0.312; 0.440]	1190	0.897	85
**Clutch size**	**0.028**	**[0.012; 0.042]**	**1001**	**<0.001**	**246**
**Egg length**	**-0.028**	**[-0.054; -0.003]**	**1001**	**0.028**	**172**
**Incubation time**	**-0.006**	**[-0.011; -0.001]**	**1001**	**0.028**	**194**

Categorical variables	DIC with	DIC without	ΔDIC	n
**Biogeographic realm**	**909**	**949**	**-40**	**275**
Habitat	950	944	6	273
Diet	786	783	3	229

Table 3. Best multi-predictor model explaining variation in research effort across species of Testudinidae. Phylogeny was included as random effect, and we used the MCMCglmm procedure. Non-significant predictors excluded from the best model (pMCMC > 0.01 or ΔDIC > 5) are shown in italic style below the factors included in the best model. -SCL = ~~Standard~~ Straight-line Carapace Length; pm = posterior mean; CI = credibility interval, eff.samp = effective sample size. 115 species were included in this analysis.

Explanatory variable	pm	95% CI	eff.samp	pMCMC
Intercept	2.269	[0.474; 4.226]	1125.8	0.018
Historic range size	0.668	[0.408; 0.941]	1205.5	<0.001
Introduction	0.463	[0.025; 0.905]	1001	0.042
Maximum SCL	0.854	[0.470; 1.184]	900.8	<0.001
Egg length	-0.433	[-0.734; -0.133]	1001	0.004
Biogeographic realm (ΔDIC = -32)				
Maximum longevity	*0.184*	*[-0.059; 0.396]*	*1001*	*0.118*
Insularity	*0.309*	*[-1.240; 1.734]*	*908.9*	*0.691*
Incubation	*-0.029*	*[-0.331; 0.211]*	*1001*	*0.859*
Country research rank	*0.035*	*[-0.318; 0.330]*	*1001*	*0.839*
Diet (ΔDIC = 8)				
Habitat (ΔDIC = 27)				

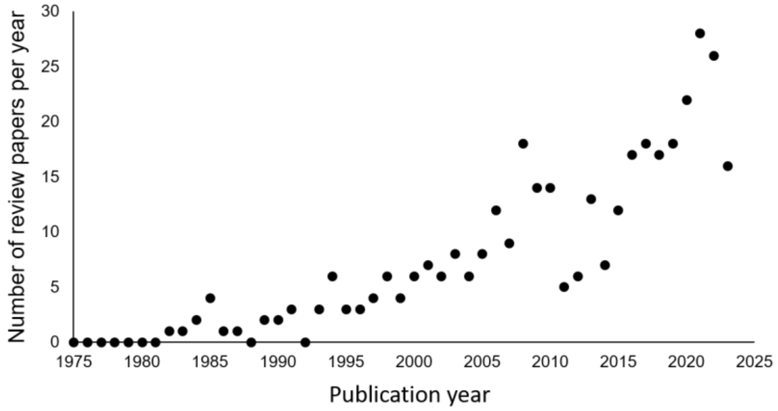

Figure 1: Evolution of the number of review articles on turtles per year, based on the Web of Science database. The numbers were extracted running a search for review articles with the keywords (“tortoise*” OR “chelonian*” OR “turtle*” OR “testudines*”) for each year since 1975 in WoS.

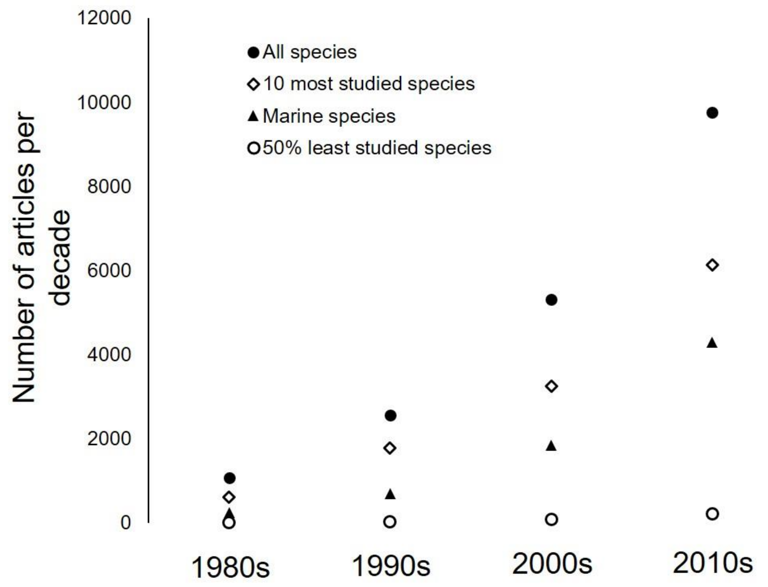

Figure 1~~2~~. Evolution of the number of articles on turtle species across time for all turtle species, for the 10 most studied species, for marine species only, and for the 50% least studied species.

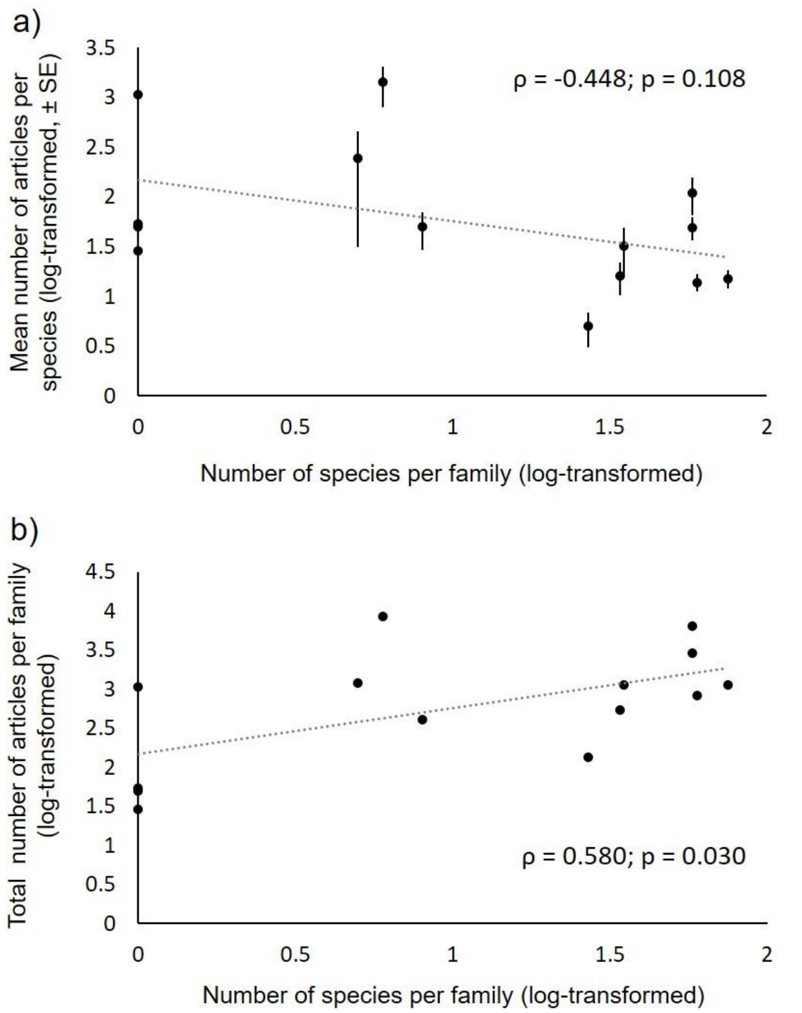

Figure 23. Association between the number of articles listed in the WoS (either the mean number in panel a, or the total number in panel b) and the number of species per family of Testudines. The results of Pearson tests are given (ρ = correlation coefficient; p = p-value) for each association.

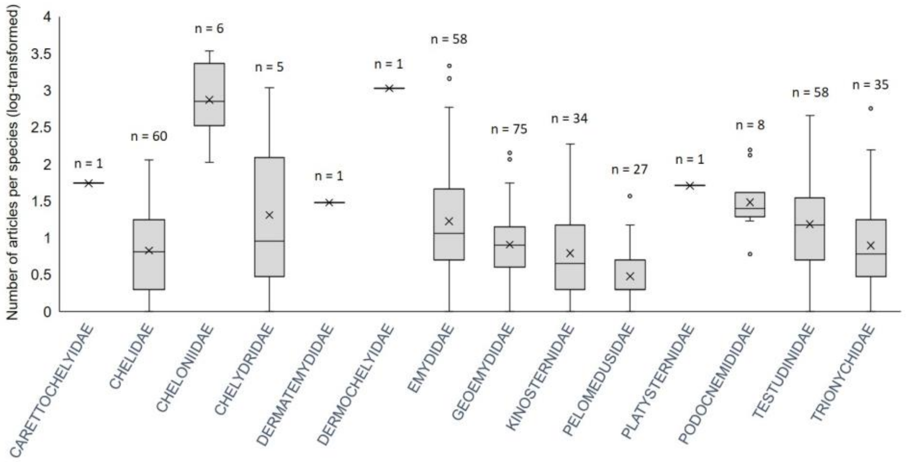

Figure 3. Box plot of the distribution of the number of articles (log-transformed) per species within each family of Testudines. Minimum, maximum, median, 25th and 75th quartiles are shown, as well as outliers. The number of species per family is indicated above each box, and x-symbols show the mean for each family.

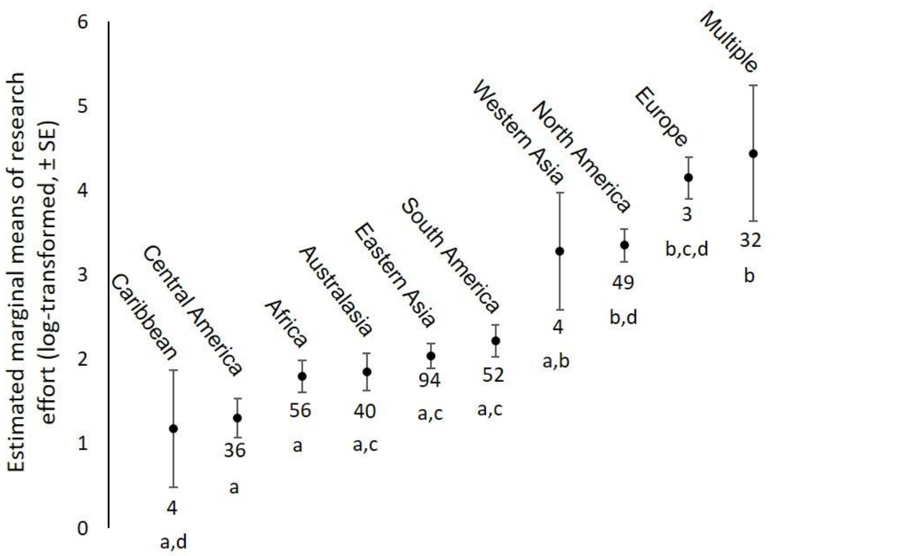

Figure 4. Variation in research effort across biogeographic realm. Estimated marginal means were extracted using the emmeans function (Lenth 2024). Different lLetters are used to illustrateindicate the significant ce of differences between geographic realms (e.g., Central America and Europe), whereas research effort does not differ significantly between . Two realmsregions that share the same letter are not significantly different (e.g., all realms with an a), whereas two realms that do not share the same letters are significantly different (e.g., Central America and Europe).

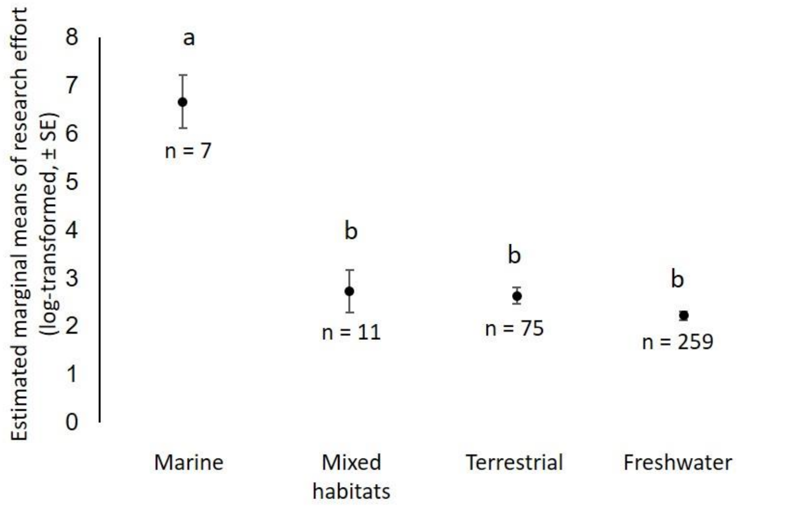

Figure 5. Variation in turtle research effort across habitats. Marine species were significantly more studied than turtles from any other habitat category, including species occurring in multiple habitats. The letters a and b indicate the significance ($p < 0.05$) of between-habitats comparisons (if the difference is significant, the two habitats were attributed different letters; if non-significant, they share the same letter). Different letters indicate significant differences between geographic realms (e.g., Central America and Europe), whereas research effort does not differ significantly between regions that share the same letter (e.g., all realms with an a).

Supplementary material 1. Best multi-predictor model explaining variation in research effort across species of Testudinidae after excluding marine species. Phylogeny was included as random effect, and we used the MCMCglmm procedure. Non-significant predictors excluded from the best model (pMCMC > 0.01 or ΔDIC > 5) are shown in italic style below the factors included in the best model. SCL = Straight-line Carapace Length; pm = posterior mean; CI = credibility interval, eff.samp = effective sample size. 110 species were included in this analysis.

Explanatory variables	pm	CI	eff.samp	pMCMC
Intercept	1.985	[0.439; 3.537]	1001	0.020
Historic range size	0.673	[0.391; 0.904]	868.1	<0.001
Introduction	0.479	[0.023; 0.964]	1001	0.048
Maximum SCL	0.449	[0.161; 0.752]	1001	0.002
Egg length	-0.444	[-0.747; -0.111]	1001	0.008
Biogeographic realm (ΔDIC = -21)				
Maximum longevity	*0.183*	*[-0.035; 0.434]*	*754.1*	*0.130*
Insularity	*0.273*	*[-1.269; 1.734]*	*1001*	*0.721*
Incubation	*-0.038*	*[-0.299; 0.333]*	*1001*	*0.761*
Country research rank	*0.010*	*[-0.299; 0.333]*	*1001*	*0.949*
Diet (ΔDIC = 10)				
Habitat (ΔDIC = 5)				

Supplementary material 2. Dataset including the research effort indices and traits considered in the analyses.

Supplementary material 3. R code used for the conducted analyses

Data Accessibilty Statement: The full dataset is available as Supplementary Material.

Competing Interests Statement: The authors declare no competing interest.

Author Contributions: SD: Writing – review & editing, Writing – original draft, Visualization, Methodology, Investigation, Funding acquisition, Formal analysis, Data curation, Conceptualization. **JD**: Writing – review & editing, Visualization, Methodology, Funding acquisition, Conceptualization

Acknowledgements: ~~SD and JD were funded by the Agence nationale de la recherche, France InvEcoF #ANR-22-CE02-0007 and the Fondation Fyssen, France.~~We are grateful to Tomasz Szczygielski and an anonymous reviewer for comments on a previous version of the manuscript.

Formatted: Font: (Default) Calibri

Formatted: Font: (Default) +Body (Calibri), 11 pt

Formatted: Font: (Default) +Body (Calibri), 11 pt

Dear Dr. Ducatez,

Thank you for submitting your manuscript to PLOS ONE. After careful consideration, we feel that it has merit but does not fully meet PLOS ONE’s publication criteria as it currently stands. Therefore, we invite you to submit a revised version of the manuscript that addresses the points raised during the review process.

==============================
However, after carefully reviewing the manuscript and considering the feedback provided by the selected reviewers, I recommend that this paper can be considered for publication after the author (s) undertake major revisions.
The key points that I would like to highlight are as follows:

1. **Methodology**: The study relies on the number of articles retrieved from the Web of Science. It is understood that a manual review of all relevant publications is impossible; however, semi-automated search strategies (WOS) may risk missing relevant data or including irrelevant ones. These issues should be more explicitly acknowledged. And discussed.

***Our answer** : We now discuss these issues more explicitly in both the methods and discussion (see lines 139 to 164 and 531 to 555). We have now developed this part of the discussion in more details to clearly underline the limitations of this index of research effort and suggest others that could give complementary information. However, as mentioned in our responses to the reviewers’ comments, we believe that this is a limited issue, as our objective was not to assess the absolute research effort, but to compare research effort across species. For example, we state when describing our methods: “Note that these indices are proxies for research effort, and not absolute estimates. They, for example, ignore the work that was published before 1975 as well as any reference that is not referenced in the WoS, including references from the grey literature. However, by constructing these indices, our purpose was to obtain a relative metric of research effort to compare species with each other, rather than an exact number of articles published per species. To test whether using different databases could affect this relative research effort score, we also compared the index obtained using the WoS database with the index extracted by Guedes et al. (2023) using Scopus and available for 343 of the 370 species considered.”*

2. **Geopolitical bias**: Research effort likely reflects whether species occur in scientifically active countries (e.g., USA, Europe, and China). This should be analyzed more directly rather than only by broad regions.

***Our answer**: We now included an extra variable considering each species presence/absence in the 10 and 20 most scientifically active countries. It is indeed a significant predictor, though it did not affect our conclusions with regards to the other predictors, and it was not retained in our multi-predictors model.*

3. **Taxonomy update**: Acknowledge the new 2025 Turtles of the World checklist (359 turtle and tortoise species now face extinction vs. 357 in 2021).

***Our answer**: We now updated the taxonomic list to the 2025 checklist, re-running all analyses after including the extra species. Note that this update was published in July 2025, after we submitted the first version of our manuscript.*

When submitting your revision, we need you to address these additional requirements.
1. Please ensure that your manuscript meets PLOS ONE's style requirements, including those for file naming. The PLOS ONE style templates can be found at https://journals.plos.org/plosone/s/file?id=wjVg/PLOSOne_formatting_sample_main_body.pdf and https://journals.plos.org/plosone/s/file?id=ba62/PLOSOne_formatting_sample_title_authors_affiliations.pdf
2. Please note that PLOS One has specific guidelines on code sharing for submissions in which author-generated code underpins the findings in the manuscript. In these cases, we expect all author-generated code to be made available without restrictions upon publication of the work. Please review our guidelines at https://journals.plos.org/plosone/s/materials-and-software-sharing#loc-sharing-code and ensure that your code is shared in a way that follows best practice and facilitates reproducibility and reuse.
***Our answer**: We have added our code to the Suppl. Mat.*

3. Thank you for stating the following financial disclosure:
“Agence nationale de la recherche, France InvEcoF #ANR-22-CE02-0007
Fondation Fyssen, France.”
Please state what role the funders took in the study. If the funders had no role, please state: "The funders had no role in study design, data collection and analysis, decision to publish, or preparation of the manuscript."
If this statement is not correct you must amend it as needed.
Please include this amended Role of Funder statement in your cover letter; we will change the online submission form on your behalf.
***Our answer**: Changed accordingly*

4. Thank you for stating the following in the Acknowledgments Section of your manuscript:
“SD and JD were funded by the Agence nationale de la recherche, France InvEcoF #ANR-22-CE02-0007 and the Fondation Fyssen, France.”
We note that you have provided funding information that is currently declared in your Funding Statement. However, funding information should not appear in the Acknowledgments section or other areas of your manuscript. We will only publish funding information present in the Funding Statement section of the online submission form.
Please remove any funding-related text from the manuscript and let us know how you would like to update your Funding Statement. Currently, your Funding Statement reads as follows:
“Agence nationale de la recherche, France InvEcoF #ANR-22-CE02-0007
Fondation Fyssen, France.”
Please include your amended statements within your cover letter; we will change the online submission form on your behalf.
***Our answer**: We have now removed the funding information from the acknowledgements section and included our funding statement and role of funder statement in the cover letter.*

5. Please include captions for your Supporting Information files at the end of your manuscript, and update any in-text citations to match accordingly. Please see our

Supporting Information guidelines for more information: http://journals.plos.org/plosone/s/supporting-information.
Our answer*: We have now included the captions for Supporting Information at the end of our manuscript.*

If the reviewer comments include a recommendation to cite specific previously published works, please review and evaluate these publications to determine whether they are relevant and should be cited. There is no requirement to cite these works unless the editor has indicated otherwise.

Additional Editor Comments:

Dear authors,

After carefully reviewing the manuscript and considering the feedback provided by the selected reviewers, I recommend that this paper can be considered for publication after the author (s) undertake major revisions.
For more information, please see the comments from reviewers.

Reviewers' comments:

Reviewer's Responses to Questions
Comments to the Author

Review Comments to the Author

Reviewer #1: This manuscript addresses a timely and important issue uneven research effort across Testudines species, highlighting biases and gaps that affect both ecological understanding and conservation actions. The study is ambitious in scale, data-rich, and well-documented, but several concerns need to be addressed before it can be considered for publication in PLOS ONE:
Our answer: *We thank the reviewer for comments that helped improving the manuscript.*

1. Justification of Methodological Choices (Search Strategy & Data Limitations)
The authors rely heavily on the number of articles retrieved from Web of Science using species names. While they attempt to validate the approach through correlation with Scopus data and synonym comparisons, this method can still under- or over-estimate research effort (e.g., name ambiguity, short genus names). Consider a more detailed justification or a sensitivity analysis beyond correlation coefficients.
Our answer*: We further developed this important point in the discussion (see lines 535-547). Note that our index of research effort is not meant to provide an absolute, but a relative metric for research effort, as mentioned in the methods section. The objective is to compare species, not to know exactly how much research was devoted to each specie. As a result, the number of articles on large online databases such as Scopus and Web of Science have been largely used in previous work investigating research effort in other taxonomic groups. We already provide multiple sensitivity analyses, considering different search strategies in the search engines (title only, or more global search), multiple search engines (Scopus or Web of Science) and considering synonyms or not. Although other sensitivity analyses could be considered, they would rely on extracting information from the grey literature or an in-depth analysis of each of the thousands of published articles that focus on turtles. We are not competent to automatize this type of analysis at this stage, and do not believe that it would bring much more information than confirming, with yet an extra sensitivity analysis, that all metrics for research effort are highly correlated. Note also that we are using an index that has been used by multiple authors in previously published work (including reptile work), and we have included more tests of the robustness of our index than most published work investigating taxonomic variation in*

research effort. This approach is a standard way to assess research effort and has been used by several papers assessing the heterogeneous study effort across species and its drivers (e.g., to illustrate how frequently these indices are used, here are a few examples of articles using similar indices, in addition to the articles already cited in our manuscript).

Adamo M, Chialva M, Calevo J et al (2021) Plant scientists' research attention is skewed towards colourful, conspicuous and broadly distributed flowers. Nat Plants 7:574–578.
Mammola S, Adamo M, Antić D et al (2023) Drivers of species knowledge across the tree of life. eLife 12:RP88251
Simoncini, A., Ficetola, G.F. & Lattanzi, M. Taxonomic bias towards charismatic and easy-to-find mammals shapes knowledge of parasites. *Biodivers Conserv* (2025). https://doi.org/10.1007/s10531-025-03147-1
Tam J, Lagisz M, Cornwell W et al (2022) Quantifying research interests in 7,521 mammalian species with h-index: a case study. Gigascience 11:giac074.
Wilson JRU, Proches S, Braschler B et al (2007) The (bio)diversity of science reflects the interests of society. Front Ecol Environ 5:409–414. https://doi.org/10.1890/060077.1

2. Treatment of Sea Turtles as Outliers
While the rationale to exclude sea turtles from the multivariate analysis is understandable due to their high representation, this introduces inconsistency: the main claim about marine species dominating research becomes methodologically decoupled from the model-based inference. Consider conducting parallel analyses (with and without sea turtles) to better understand their effect.
***Our answer**: We agree with the reviewer that parallel analyses including vs excluding sea turtles for the global model would be preferable. We now ran the multivariate analysis twice, either with or without sea turtles. We show the results of the model on all species in the main text, and the results of the model excluding sea turtles in the Supplementary Material. Note that the results were consistent between these two datasets.*

3. Insufficient Integration of Conservation Implications
The Discussion does mention that endangered species are not more studied, but the conservation implications remain superficial. The authors should provide clearer suggestions: How can their findings be translated into priority research planning, especially for data-deficient taxa?
***Our answer**: We added more discussion on this aspect lines 524-530. Note that our analyses mostly identify a need for more work to clearly identify how these research gaps can affect our ability to design conservation plans for endangered species, as discussed in the paragraph lines 507-530. Specifically, our findings underline that species that are at a higher risk of extinction, though they should be prioritized by researchers to favor their conservation and/or recovery, are not more often studied than less threatened species. We believe that our index or research effort can be used as an extra argument to help obtaining funding for under researched species (either data deficient or unassessed ones) and endangered ones.*

4. Trait–Effort Relationships Need Clarification

The final multivariate model includes biologically meaningful traits like body size, longevity, and clutch size. However, clutch size switches from a positive association in the univariate to negative in the multivariate model, without adequate explanation. Please address potential collinearity or scaling issues, and interpret biological significance more clearly.
Our answer*: We agree with the reviewer that this switch is unclear and could be due to collinearity issues, or differences in the set of species considered in the monovariate and multivariate models. The correlation between max SCL and clutch size was of 0.568 – to be safe and avoid any risk of collinearity, we therefore decided to exclude clutch size from the multivariate model.*

Note that to better understand the switch in clutch size effect (even though we are not presenting this switch in the MS anymore), we built a model with clutch size as the only predictor considering the species that were included in the multivariate model only, to determine whether the switch could be due to a change in the set of species considered. This resulted in a non-significant effect of clutch size, supporting the idea that both collinearity between clutch size and SCL, and the subsample of species on which the multivariate analysis could be conducted, explain the discrepancy between the univariate and multivariate models with regard to the clutch size effect.

5. Geographic Bias Discussion Needs Expansion
The paper identifies geographic disparities (e.g., North America vs. Africa), but lacks explanation of underlying drivers (e.g., funding, infrastructure, language bias). Incorporate literature from bibliometric studies or conservation research bias.
Our answer: *We have now added an extra analysis as suggested by reviewer 2, comparing species present in the 10 or 20 most scientifically active countries with species that are absent from these countries. We also added sentences of discussion on this aspect lines 459-461. This was also discussed in the paragraph lines 499-506.*

Suggested Additional Analyses (if feasible):
1- A network map of author affiliations vs. species studied might add depth to understanding geographic biases.
Our answer*: Although we agree that this would be an interesting addition, this was out of the scope of the manuscript and would require data extraction from each of the thousands of articles on the different turtle species. All of these data extractions were conducted by hand, so this would add a massive amount of work. A future study using AI might bring this information more easily.*

2- A comparison between publication count and conservation funding per species or region, if data are available.
Our answer*: We also agree that this would be a welcome future study, though this was out of the scope of our study. We thank the reviewer for this suggestion which we added as a perspective in the discussion (see lines 528-530).*

Conclusion:
This manuscript has the potential to make a meaningful contribution to reptile conservation and bibliometric ecology, but it requires significant revisions to improve its methodological rigor, analytical clarity, and conservation relevance.

Please revise the manuscript accordingly and provide a detailed point-by-point rebuttal.
Our answer*: We are grateful to the reviewer for constructive comments that helped us improving the quality of our study.*

Reviewer #2: This is an interesting contribution with implications not only for the turtles, but for the approach to biodiversity studies as a whole. I need to stress that I am not an expert in statistics, so those should be checked by a specialist in the field. However, I can provide some insights from the general biological and herpetological point of view. Overall, I think the paper is well-written and it should be published but I see some issues, mostly with how the results are presented, as I think that the potential of the data is not fully utilized and some aspects may require slight refinement or a better justification.
Our answer*: We thank the reviewer for comments that helped improving the manuscript.*

The topic is somewhat difficult from the methodological standpoint because, given the vast number of published papers, it is not really possible to review their contents manually, whereas any semiautomatic approach (such as the one employed by the Authors) will unavoidably result in some errors. For example, some papers providing valuable information on some species may be overlooked, because they do not include the full name of the species in any of the fields searchable by the Web of Science, but instead use, e.g., only the generic name or the name of the family (as I understand, the Authors did not look for those, only for the binomina). On the other hand, in some cases a specific name may be used in an abstract, e.g., solely in a comparative context without any meaningful information on that species presented in the paper (false hits), or as a synonym (double hits when both variants of a name are searched for). I understand that these instances are inevitable and while some workarounds can be proposed, they cannot be fully eliminated. I also understand that the cutoff used by the Authors (1975) is a sad necessity due to the increasingly limited availability of data for processing (that could be, to some extent, helped by inclusion of, e.g., the Biodiversity Heritage Library) but also due to complications regarding the synonymy and more frequent usage of sole generic names in those older papers. Unfortunately, this leaves out some important anatomical and ecological literature, also regarding the less popular taxa, which is still useful and informative and which in some cases may render restudy of those species in modern times less of a priority, thus causing them to appear even less studied in the selected timeframe. This should be perhaps acknowledged slightly more in the text (although the Authors already referred to that problem) but other than that we cannot do much about it and I accept that as an inherent limitation of that kind of research.
Our answer*: As mentioned in our response to the editor and reviewer 1, we developed the discussion on this issue, although we already discussed it in both the methods and discussion of the previous version of the MS (see lines 139 to 164 and 531 to 555). As mentioned in the manuscript as well as in response to reviewer 1, the objective of this research index is not to provide an absolute metric for species research effort, but an index that allows to compare species. There is a lot more literature on turtles that is not included in WOS or Scopus, and conversely, some articles that came up in our search did not necessarily focus on the species of interest (even if the species name is given in the abstract, it does not systematically imply that the article is focused on the species of interest). Our purpose was however not to produce an exact and exhaustive list of articles per species, but rather to compare the relative amount of research that has been devoted to each species. Although pre-1975 research can be very important and should not be neglected by researchers working on particular species, the amount of research articles that have been produced since 1975 is overwhelmingly larger, by multiple orders of magnitude.*

This increase in the quantity of research conducted since the 1970 has been described elsewhere in details (see also our response to another comment of reviewer 2). So even if it is possible that some species that were under studied since 1975 were the focus of several studies before that date, it would likely only slightly alter their score, and be of very little effect on the global trends that we aimed at testing here. We have now added more discussion on this aspect lines 535-547.

One oversight and potential problem I see with communicating the methodology is related to Fig. 1. It presents the number of review papers on turtles. Unfortunately, I don't see any notion of such a search in the Methods section and the methodology for compiling that charts seems to be only explained in the figure caption. However, this does not seem right for two reasons. (1) using solely the given query ("tortoise*" OR "chelonian*" OR "turtle*" OR "testudines*") one is expected to find not only papers relevant to the reviewed manuscript, but potentially also papers completely out of scope, e.g., on extinct stem turtles, turtle-derived materials (tortoiseshell), arthropods (tortoise beetles), etc., so the result does not really say much. (2) I tried to see how badly out of scope the results are in that case, but I could not replicate the Author's results. I searched for the provided keywords, string copied directly from the figure caption, and document type set to Review Article. I will give the values for the year 2022, because this is the largest number in the Author's search and shows the problem the most easily, but there were also differences for other years. When searching in Author Keywords, 21 articles were found. This is much below the over 25 papers found by the Authors for that year. When searching in Keywords plus, the search yielded 58 papers for that year – this is much more. Also, as expected, the search found lots of papers on fossil turtles and some nonsensical ones (e.g., "Plant phenolics: neglected secondary metabolites in plant stress tolerance"). Please check and preferably refine – the Author Keywords yielded more sensible results in that case and about 250 papers total, so it is doable to leave out those that are out of scope). In case the Authors searched in Keywords Plus and manually rejected results out of scope (possible but I think unlikely, as that search produced over 700 results for me) then kudos to them but please communicate that in the Methods!
Our answer: *We have now removed this figure, which was only meant to provide a simple illustration on the increase in reviews and large taxonomic scale comparative studies on the Testudinae group to further illustrate the need for our study, in the introduction. This is why we did not mention this approach in the methods, but instead directly in the introduction. Our purpose was not to be exhaustive, but to show the relative increase in these large-scale studies that sometimes rely on the expectation that all species are equally known, or that aim at providing patterns or mechanisms that are true at the scale of the entire clade. Why the reviewer did not find the same numbers as us is unclear to us, as we reran these searches and found very similar numbers for each year. Note however that the WoS database is frequently changed, with new references added or removed regularly, which may explain this discrepancy (though to our experience, the differences are usually minor). We removed the figure to avoid any misunderstanding with regards to the interpretation of the figure, and because it was not absolutely needed.*

The most important aspect, which is currently very indirectly and tangentially tackled by the Authors but certainly requires more attention as a potential good predictor of research effort, is simply whether the species in question is native to territories of

more scientifically active countries (e.g., with better financing of scientific institutions). This may very well explain why some taxa became sort of model species for turtles and why they continue to gain attention while other remain neglected. Given the huge differences in research effort between species, some receiving next to no attention and some literally thousands of papers, I suspect that such geopolitical factors may not only be significant, but even overpower other traits – especially that, if I see correctly, all but one species with over 150 papers on their account occur either in the USA, Western or Southern Europe, or China, whereas none of at least several dozens of the least studied species (except of some artificially underestimated instances – see below) occurs there. Currently, the Authors only briefly consider that topic in terms of generalized geographic regions (North America, Central America, South America, etc.) but I am afraid this misses a lot of nuance, especially that a number of taxa falls into the uninformative “Multiple” category. Analyzing whether there is a statistically significant correlation between the research interest and the overlap of the species range with the territory of one or more of, let’s say, the 10 or 20 most scientifically active countries may prove extremally interesting and should not require a lot of additional work and may explain why some taxa inhabiting the same large-scale geographic area differ significantly in attention. I propose that the Authors check and discuss whether this is indeed the case.
Our answer*: We followed the reviewer’s advice and compared the research effort of the Testudinae species occurring the 10 or 20 most scientifically active species, using a research ranking score per country, and ranking each species according to whether or not it occurs within the top 10 or top 20 countries. As predicted by the reviewers, there is a strong discrepancy, with species occurring in the most scientifically active countries being also the focus of more research. We now include this variable in both a univariate and multivariate models, though it was not a significant predictor in the multivariate model (likely because it is redundant with the geographic region that was already included). A diversity of socio-economic predictors are likely to affect research effort, including factors related to the cultural and economic importance of some taxa; other works have focused on these types of predictors to understand their role (though to the best of our knowledge, this has not been investigated in turtles). In this study, however, our purpose was not to test these questions in depth, nor to disentangle all drivers of variation in research effort. Instead, we were mostly interested in underlining the existence of research-biases across Testudinae species, and of their potential consequences for the interpretation of large scale comparative analyses. Elucidating all the causes of research-biases was out of the scope of our study.*

Additionally, the Authors should address the problem of recently established species, such as Kinosternon steindachneri (2013) and Sternotherus intermedius (2017), which did not yet accumulate a significant bibliography of their own, but were considered historically as part (subspecies or subpopulation) of their closely related species (in the case of Kinosternon steindachneri – Kinosternon subrubum) or hybrids (in the case of Sternotherus intermedius). As a result, true research effort devoted to them is either difficult to establish or artificially underestimated, in turn possibly skewing indices for corresponding supraspecific taxa and traits. If possible, in those instances it would be advisable to track the research effort on the subspecies rather than species level. Otherwise, they should probably be either considered jointly with their sister species or left out, unless their impact is confirmed to be insignificant.

Our answer*: We agree that a limitation of our index of research effort is that it does not give the "true" research effort value for each species. It was however not our purpose, as we aimed at obtaining a relative index comparable across species (see our answer to previous comments). In the case of recently established species, if they were recently split and previously considered as a subspecies of other species, the subspecies name was included in the list of synonyms, and therefore considered (based on synonymy) even if the species was only recently recognized as a full species. Note that tracking subspecies research effort is particularly challenging as most studies do not precise which subspecies is investigated, but only provide the species name.*

Another thing that may deserve reconsideration on the part of the Authors is the usage of full decades in some of their analyses, despite their timeframe starting and ending in the middle of the decades (1975–2024). Five-year bins may perhaps be more adequate?
Our answer*: Our purpose here was to test for changes in research effort discrepancy over time. Since the split into decades was sufficient to test and detect this effect, we decided not to include the five-years steps which would require to rerun every search species by species for each 5-year period, with no clear extra information for our understanding of variation in research effort across time.*

Finally, the paper is mostly built on the taxonomical framework established by the 2021 ninth edition of the Turtles of the World checklist. However, a new edition (tenth, 2025) is already available, so the manuscript should be updated to acknowledge that. The new edition distinguishes 359 extant species instead of 357 recognized by TotW 2021. Whether those new species should be considered on their own depends on the approach (see above), but a lot of attention has been given to turtle extinctions in the new volume, so it is relevant at least in that context.
Our answer*: We have now updated our dataset with this 2025 edition of the checklist and reran all analyses considering this new framework (we also updated the historic range size values). Note that this new checklist was published on July 25th, 2025 – so after we'd submitted the previous version of the manuscript. Note that we also updated the scores of extinction risk and population trend for all species using the newly published IUCN red list (i.e., the 2025-2 version, from October 2025).*

More detailed notes are provided below:
P. 3, lines 43–44 („Testudines emerged during the Triassic and survived two mass extinction crises that eliminated several other groups of reptiles at the end of the Permian and Cretaceous"): This statement is incorrect for two reasons. First, the Testudines are the crown group composed from two extant clades: the side-necked turtles (Pleurodira) and hidden-necked turtles (Cryptodira). The exact time of divergence or those two clades (and, in consequence, the appearance of the Testudines) is somewhat contentious, as it depends on the classification of some poorly known fossil taxa, but it was certainly later than in the Triassic, most probably in the Middle to Late Jurassic. All Triassic turtles are stem taxa so they belong to Testudinata, not Testudines. Second, the Permian was before the Triassic, so neither the Testudines nor the Testudinata (as a group appearing at the end of the Triassic) could not survive the Permian extinction. I imagine that the Authors may confuse here the Testudines, Testudinata, and Pan-Testudines/Pantestudinata (a larger group including also shell-less turtle relatives, possibly also the Permian

Eunotosaurus africanus), but these not the same and the paper focuses solely on Testudines proper, so there is no need to bring those earlier forms up.
***Our answer**: We thank the reviewer for this important point, apologize for our mistake and removed this sentence from the introduction.*

P. 3, lines 48 and 87 („Squamates", "Amphibians"): when anglicized, the name should not be capitalized (so „Squamata" and "Amphibia" would be correct but in that case „squamates" and "amphibians").
***Our answer**: Changed accordingly.*

P. 4, lines 104–105 ("fast lifestyle", "slow lifestyle"): this seem somewhat poorly fitting terms, maybe "life cycle"?
***Our answer**: We changed these into fast and slow life history strategies (see lines 103-104).*

P. 6, line 186 ("maximum standard carapace length"): I am unfamiliar with such a measurement and I think the Authors mean here the "maximum straight-line carapace length" (a common measurement used for turtles and typically abbreviated as SCL, also used by Rhodin et al., 2021). If so, please also correct elsewhere. If not, please define.
***Our answer**: The reviewer is right and we changed this (into "straight-line carapace length") throughout the manuscript.*

P. 8, line 313: can this increase be partially explained by the incomplete sampling of older articles in the WoS, e.g., published in historical but now discontinued journals which are not indexed?
***Our answer**: page 8 line 313 of the previous version of the MS refers to results showing the proportion of variance in research effort explained by taxonomy. This comment is likely referring to the previous paragraph and page, which shows the increase in research effort across time (lines 305-309)? If so, the increase in the number of publications across time is known and well documented globally (not only in biology) and was expected for Testudines research as well. We decided not to discuss this question in our manuscript is this was out of the scope of our study, and it has been the focus of diverse studies. Note that discontinued journals are also included in the WoS or Scopus databases. A few examples of work showing and discussing the causes of this global increase:*
https://direct.mit.edu/qss/article/5/4/823/124269/The-strain-on-scientific-publishing
Thekwall and Sud 2022 Scopus 1900–2020: Growth in articles, abstracts, countries, fields, and journals. Quantitative Science Studies *(2022) 3 (1): 37–50.*
Fire and Guestrin 2019 Over-optimization of academic publishing metrics: observing Goodhart's Law in action. GigaScience*, Volume 8, Issue 6, giz053*

P. 10, line 401 ("Trachemys scrypta"): Trachemys scripta.
***Our answer**: Changed accordingly.*

Table 1: I think I understand what the Authors want to show here, but at the same time there is something missing. The juxtaposition of the taxonomic richness of the family and the number of papers is some information, but this does not say too much without some measure of the evenness of coverage. As presented, the Chelydridae are in the third spot with a decent 243 papers per species, which looks very good, but

what of it, if nearly 90% of that (!) is devoted to one species. Conversely, the Geoemydidae only have 16 papers per species, so they appear significantly understudied compared to the chelydrids, but in reality the research effort is much more evenly distributed. In consequence, I feel the message here falls into the same pitfall which the Authors try to solve in their manuscript – we see an averaged picture but completely miss how some species are understudied. Also, the numbers alone are difficult to visualize, so the nice data that the Authors gathered get somewhat lost. I would strongly suggest presenting this in some more visual way. Perhaps a bar chart with the bars subdivided into sections corresponding to the proportional research attention received by individual species, or a pie chart with subdivided slices? I get it that the number of species within many families is too large to show all of them within a single bar/slice, but the differences in attention between them are large enough that the best studied species within each family should be visible as individual portions, whereas the least studied species may be grouped together. This would not only nicely show the differences between the families, but it would also present the disproportions in the effort within each of them and it would also be much more visually appealing.
Our answer*: We thank the reviewer for this suggestion. We have now added to this table the standard error for each family as an index of research effort evenness across species within families. We also followed the reviewer advice by adding a new figure with a boxplot showing the distribution of research effort within each family, with the four quartiles, mean, median and number of species per family (see Figure 2).*

Fig. 1 caption: “Evolution” is not a good term here, I would suggest just leaving “The number of review articles” etc.
Our answer*: We have now removed this figure from the manuscript.*

Figs 4 and 5: Please explain what do “a”, “b”, and “c” mean.
Our answer*: We now provide this information in both captions.*
We are very grateful to Tomasz Szczygielski for constructive comments that helped us to improve our manuscript.
