# Supplementary material for: Which turtle should I study? Uneven distribution of research effort across Testudines species
Source: PLoS One. 2026 Apr 20;21(4):e0347198. doi: 10.1371/journal.pone.0347198 (PMC13094973; doi:10.1371/journal.pone.0347198)
Supplement: Attachment — Submitted filename: Response to reviewers.docx [file pone.0347198.s009.docx]

Dear Dr. Ducatez,

Thank you for submitting your manuscript to PLOS ONE. After careful consideration, we feel that it has merit but does not fully meet PLOS ONE’s publication criteria as it currently stands. Therefore, we invite you to submit a revised version of the manuscript that addresses the points raised during the review process.

==============================

However, after carefully reviewing the manuscript and considering the feedback provided by the selected reviewers, I recommend that this paper can be considered for publication after the author (s) undertake major revisions.

The key points that I would like to highlight are as follows:

1. **Methodology**: The study relies on the number of articles retrieved from the Web of Science. It is understood that a manual review of all relevant publications is impossible; however, semi-automated search strategies (WOS) may risk missing relevant data or including irrelevant ones. These issues should be more explicitly acknowledged. And discussed.

***Our answer*** *: We now discuss these issues more explicitly in both the methods and discussion (see lines 139 to 164 and 531 to 555). We have now developed this part of the discussion in more details to clearly underline the limitations of this index of research effort and suggest others that could give complementary information. However, as mentioned in our responses to the reviewers’ comments, we believe that this is a limited issue, as our objective was not to assess the absolute research effort, but to compare research effort across species. For example, we state when describing our methods: “Note that these indices are proxies for research effort, and not absolute estimates. They, for example, ignore the work that was published before 1975 as well as any reference that is not referenced in the WoS, including references from the grey literature. However, by constructing these indices, our purpose was to obtain a relative metric of research effort to compare species with each other, rather than an exact number of articles published per species. To test whether using different databases could affect this relative research effort score, we also compared the index obtained using the WoS database with the index extracted by Guedes et al. (2023) using Scopus and available for 343 of the 370 species considered.”*

2. **Geopolitical bias**: Research effort likely reflects whether species occur in scientifically active countries (e.g., USA, Europe, and China). This should be analyzed more directly rather than only by broad regions.

***Our answer****: We now included an extra variable considering each species presence/absence in the 10 and 20 most scientifically active countries. It is indeed a significant predictor, though it did not affect our conclusions with regards to the other predictors, and it was not retained in our multi-predictors model.*

3. **Taxonomy update**: Acknowledge the new 2025 Turtles of the World checklist (359 turtle and tortoise species now face extinction vs. 357 in 2021).

***Our answer****: We now updated the taxonomic list to the 2025 checklist, re-running all analyses after including the extra species. Note that this update was published in July 2025, after we submitted the first version of our manuscript.*

When submitting your revision, we need you to address these additional requirements.

1. Please ensure that your manuscript meets PLOS ONE's style requirements, including those for file naming. The PLOS ONE style templates can be found at

<https://journals.plos.org/plosone/s/file?id=wjVg/PLOSOne_formatting_sample_main_body.pdf> and

<https://journals.plos.org/plosone/s/file?id=ba62/PLOSOne_formatting_sample_title_authors_affiliations.pdf>

2. Please note that PLOS One has specific guidelines on code sharing for submissions in which author-generated code underpins the findings in the manuscript. In these cases, we expect all author-generated code to be made available without restrictions upon publication of the work. Please review our guidelines at <https://journals.plos.org/plosone/s/materials-and-software-sharing#loc-sharing-code> and ensure that your code is shared in a way that follows best practice and facilitates reproducibility and reuse.

***Our answer****: We have added our code to the Suppl. Mat.*

3. Thank you for stating the following financial disclosure:

“Agence nationale de la recherche, France InvEcoF #ANR-22-CE02-0007

Fondation Fyssen, France.”

Please state what role the funders took in the study.  If the funders had no role, please state: "The funders had no role in study design, data collection and analysis, decision to publish, or preparation of the manuscript."

If this statement is not correct you must amend it as needed.

Please include this amended Role of Funder statement in your cover letter; we will change the online submission form on your behalf.

***Our answer****: Changed accordingly*

4. Thank you for stating the following in the Acknowledgments Section of your manuscript:

“SD and JD were funded by the Agence nationale de la recherche, France InvEcoF #ANR-22-CE02-0007 and the Fondation Fyssen, France.”

We note that you have provided funding information that is currently declared in your Funding Statement. However, funding information should not appear in the Acknowledgments section or other areas of your manuscript. We will only publish funding information present in the Funding Statement section of the online submission form.

Please remove any funding-related text from the manuscript and let us know how you would like to update your Funding Statement. Currently, your Funding Statement reads as follows:

“Agence nationale de la recherche, France InvEcoF #ANR-22-CE02-0007

Fondation Fyssen, France.”

Please include your amended statements within your cover letter; we will change the online submission form on your behalf.

***Our answer****: We have now removed the funding information from the acknowledgements section and included our funding statement and role of funder statement in the cover letter.*

5. Please include captions for your Supporting Information files at the end of your manuscript, and update any in-text citations to match accordingly. Please see our Supporting Information guidelines for more information: <http://journals.plos.org/plosone/s/supporting-information>.

***Our answer****: We have now included the captions for Supporting Information at the end of our manuscript.*

If the reviewer comments include a recommendation to cite specific previously published works, please review and evaluate these publications to determine whether they are relevant and should be cited. There is no requirement to cite these works unless the editor has indicated otherwise. 

Additional Editor Comments:

Dear authors,

After carefully reviewing the manuscript and considering the feedback provided by the selected reviewers, I recommend that this paper can be considered for publication after the author (s) undertake major revisions.
For more information, please see the comments from reviewers.


Reviewers' comments:

Reviewer's Responses to Questions

**Comments to the Author**

Review Comments to the Author

Reviewer #1: This manuscript addresses a timely and important issue uneven research effort across Testudines species, highlighting biases and gaps that affect both ecological understanding and conservation actions. The study is ambitious in scale, data-rich, and well-documented, but several concerns need to be addressed before it can be considered for publication in PLOS ONE:

***Our answer:*** *We thank the reviewer for comments that helped improving the manuscript.*

1. Justification of Methodological Choices (Search Strategy & Data Limitations)
The authors rely heavily on the number of articles retrieved from Web of Science using species names. While they attempt to validate the approach through correlation with Scopus data and synonym comparisons, this method can still under- or over-estimate research effort (e.g., name ambiguity, short genus names). Consider a more detailed justification or a sensitivity analysis beyond correlation coefficients.

***Our answer****: We further developed this important point in the discussion (see lines 535-547). Note that our index of research effort is not meant to provide an absolute, but a relative metric for research effort, as mentioned in the methods section. The objective is to compare species, not to know exactly how much research was devoted to each specie. As a result, the number of articles on large online databases such as Scopus and Web of Science have been largely used in previous work investigating research effort in other taxonomic groups. We already provide multiple sensitivity analyses, considering different search strategies in the search engines (title only, or more global search), multiple search engines (Scopus or Web of Science) and considering synonyms or not. Although other sensitivity analyses could be considered, they would rely on extracting information from the grey literature or an in-depth analysis of each of the thousands of published articles that focus on turtles. We are not competent to automatize this type of analysis at this stage, and do not believe that it would bring much more information than confirming, with yet an extra sensitivity analysis, that all metrics for research effort are highly correlated. Note also that we are using an index that has been used by multiple authors in previously published work (including reptile work), and we have included more tests of the robustness of our index than most published work investigating taxonomic variation in research effort. This approach is a standard way to assess research effort and has been used by several papers assessing the heterogeneous study effort across species and its drivers (e.g., to illustrate how frequently these indices are used, here are a few examples of articles using similar indices, in addition to the articles already cited in our manuscript).*

Adamo M, Chialva M, Calevo J et al (2021) Plant scientists’ research attention is skewed towards colourful, conspicuous and broadly distributed flowers. Nat Plants 7:574–578.

Mammola S, Adamo M, Antić D et al (2023) Drivers of species knowledge across the tree of life. eLife 12:RP88251

Simoncini, A., Ficetola, G.F. & Lattanzi, M. Taxonomic bias towards charismatic and easy-to-find mammals shapes knowledge of parasites. *Biodivers Conserv* (2025). <https://doi.org/10.1007/s10531-025-03147-1>

Tam J, Lagisz M, Cornwell W et al (2022) Quantifying research interests in 7,521 mammalian species with h-index: a case study. Gigascience 11:giac074.

Wilson JRU, Proches S, Braschler B et al (2007) The (bio)diversity of science reflects the interests of society. Front Ecol Environ 5:409–414. <https://doi.org/10.1890/060077.1>

2. Treatment of Sea Turtles as Outliers
While the rationale to exclude sea turtles from the multivariate analysis is understandable due to their high representation, this introduces inconsistency: the main claim about marine species dominating research becomes methodologically decoupled from the model-based inference. Consider conducting parallel analyses (with and without sea turtles) to better understand their effect.

***Our answer****: We agree with the reviewer that parallel analyses including vs excluding sea turtles for the global model would be preferable. We now ran the multivariate analysis twice, either with or without sea turtles. We show the results of the model on all species in the main text, and the results of the model excluding sea turtles in the Supplementary Material. Note that the results were consistent between these two datasets.*

3. Insufficient Integration of Conservation Implications
The Discussion does mention that endangered species are not more studied, but the conservation implications remain superficial. The authors should provide clearer suggestions: How can their findings be translated into priority research planning, especially for data-deficient taxa?

***Our answer****: We added more discussion on this aspect lines 524-530. Note that our analyses mostly identify a need for more work to clearly identify how these research gaps can affect our ability to design conservation plans for endangered species, as discussed in the paragraph lines 507-530. Specifically, our findings underline that species that are at a higher risk of extinction, though they should be prioritized by researchers to favor their conservation and/or recovery, are not more often studied than less threatened species. We believe that our index or research effort can be used as an extra argument to help obtaining funding for under researched species (either data deficient or unassessed ones) and endangered ones.*

4. Trait–Effort Relationships Need Clarification
The final multivariate model includes biologically meaningful traits like body size, longevity, and clutch size. However, clutch size switches from a positive association in the univariate to negative in the multivariate model, without adequate explanation. Please address potential collinearity or scaling issues, and interpret biological significance more clearly.
***Our answer****: We agree with the reviewer that this switch is unclear and could be due to collinearity issues, or differences in the set of species considered in the monovariate and multivariate models. The correlation between max SCL and clutch size was of 0.568 – to be safe and avoid any risk of collinearity, we therefore decided to exclude clutch size from the multivariate model.*

*Note that to better understand the switch in clutch size effect (even though we are not presenting this switch in the MS anymore), we built a model with clutch size as the only predictor considering the species that were included in the multivariate model only, to determine whether the switch could be due to a change in the set of species considered. This resulted in a non-significant effect of clutch size, supporting the idea that both collinearity between clutch size and SCL, and the subsample of species on which the multivariate analysis could be conducted, explain the discrepancy between the univariate and multivariate models with regard to the clutch size effect.*

5. Geographic Bias Discussion Needs Expansion
The paper identifies geographic disparities (e.g., North America vs. Africa), but lacks explanation of underlying drivers (e.g., funding, infrastructure, language bias). Incorporate literature from bibliometric studies or conservation research bias.
***Our answer:*** *We have now added an extra analysis as suggested by reviewer 2, comparing species present in the 10 or 20 most scientifically active countries with species that are absent from these countries. We also added sentences of discussion on this aspect lines 459-461. This was also discussed in the paragraph lines 499-506.*

Suggested Additional Analyses (if feasible):
1- A network map of author affiliations vs. species studied might add depth to understanding geographic biases.

***Our answer****: Although we agree that this would be an interesting addition, this was out of the scope of the manuscript and would require data extraction from each of the thousands of articles on the different turtle species. All of these data extractions were conducted by hand, so this would add a massive amount of work. A future study using AI might bring this information more easily.*

2- A comparison between publication count and conservation funding per species or region, if data are available.

***Our answer****: We also agree that this would be a welcome future study, though this was out of the scope of our study. We thank the reviewer for this suggestion which we added as a perspective in the discussion (see lines 528-530).*

Conclusion:
This manuscript has the potential to make a meaningful contribution to reptile conservation and bibliometric ecology, but it requires significant revisions to improve its methodological rigor, analytical clarity, and conservation relevance.
Please revise the manuscript accordingly and provide a detailed point-by-point rebuttal.

***Our answer****: We are grateful to the reviewer for constructive comments that helped us improving the quality of our study.*

Reviewer #2: This is an interesting contribution with implications not only for the turtles, but for the approach to biodiversity studies as a whole. I need to stress that I am not an expert in statistics, so those should be checked by a specialist in the field. However, I can provide some insights from the general biological and herpetological point of view. Overall, I think the paper is well-written and it should be published but I see some issues, mostly with how the results are presented, as I think that the potential of the data is not fully utilized and some aspects may require slight refinement or a better justification.

***Our answer****: We thank the reviewer for comments that helped improving the manuscript.*

The topic is somewhat difficult from the methodological standpoint because, given the vast number of published papers, it is not really possible to review their contents manually, whereas any semiautomatic approach (such as the one employed by the Authors) will unavoidably result in some errors. For example, some papers providing valuable information on some species may be overlooked, because they do not include the full name of the species in any of the fields searchable by the Web of Science, but instead use, e.g., only the generic name or the name of the family (as I understand, the Authors did not look for those, only for the binomina). On the other hand, in some cases a specific name may be used in an abstract, e.g., solely in a comparative context without any meaningful information on that species presented in the paper (false hits), or as a synonym (double hits when both variants of a name are searched for). I understand that these instances are inevitable and while some workarounds can be proposed, they cannot be fully eliminated. I also understand that the cutoff used by the Authors (1975) is a sad necessity due to the increasingly limited availability of data for processing (that could be, to some extent, helped by inclusion of, e.g., the Biodiversity Heritage Library) but also due to complications regarding the synonymy and more frequent usage of sole generic names in those older papers. Unfortunately, this leaves out some important anatomical and ecological literature, also regarding the less popular taxa, which is still useful and informative and which in some cases may render restudy of those species in modern times less of a priority, thus causing them to appear even less studied in the selected timeframe. This should be perhaps acknowledged slightly more in the text (although the Authors already referred to that problem) but other than that we cannot do much about it and I accept that as an inherent limitation of that kind of research.

***Our answer****: As mentioned in our response to the editor and reviewer 1, we developed the discussion on this issue, although we already discussed it in both the methods and discussion of the previous version of the MS (see lines 139 to 164 and 531 to 555). As mentioned in the manuscript as well as in response to reviewer 1, the objective of this research index is not to provide an absolute metric for species research effort, but an index that allows to compare species. There is a lot more literature on turtles that is not included in WOS or Scopus, and conversely, some articles that came up in our search did not necessarily focus on the species of interest (even if the species name is given in the abstract, it does not systematically imply that the article is focused on the species of interest). Our purpose was however not to produce an exact and exhaustive list of articles per species, but rather to compare the relative amount of research that has been devoted to each species. Although pre-1975 research can be very important and should not be neglected by researchers working on particular species, the amount of research articles that have been produced since 1975 is overwhelmingly larger, by multiple orders of magnitude. This increase in the quantity of research conducted since the 1970 has been described elsewhere in details (see also our response to another comment of reviewer 2). So even if it is possible that some species that were under studied since 1975 were the focus of several studies before that date, it would likely only slightly alter their score, and be of very little effect on the global trends that we aimed at testing here. We have now added more discussion on this aspect lines 535-547.*

One oversight and potential problem I see with communicating the methodology is related to Fig. 1. It presents the number of review papers on turtles. Unfortunately, I don’t see any notion of such a search in the Methods section and the methodology for compiling that charts seems to be only explained in the figure caption. However, this does not seem right for two reasons. (1) using solely the given query (“tortoise*” OR “chelonian*” OR “turtle*” OR “testudines*”) one is expected to find not only papers relevant to the reviewed manuscript, but potentially also papers completely out of scope, e.g., on extinct stem turtles, turtle-derived materials (tortoiseshell), arthropods (tortoise beetles), etc., so the result does not really say much. (2) I tried to see how badly out of scope the results are in that case, but I could not replicate the Author’s results. I searched for the provided keywords, string copied directly from the figure caption, and document type set to Review Article. I will give the values for the year 2022, because this is the largest number in the Author’s search and shows the problem the most easily, but there were also differences for other years. When searching in Author Keywords, 21 articles were found. This is much below the over 25 papers found by the Authors for that year. When searching in Keywords plus, the search yielded 58 papers for that year – this is much more. Also, as expected, the search found lots of papers on fossil turtles and some nonsensical ones (e.g., “Plant phenolics: neglected secondary metabolites in plant stress tolerance”). Please check and preferably refine – the Author Keywords yielded more sensible results in that case and about 250 papers total, so it is doable to leave out those that are out of scope). In case the Authors searched in Keywords Plus and manually rejected results out of scope (possible but I think unlikely, as that search produced over 700 results for me) then kudos to them but please communicate that in the Methods!

***Our answer****: We have now removed this figure, which was only meant to provide a simple illustration on the increase in reviews and large taxonomic scale comparative studies on the Testudinae group to further illustrate the need for our study, in the introduction. This is why we did not mention this approach in the methods, but instead directly in the introduction. Our purpose was not to be exhaustive, but to show the relative increase in these large-scale studies that sometimes rely on the expectation that all species are equally known, or that aim at providing patterns or mechanisms that are true at the scale of the entire clade. Why the reviewer did not find the same numbers as us is unclear to us, as we reran these searches and found very similar numbers for each year. Note however that the WoS database is frequently changed, with new references added or removed regularly, which may explain this discrepancy (though to our experience, the differences are usually minor). We removed the figure to avoid any misunderstanding with regards to the interpretation of the figure, and because it was not absolutely needed.*

The most important aspect, which is currently very indirectly and tangentially tackled by the Authors but certainly requires more attention as a potential good predictor of research effort, is simply whether the species in question is native to territories of more scientifically active countries (e.g., with better financing of scientific institutions). This may very well explain why some taxa became sort of model species for turtles and why they continue to gain attention while other remain neglected. Given the huge differences in research effort between species, some receiving next to no attention and some literally thousands of papers, I suspect that such geopolitical factors may not only be significant, but even overpower other traits – especially that, if I see correctly, all but one species with over 150 papers on their account occur either in the USA, Western or Southern Europe, or China, whereas none of at least several dozens of the least studied species (except of some artificially underestimated instances – see below) occurs there. Currently, the Authors only briefly consider that topic in terms of generalized geographic regions (North America, Central America, South America, etc.) but I am afraid this misses a lot of nuance, especially that a number of taxa falls into the uninformative “Multiple” category. Analyzing whether there is a statistically significant correlation between the research interest and the overlap of the species range with the territory of one or more of, let’s say, the 10 or 20 most scientifically active countries may prove extremally interesting and should not require a lot of additional work and may explain why some taxa inhabiting the same large-scale geographic area differ significantly in attention. I propose that the Authors check and discuss whether this is indeed the case.

***Our answer****: We followed the reviewer’s advice and compared the research effort of the Testudinae species occurring the 10 or 20 most scientifically active species, using a research ranking score per country, and ranking each species according to whether or not it occurs within the top 10 or top 20 countries. As predicted by the reviewers, there is a strong discrepancy, with species occurring in the most scientifically active countries being also the focus of more research. We now include this variable in both a univariate and multivariate models, though it was not a significant predictor in the multivariate model (likely because it is redundant with the geographic region that was already included). A diversity of socio-economic predictors are likely to affect research effort, including factors related to the cultural and economic importance of some taxa; other works have focused on these types of predictors to understand their role (though to the best of our knowledge, this has not been investigated in turtles). In this study, however, our purpose was not to test these questions in depth, nor to disentangle all drivers of variation in research effort. Instead, we were mostly interested in underlining the existence of research-biases across Testudinae species, and of their potential consequences for the interpretation of large scale comparative analyses. Elucidating all the causes of research-biases was out of the scope of our study.*

Additionally, the Authors should address the problem of recently established species, such as Kinosternon steindachneri (2013) and Sternotherus intermedius (2017), which did not yet accumulate a significant bibliography of their own, but were considered historically as part (subspecies or subpopulation) of their closely related species (in the case of Kinosternon steindachneri – Kinosternon subrubum) or hybrids (in the case of Sternotherus intermedius). As a result, true research effort devoted to them is either difficult to establish or artificially underestimated, in turn possibly skewing indices for corresponding supraspecific taxa and traits. If possible, in those instances it would be advisable to track the research effort on the subspecies rather than species level. Otherwise, they should probably be either considered jointly with their sister species or left out, unless their impact is confirmed to be insignificant.

***Our answer****: We agree that a limitation of our index of research effort is that it does not give the “true” research effort value for each species. It was however not our purpose, as we aimed at obtaining a relative index comparable across species (see our answer to previous comments). In the case of recently established species, if they were recently split and previously considered as a subspecies of other species, the subspecies name was included in the list of synonyms, and therefore considered (based on synonymy) even if the species was only recently recognized as a full species. Note that tracking subspecies research effort is particularly challenging as most studies do not precise which subspecies is investigated, but only provide the species name.*

Another thing that may deserve reconsideration on the part of the Authors is the usage of full decades in some of their analyses, despite their timeframe starting and ending in the middle of the decades (1975–2024). Five-year bins may perhaps be more adequate?

***Our answer****: Our purpose here was to test for changes in research effort discrepancy over time. Since the split into decades was sufficient to test and detect this effect, we decided not to include the five-years steps which would require to rerun every search species by species for each 5-year period, with no clear extra information for our understanding of variation in research effort across time.*

Finally, the paper is mostly built on the taxonomical framework established by the 2021 ninth edition of the Turtles of the World checklist. However, a new edition (tenth, 2025) is already available, so the manuscript should be updated to acknowledge that. The new edition distinguishes 359 extant species instead of 357 recognized by TotW 2021. Whether those new species should be considered on their own depends on the approach (see above), but a lot of attention has been given to turtle extinctions in the new volume, so it is relevant at least in that context.

***Our answer****: We have now updated our dataset with this 2025 edition of the checklist and reran all analyses considering this new framework (we also updated the historic range size values). Note that this new checklist was published on July 25^th^, 2025 – so after we’d submitted the previous version of the manuscript. Note that we also updated the scores of extinction risk and population trend for all species using the newly published IUCN red list (i.e., the 2025-2 version, from October 2025).*

More detailed notes are provided below:
P. 3, lines 43–44 („Testudines emerged during the Triassic and survived two mass extinction crises that eliminated several other groups of reptiles at the end of the Permian and Cretaceous”): This statement is incorrect for two reasons. First, the Testudines are the crown group composed from two extant clades: the side-necked turtles (Pleurodira) and hidden-necked turtles (Cryptodira). The exact time of divergence or those two clades (and, in consequence, the appearance of the Testudines) is somewhat contentious, as it depends on the classification of some poorly known fossil taxa, but it was certainly later than in the Triassic, most probably in the Middle to Late Jurassic. All Triassic turtles are stem taxa so they belong to Testudinata, not Testudines. Second, the Permian was before the Triassic, so neither the Testudines nor the Testudinata (as a group appearing at the end of the Triassic) could not survive the Permian extinction. I imagine that the Authors may confuse here the Testudines, Testudinata, and Pan-Testudines/Pantestudinata (a larger group including also shell-less turtle relatives, possibly also the Permian Eunotosaurus africanus), but these not the same and the paper focuses solely on Testudines proper, so there is no need to bring those earlier forms up.

***Our answer****: We thank the reviewer for this important point, apologize for our mistake and removed this sentence from the introduction.*

P. 3, lines 48 and 87 („Squamates”, “Amphibians”): when anglicized, the name should not be capitalized (so „Squamata” and “Amphibia” would be correct but in that case „squamates” and “amphibians”).
***Our answer****: Changed accordingly.*

P. 4, lines 104–105 (“fast lifestyle”, “slow lifestyle”): this seem somewhat poorly fitting terms, maybe “life cycle”?

***Our answer****: We changed these into fast and slow life history strategies (see lines 103-104).*

P. 6, line 186 (“maximum standard carapace length”): I am unfamiliar with such a measurement and I think the Authors mean here the “maximum straight-line carapace length” (a common measurement used for turtles and typically abbreviated as SCL, also used by Rhodin et al., 2021). If so, please also correct elsewhere. If not, please define.

***Our answer****: The reviewer is right and we changed this (into “straight-line carapace length”) throughout the manuscript.*

P. 8, line 313: can this increase be partially explained by the incomplete sampling of older articles in the WoS, e.g., published in historical but now discontinued journals which are not indexed?
***Our answer****: page 8 line 313 of the previous version of the MS refers to results showing the proportion of variance in research effort explained by taxonomy. This comment is likely referring to the previous paragraph and page, which shows the increase in research effort across time (lines 305-309)? If so, the increase in the number of publications across time is known and well documented globally (not only in biology) and was expected for Testudines research as well. We decided not to discuss this question in our manuscript is this was out of the scope of our study, and it has been the focus of diverse studies. Note that discontinued journals are also included in the WoS or Scopus databases. A few examples of work showing and discussing the causes of this global increase:*

[*https://direct.mit.edu/qss/article/5/4/823/124269/The-strain-on-scientific-publishing*](https://direct.mit.edu/qss/article/5/4/823/124269/The-strain-on-scientific-publishing)

# *Thekwall and Sud 2022 Scopus 1900–2020: Growth in articles, abstracts, countries, fields, and journals.* Quantitative Science Studies *(2022) 3 (1): 37–50.*

*Fire and Guestrin 2019 Over-optimization of academic publishing metrics: observing Goodhart's Law in action.* GigaScience*, Volume 8, Issue 6, giz053*

P. 10, line 401 (“Trachemys scrypta”): Trachemys scripta.

***Our answer****: Changed accordingly.*

Table 1: I think I understand what the Authors want to show here, but at the same time there is something missing. The juxtaposition of the taxonomic richness of the family and the number of papers is some information, but this does not say too much without some measure of the evenness of coverage. As presented, the Chelydridae are in the third spot with a decent 243 papers per species, which looks very good, but what of it, if nearly 90% of that (!) is devoted to one species. Conversely, the Geoemydidae only have 16 papers per species, so they appear significantly understudied compared to the chelydrids, but in reality the research effort is much more evenly distributed. In consequence, I feel the message here falls into the same pitfall which the Authors try to solve in their manuscript – we see an averaged picture but completely miss how some species are understudied. Also, the numbers alone are difficult to visualize, so the nice data that the Authors gathered get somewhat lost. I would strongly suggest presenting this in some more visual way. Perhaps a bar chart with the bars subdivided into sections corresponding to the proportional research attention received by individual species, or a pie chart with subdivided slices? I get it that the number of species within many families is too large to show all of them within a single bar/slice, but the differences in attention between them are large enough that the best studied species within each family should be visible as individual portions, whereas the least studied species may be grouped together. This would not only nicely show the differences between the families, but it would also present the disproportions in the effort within each of them and it would also be much more visually appealing.

***Our answer****: We thank the reviewer for this suggestion. We have now added to this table the standard error for each family as an index of research effort evenness across species within families. We also followed the reviewer advice by adding a new figure with a boxplot showing the distribution of research effort within each family, with the four quartiles, mean, median and number of species per family (see Figure 2).*

Fig. 1 caption: “Evolution” is not a good term here, I would suggest just leaving “The number of review articles” etc.

***Our answer****: We have now removed this figure from the manuscript.*

Figs 4 and 5: Please explain what do “a”, “b”, and “c” mean.

***Our answer****: We now provide this information in both captions.*

*We are very grateful to Tomasz Szczygielski for constructive comments that helped us to improve our manuscript.*
