# Supplementary material for: Which turtle should I study? Uneven distribution of research effort across Testudines species
Source: PLoS One. 2026 Apr 20;21(4):e0347198. doi: 10.1371/journal.pone.0347198 (PMC13094973; doi:10.1371/journal.pone.0347198)
Supplement: Attachment — Submitted filename: Comments_Editor.docx [file pone.0347198.s007.docx]

**Comments to the editor**

Thank you for the opportunity to review the revised version of this manuscript. The authors have made substantial and meaningful improvements in response to both reviewers’ comments from the first round. In particular, the updated taxonomy (Rhodin et al. 2025, now covering 370 species), the addition of the country research rank variable, the parallel analyses with and without sea turtles, and the transparent resolution of the clutch size sign reversal are all commendable.

I am broadly satisfied with the revisions and believe the manuscript makes a useful contribution to our understanding of research biases in chelonian biology. My recommendation is minor revision. The remaining issues are largely editorial in nature, though two substantive points merit the authors’ attention.

First, a wording error on line 326, which persists from the original submission, reverses the direction of a key finding (stating that marine turtles attracted 18.1 times more research in the 1980s compared to the 2010s, when the intended meaning is clearly the opposite). Second, the multivariate model is based on 115 of 370 species (31%), and species with complete trait data are likely to be better-studied species, creating a potential circularity that should be briefly acknowledged.

Full comments to the authors are attached separately.
