# Supplementary material for: Which turtle should I study? Uneven distribution of research effort across Testudines species
Source: PLoS One. 2026 Apr 20;21(4):e0347198. doi: 10.1371/journal.pone.0347198 (PMC13094973; doi:10.1371/journal.pone.0347198)
Supplement: Attachment — Submitted filename: Comments_authors.docx [file pone.0347198.s008.docx]

**Comments to the authors**

The authors have done commendable work in revising this manuscript. The revisions are substantial and demonstrate a genuine engagement with the concerns raised by both reviewers and the editor. In particular, the updated taxonomy (2025 Turtles of the World checklist, now covering 370 species), the addition of the country research rank variable to address geopolitical bias, the parallel multivariate analyses with and without sea turtles, and the resolution of the clutch size sign reversal through its exclusion from the multivariate model are all welcome improvements. The removal of the problematic review papers figure and the correction of the Triassic/Testudines error also strengthen the manuscript. The paper reads more clearly and is better supported than the first submission.

That said, a small number of issues remain, which I outline below. Most are minor and should be straightforward to address. I believe the manuscript is approaching publication readiness, and I would recommend minor revisions before acceptance.

**Substantiative points**

1. **Wording error on line 326 (persists from the previous version).** The sentence currently reads: "This increase was particularly important in marine turtles, which attracted 18.1 times more research in the 1980s as compared to the 2010s." The direction is reversed. Given the context and Figure 1, the intended meaning is clearly that the 2010s saw 18.1 times more research on marine turtles than the 1980s, not the other way round. This should be corrected to read something like: "...which attracted 18.1 times more research in the 2010s as compared to the 1980s."
2. **Potential bias in the multivariate model subset.** The final multi-predictor model (Table 3) is based on 115 species for which all trait values were available, out of 370 total. This represents roughly 31% of all Testudines species. The concern here is that species with complete trait data are more likely to be well-studied species, since trait values are typically documented through the very research effort the model is trying to explain. This creates a potential circularity: the model may primarily be explaining variation among the already better-known species, and the predictors that emerge as significant may not generalise to the full dataset.

I recognise that this is a common limitation of comparative studies relying on trait data, and that the authors have already taken steps to maximise sample size (e.g. by excluding female age at maturity and generation length). Nevertheless, I would encourage the authors to briefly acknowledge this potential circularity in the Discussion, or alternatively, to provide a simple comparison of mean research effort between species included versus excluded from the multivariate model to quantify the extent of this bias.

**Minor points**

3. **32 zero-publication species.** The increase from 29 to 32 species with no publications (line 322) following the taxonomy update is a striking finding. I would encourage the authors to include the full list of these 32 species as a supplementary table. This would be a valuable resource for the conservation community and would cost very little in terms of additional effort.
4. **Country research rank variable.** The addition of this variable (lines 185–196) is a welcome response to Reviewer 2’s suggestion, and the results are illuminating: species in the top 10 countries had a mean research effort of 145 articles versus 9 for species absent from the top 20 (lines 354–358). It is interesting, though perhaps not surprising, that this variable was not retained in the multivariate model, likely due to redundancy with the biogeographic realm. The authors note this (response to Reviewer 2), and a brief mention in the Discussion of why these two variables are partially redundant would be useful for the reader.
5. **Hypothesis framing.** The Introduction continues to frame the research questions as open-ended queries (lines 102–106) rather than as directional predictions with stated rationale. I appreciate that this is an exploratory study and that PLOS ONE’s scope accommodates this approach. Nevertheless, even a brief statement of expected patterns (e.g. “We predicted that species occurring in regions with greater research infrastructure would attract more attention, given...”) would sharpen the framing and strengthen the reader’s ability to evaluate the findings against clear expectations. This is offered as a suggestion rather than a requirement.

**Summary**

This is a useful and well-executed study that makes a meaningful contribution to our understanding of research biases in Testudines, with clear implications for conservation prioritisation and the interpretation of large-scale comparative analyses. The authors have been responsive to the first round of reviews and have substantially improved the manuscript. The remaining issues are relatively minor. I recommend that the wording error on line 326 be corrected, and that the authors consider briefly addressing the potential bias in the multivariate model’s species subset. The other suggestions above are offered in the spirit of strengthening an already solid piece of work.

**Recommendation:** Minor revision
